# Density of rational points on some quadric bundle threefolds

**DOI:** 10.1007/s00208-024-02854-4

**Published:** 2024-04-15

**Authors:** Dante Bonolis, Tim Browning, Zhizhong Huang

**Affiliations:** 1 Department of Mathematics, Duke University, Durham, NC 27708 USA; 2 IST Austria, Am Campus 1, 3400 Klosterneuburg, Austria; 3 Institute of Mathematics, Chinese Academy of Sciences, Beijing, 100190 China

**Keywords:** 11D45 (11G35, 11G50, 11P55, 14G05, 14G25)

## Abstract

We prove the Manin–Peyre conjecture for the number of rational points of bounded height outside of a thin subset on a family of Fano threefolds of bidegree (1, 2).

## Introduction

The primary purpose of this paper is to resolve the Manin–Peyre conjecture for a family of smooth Fano threefolds in ${{\mathbb {P}}}^1\times {{\mathbb {P}}}^3.$ Let $L_1,\dots ,L_4\in {{\mathbb {Z}}}[x_1,x_2]$ be binary linear forms which are pairwise non-proportional. Let $V\subset {{\mathbb {P}}}^1\times {{\mathbb {P}}}^3$ be given by

1.1 $$\begin{aligned} L_1(x_1,x_2)y_1^2+L_2(x_1,x_2)y_2^2+L_3(x_1,x_2)y_3^2+L_4(x_1,x_2)y_4^2=0, \end{aligned}$$

defining a smooth Fano threefold of bidegree (1, 2). The Picard group is ${{\,\textrm{Pic}\,}}(V)\cong {\mathbb {Z}}^2$ and *V* is equipped with two morphisms, corresponding to the projections $\pi _1:{{\mathbb {P}}}^1\times {{\mathbb {P}}}^3\rightarrow {{\mathbb {P}}}^1$ and $\pi _2:{{\mathbb {P}}}^1\times {{\mathbb {P}}}^3\rightarrow {{\mathbb {P}}}^3$. Let $|\cdot |$ denote the sup-norm on ${\mathbb {R}}^d$, for any $d\in {\mathbb {N}}$. We can associate an anticanonical height function on $V({\mathbb {Q}})$ via $H(x,y)=|\textbf{x}||\textbf{y}|^2,$ if $(x,y)\in V({\mathbb {Q}})$ is represented by a vector $(\textbf{x},\textbf{y})\in {\mathbb {Z}}_{\text {prim}}^2\times {\mathbb {Z}}_{\text {prim}}^4$. The Manin–Peyre conjecture [10, 20, 21] predicts that there should exist a thin subset $\Omega \subset V({\mathbb {Q}})$, in the sense of Serre [24, §3.1], such that

1.2 $$\begin{aligned} \#\{(x,y)\in V({\mathbb {Q}})\setminus \Omega : H(x,y)\leqslant B\} \sim c_V B\log B, \end{aligned}$$

where $c_V$ is a constant whose value has been conjectured by Peyre [20]. Addressing a question of Peyre, raised in his lecture at the 2009 conference *Arithmetic and algebraic geometry of higher-dimensional varieties* at the University of Bristol, numerical evidence towards this conjecture has been supplied by Elsenhans [9]. We can write (1.1) as

1.3 $$\begin{aligned} x_1Q_1(y_1,\dots ,y_4)=x_2Q_2(y_1,\dots ,y_4), \end{aligned}$$

for suitable diagonal quadratic forms $Q_1,Q_2\in {\mathbb {Z}}[y_1,\dots ,y_4]$. Consider the subvariety $Z\subset {\mathbb {P}}^3$ given by $Q_1=Q_2=0.$ Our assumptions on $L_1,\dots ,L_4$ ensure that *Z* is a smooth genus one curve and so the closed subvariety $\pi _2^{-1}(Z)\cong {{\mathbb {P}}}^1\times Z \subset V$ defines an elliptic cylinder. If $Z({\mathbb {Q}})\ne \emptyset$ then there are $\gg B^2$ rational points of anticanonical height $\leqslant B$ on $\pi _2^{-1}(Z)$. Thus, in general, we should certainly demand that $\Omega$ contains $\pi _2^{-1}(Z)({\mathbb {Q}})$.

The restriction of $\pi _1$ to *V* gives a fibration into quadrics $\pi _1:V\rightarrow {\mathbb {P}}^1$. If $x\in {\mathbb {P}}^1({\mathbb {Q}})$ is represented by $\textbf{x}=(x_1,x_2)\in {{\mathbb {Z}}}_{{\text {prim}}}^2$, then $\pi _1^{-1}(x)$ is split if and only if

1.4 $$\begin{aligned} \prod _{i=1}^{4}L_i(\textbf{x})=\square . \end{aligned}$$

For such a point *x* with $\pi _1^{-1}(x)({\mathbb {Q}})\ne \emptyset$, the fibre will contribute $\sim c_x B\log B$ points, for an appropriate constant $c_x>0$ that depends on *x*. Based on numerical investigation, Elsenhans suggests that the conjectured asymptotic (1.2) holds when $\Omega$ is taken to be union of $\pi _2^{-1}(Z)({\mathbb {Q}})$ and the set of $(x,y)\in V({\mathbb {Q}})$ for which (1.4) holds. In particular, $\Omega \subset V({\mathbb {Q}})$ is a thin set of rational points, since $\pi _2^{-1}(Z)({\mathbb {Q}})$ lies on a proper subvariety of *V* and the set of rational points satisfying (1.4) correspond to rational points on a double covering. Note that (1.4) defines an elliptic curve $E\subset {\mathbb {P}}(2,1,1)$ and so $\Omega$ is Zariski dense in *V* if *E* has positive rank. In fact, as discussed by Skorobogatov [25, § 3.3], *E* is the Jacobian of the genus 1 curve *Z*. An explicit example is given by taking *V* to be

1.5 $$\begin{aligned} x_1y_1^2+x_2y_2^2+(x_1+2x_2)y_3^2+(x_1+6x_2)y_4^2=0, \end{aligned}$$

for which $Z({\mathbb {R}})=\emptyset$ and $E({\mathbb {Q}})\cong ({\mathbb {Z}}/2{\mathbb {Z}})^2 \times {\mathbb {Z}}$ (with Cremona label 192a2). Our main result settles the thin set version of the Manin–Peyre conjecture under mild assumptions on *V*.

### Theorem 1.1

Assume that $L_1,\dots ,L_4\in {{\mathbb {Z}}}[x_1,x_2]$ are pairwise non-proportional linear forms, each with coprime coefficients, such that $Z({\mathbb {R}})=\emptyset$. Then, if $\Omega$ is the set of $(x,y)\in V({\mathbb {Q}})$ for which (1.4) holds, we have

$$\begin{aligned} \#\{(x,y)\in V({\mathbb {Q}})\setminus \Omega : H(x,y)\leqslant B\} \sim c_V B\log B, \end{aligned}$$

where $c_V$ is the constant predicted by Peyre [20].

The example (1.5) satisfies the assumptions of the theorem. With further work, it is possible to remove the hypotheses that each $L_i$ has coprime coordinates and $Z({\mathbb {R}})=\emptyset$. The latter is a particularly convenient assumption that allows us to assume that the regions we work in are not too lopsided. Since these hypotheses simplify an already lengthy argument, we have chosen not to attempt their removal here.

As discussed in [16], a geometric framework for identifying problematic thin sets in the Manin–Peyre conjecture has been developed by Lehmann, Sengupta and Tanimoto. In private communication with the authors, Professor Tanimoto has indicated that similar arguments to those in [16, § 12] show that the thin set $\Omega$ in Theorem 1.1 agrees with their prediction.

Manin [17] used height machinery to establish a lower bound supporting linear growth for all smooth Fano threefolds, possibly after an extension of the ground field. More recently, Tanimoto [26] has produced a range of upper bounds for various classes of Fano threefolds, but his work does not cover (1.1). The classification of Fano threefolds with Picard number 2 goes back to Mori and Mukai [18], but it is convenient to appeal to the summary of Iskovskikh and Prokhorov [15, Table 12.3]. Over $\overline{{\mathbb {Q}}}$, there are 36 isomorphism types of Fano threefold of Picard number 2. The expectation is that the arithmetic of these varieties becomes harder the higher up the table they appear. As explained in Remark (i) before [15, Table 12.3], varieties numbered 33–36 are toric. Thus, for each of these, the Manin–Peyre conjecture follows from work of Batyrev and Tschinkel [1]. Equivariant compactifications of the additive group ${\mathbb {G}}_a^3$ are also known to satisfy the Manin–Peyre conjecture, thanks to work of Chambert-Loir and Tschinkel [8], and the smooth Fano threefolds that arise as equivariant compactifications of ${\mathbb {G}}_a^3$ have been identified by Huang and Montero [14]. These cover varieties numbered 28, 30, 31 and 33–36. Variety number 32 is a bilinear hypersurface in ${\mathbb {P}}^2\times {\mathbb {P}}^2$, which is a flag variety and so covered by [10]. Finally, in a recent tour de force [2], Blomer, Brüdern, Derenthal and Gagliardi have shown that the Manin–Peyre conjecture holds for many spherical Fano threefolds of semisimple rank one. Among those of Picard number 2, this covers a quadric in ${\mathbb {P}}^4$ blown-up along a conic, which corresponds to variety number 29 in [15, Table 12.3]. Our variety $V\subset {\mathbb {P}}^1\times {\mathbb {P}}^3$ has Picard number 2 and can be viewed as the blow-up of ${{\mathbb {P}}}^3$ along the genus one curve *Z*, corresponding to variety number 25. In particular it is neither spherical, nor toric, nor an equivariant compactification of ${\mathbb {G}}_a^3$.

The proof of Theorem 1.1 is inspired by recent work by Browning and Heath-Brown [5], which resolves the Manin–Peyre conjecture for the Fano fivefold

1.6 $$\begin{aligned} x_1y_1^2+ x_2y_2^2+ x_3y_3^2+ x_4y_4^2=0, \end{aligned}$$

of bidegree (1, 2) in ${\mathbb {P}}^3\times {\mathbb {P}}^3$. In this setting, an anticanonical height is $|\textbf{x}|^3|\textbf{y}|^2$, if (*x*, *y*) is represented by $(\textbf{x},\textbf{y})\in {\mathbb {Z}}_{\text {prim}}^4\times {\mathbb {Z}}_{\text {prim}}^4$. The basic line of attack in [5] involves counting points as a union of planes when $|\textbf{y}|\leqslant B^{\frac{1}{4}}$, and as a union of quadrics when $|\textbf{x}|\leqslant B^{\frac{1}{6}-\eta }$, for any fixed $\eta >0$. In the first case, geometry of numbers arguments are used to count the relevant vectors $\textbf{x}$, whereas the circle method underpins the second case. In terms of the inequality $|\textbf{x}|^3|\textbf{y}|^2\leqslant B$, this leaves a small range uncovered, for which it is necessary to have an upper bound of the correct order of magnitude.

Our work follows a similar strategy, but with substantial extra difficulties. This is reflected in the geometry of the effective cone of divisors ${{\,\textrm{Eff}\,}}_V$. Let $H_1=\pi _1^{*}{{\mathscr {O}}}_{{\mathbb {P}}^1}(1)$ and $H_2=\pi _2^{*}{{\mathscr {O}}}_{{\mathbb {P}}^3}(1)$ be hyperplane classes. As discussed by Ottem [19, Thm. 1.1], the effective cone is ${{\,\textrm{Eff}\,}}_V= {{\mathbb {R}}}_{\geqslant 0}H_1+{{\mathbb {R}}}_{\geqslant 0}C$, where $C=-H_1+2H_2$ is the class of the exceptional divisor $\pi _2^{-1}(Z)$. This is larger than the nef cone ${\text {Nef}}_V={{\mathbb {R}}}_{\geqslant 0} H_1+{{\mathbb {R}}}_{\geqslant 0} H_2$, meaning that ${{\,\textrm{Eff}\,}}_V^{\vee }$ is smaller than ${\text {Nef}}_V^\vee$, which strongly influences the asymptotic behaviour in Theorem 1.1. This is in stark contrast to the situation in (1.6), where the effective cone is equal to the nef cone. When $|\textbf{x}|\leqslant B^{\frac{1}{4}}$, we view *V* as a family of quadrics via (1.1). We will then reapply the circle method arguments worked out in [5], but we shall face extra challenges in dealing with a family over ${\mathbb {P}}^1$ rather than over ${\mathbb {P}}^3$. When $|\textbf{x}|\geqslant B^{\frac{1}{4}+\eta }$, we view *V* as being given by (1.3), which we can use to eliminate $x_1$ and $x_2$, on extracting common divisors. This ultimately leads to a counting problem of the form

$$\begin{aligned} \sum _{d\leqslant D} \#\left\{ \textbf{y}\in {\mathbb {Z}}_{\text {prim}}^4: |\textbf{y}|\leqslant Y, ~Q_1(\textbf{y})\equiv Q_2(\textbf{y}) \equiv 0 \bmod {d}\right\} , \end{aligned}$$

for $D,Y\geqslant 1$. On interpreting the inner sum as a disjoint union of lattice conditions, the main work is to show that the successive minima have the expected order of magnitude, as one averages over *d* and over the different lattices. This counting problem is rather different to the one appearing [5], and it seems likely the methods developed could be useful in the study of other quadric bundles, including del Pezzo surfaces with a conic bundle structure. It is in this part of the argument that the difference between ${{\,\textrm{Eff}\,}}_V$ and ${\text {Nef}}_V$ manifests itself. If $|\textbf{x}|\geqslant B^{\frac{1}{4}+\eta }$ and $|\textbf{x}||\textbf{y}|^2\leqslant B$, then we are only interested in the range $|\textbf{y}|\leqslant B^{\frac{3}{8}-\frac{\eta }{2}}$. However, it turns out that the contribution from $|\textbf{y}|\leqslant B^{\frac{1}{4}}$ is negligible, which thereby reduces the size of the leading constant. In (1.6), by comparison, the contribution from $|\textbf{y}|\leqslant B^\delta$ makes a positive proportional contribution for any $\delta >0$.

Finally, we are left with a small range to cover via an auxiliary upper bound of the correct order. The completely diagonal structure of (1.6) renders it easier to obtain the necessary upper bound via a modification of Hua’s inequality. Lacking this diagonal structure, our approach involves an array of inputs, from character sum estimates and point counting on *Z* modulo prime powers, to various circle method applications [3, 5] and a general upper bound for the number of rational points of bounded height on diagonal quadric surfaces [4].

## Roadmap of the proof

We proceed to summarise some of the steps in the proof of Theorem 1.1. We shall frequently switch between the representation of *V* given by (1.1), involving the pairwise non-proportional linear forms $L_1,\dots ,L_4\in {{\mathbb {Z}}}[x_1,x_2]$ with coprime coefficients, and the representation (1.3), involving diagonal quadratic forms $Q_1,Q_2\in {\mathbb {Z}}[y_1,\dots ,y_4]$ such that $Z({\mathbb {R}})=\emptyset$. All of the estimates in our work are allowed to depend implicitly on the coefficients of the polynomials $L_1,\dots ,L_4$. Any other dependence will be indicated by an appropriate subscript.

For each $$1\leqslant i\leqslant 4$$, we may assume that $$ L_i(x_1,x_2)=a_ix_1+b_ix_2, $$ for $$a_i,b_i\in {\mathbb {Z}}$$ such that $$\gcd (a_i,b_i)=1$$. Define the set of primes $$ {\mathscr {P}}=\{p: p\mid (a_{i}b_{j}-a_{j}b_{i})\text { for some } i\ne j\}\cup \{2\}. $$ Then $${\mathscr {P}}$$ is a finite set, since $$L_i$$ and $$L_j$$ are assumed to be non-proportional, for distinct $$1\leqslant i,j\leqslant 4$$. It will be convenient to set

2.1 $$\begin{aligned} \Delta =\prod _{p\in {\mathscr {P}}}p. \end{aligned}$$

Our diagonal quadratic forms take the shape

2.2 $$\begin{aligned} Q_{1}(\textbf{y})=\sum _{i=1}^{4}a_{i}y_{i}^{2},\quad Q_{2}(\textbf{y})=-\sum _{i=1}^{4}b_{i}y_{i}^{2}. \end{aligned}$$

Moreover, $Q_1=Q_2=0$ defines a smooth genus 1 curve $Z\subset {\mathbb {P}}^3$ such that $Z({\mathbb {R}})=\emptyset$.

Let $N_V(\Omega ,B)$ denote the counting function whose asymptotics we are trying to determine. We shall avoid the set $\Omega$ by stipulating that

2.3 $$\begin{aligned} \prod _{i=1}^{4}L_i(\textbf{x})\ne \square , \end{aligned}$$

for any rational point $(x,y)\in V({\mathbb {Q}})$ that is to be counted. On taking into account the action of the units in ${\mathbb {P}}^1({\mathbb {Q}})\times {\mathbb {P}}^3({\mathbb {Q}})$, we have

2.4 $$\begin{aligned} N_V(\Omega ,B)=\frac{1}{4}\#\left\{ (\textbf{x},\textbf{y})\in {{\mathbb {Z}}}_{{\text {prim}}}^2\times {{\mathbb {Z}}}_{{\text {prim}}}^4: \begin{array}{l} (1.1) \ \, \text {and} \ \, (2.3) \, \ \text {hold}\\ |\textbf{x}||\textbf{y}|^2 \leqslant B \end{array} \right\} . \end{aligned}$$

Following the line of attack in [5], we will use different techniques to estimate the size of $N_V(\Omega ,B)$, according to the relative sizes of $|\textbf{x}|$ and $|\textbf{y}|$. When $|\textbf{x}|$ is small, we will fix $\textbf{x}$ and use the circle method to estimate the number of $\textbf{y}$. In fact the relevant application of the circle method carried out in [5, § 4] is directly in a form that can be applied to our own setting. On the other hand, when $|\textbf{x}|$ is large, we will use (1.3) to eliminate $\textbf{x}$ and reduce to a problem about counting integer vectors which reduce to *Z* modulo *d*, for varying moduli *d*. There remains an annoying middle range which requires a sufficiently sharp upper bound.

Let

2.5 $$\begin{aligned} {\mathscr {M}}(X,Y)=\left\{ (\textbf{x},\textbf{y})\in {{\mathbb {Z}}}_{{\text {prim}}}^2\times {{\mathbb {Z}}}_{{\text {prim}}}^4: \begin{array}{l} (1.1)\ \text {and}\ (2.3)\ \text {hold}\\ X\leqslant |\textbf{x}|< 2X,~|\textbf{y}|\leqslant Y\end{array}\right\} , \end{aligned}$$

for $X,Y\geqslant 1$. Since $Z({\mathbb {R}})=\emptyset$ it is easy to deduce from the alternative representation (1.3) of (1.1) that ${\mathscr {M}}(X,Y)$ is empty unless $X\ll Y^2$ for a suitable implied constant depending only on *V*. In Sect. 5 we shall prove the following general upper bound for the cardinality of ${\mathscr {M}}(X,Y)$.

### Theorem 2.1

Let $$X,Y\gg 1$$. Then

$$\begin{aligned} \#{\mathscr {M}}(X,Y)\ll XY^2 + \min \left\{ X^2Y^{4/3}, X^{\frac{1}{4}}Y^{\frac{5}{2}} \frac{\log Y}{\log \log Y}\right\} . \end{aligned}$$

On breaking the ranges for $|\textbf{x}|$ and $|\textbf{y}|$ into dyadic intervals, Theorem 2.1 easily implies the optimal upper bound $N_V(\Omega ,B)=O( B\log B).$ In fact, not only does it help cover an awkward range for the relative sizes of $|\textbf{x}|$ and $|\textbf{y}|$, but certain steps in the proof of Theorem 2.1 also play a vital role in the proof of the asymptotic formula, where it used to show that certain lattices are rarely lopsided.

Let us now summarise the proof of Theorem 1.1 in a little more detail. Let

2.6 $$\begin{aligned} {{\mathscr {L}}}(B)= \left\{ (\textbf{x},\textbf{y})\in {{\mathbb {Z}}}_{{\text {prim}}}^2\times {{\mathbb {Z}}}_{{\text {prim}}}^4: \begin{array}{l} (1.1)\ \text {and}\ (2.3)\ \text {hold}\\ |\textbf{x}||\textbf{y}|^2\leqslant B \end{array} \right\} , \end{aligned}$$

so that $N_V(\Omega ,B)=\frac{1}{4}\#{{\mathscr {L}}}(B)$ in (2.4). We will decompose ${{\mathscr {L}}}(B)$ into three sets

2.7 $$\begin{aligned} {{\mathscr {L}}}(B)={{\mathscr {L}}}_1(B)\sqcup {{\mathscr {L}}}_2(B)\sqcup {{\mathscr {L}}}_3(B), \end{aligned}$$

where

$$\begin{aligned} {{\mathscr {L}}}_1(B)&=\left\{ (\textbf{x},\textbf{y})\in {{\mathscr {L}}}(B): B^{\frac{1}{4}+\eta }\leqslant |\textbf{x}|\leqslant B^{\frac{1}{2}-\eta } \right\} ,\\ {{\mathscr {L}}}_2(B)&=\left\{ (\textbf{x},\textbf{y})\in {{\mathscr {L}}}(B):|\textbf{x}|\leqslant B^\frac{1}{4}\right\} ,\\ {{\mathscr {L}}}_3(B)&=\left\{ (\textbf{x},\textbf{y})\in {{\mathscr {L}}}(B):B^\frac{1}{4}<|\textbf{x}|<B^{\frac{1}{4}+\eta } \text { or } |\textbf{x}|> B^{\frac{1}{2}-\eta } \right\} , \end{aligned}$$

for any $\eta >0$. The parameter $\eta$ will ultimately be taken to be arbitrarily small, but it is fixed at each appearance. We now reveal our estimates for the cardinality of these sets.

The treatment of $\#{{\mathscr {L}}}_1(B)$ rests on rewriting (1.1) as (1.3) and then appealing to the geometry of numbers. In order to record our result, we first need to define some auxiliary quantities. For any prime *p* and $a\in {\mathbb {N}}$, we can define an equivalence relation on $\left( {\mathbb {Z}}/p^a{\mathbb {Z}}\right) ^{4}$, by saying $\textbf{u}$ is equivalent to $\textbf{v}$ if and only if there exists $\lambda \in \left( {\mathbb {Z}}/p^a{\mathbb {Z}}\right) ^{\times }$ such that $\lambda \textbf{u}\equiv \textbf{v}\bmod {p^a}$. An important role will be played in our work by the set of equivalence classes

$$\begin{aligned} V_{p^a}^{\times }=\{\textbf{u}\in \left( {\mathbb {Z}}/p^a{\mathbb {Z}}\right) ^{4}: p\not \mid \textbf{u},~Q_{1}(\textbf{u})\equiv Q_{2}(\textbf{u})\equiv 0 \bmod {p^a}\}/\left( {\mathbb {Z}}/p^a{\mathbb {Z}}\right) ^{\times }. \end{aligned}$$

We may now define

2.8 $$\begin{aligned} \mathfrak {S}_1=\prod _{p}\left( 1-\frac{1}{p}\right) \left( 1-\frac{1}{p^4}+\left( 1-\frac{1}{p}\right) ^2\sum _{a=1}^{\infty }\frac{\#V_{p^{a}}^{\times }}{p^{2a}} \right) . \end{aligned}$$

The absolute convergence of $\mathfrak {S}_1$ is ensured by Corollary 3.6, which implies that

$$\begin{aligned} 1-\frac{1}{p^4}+\left( 1-\frac{1}{p}\right) ^2\sum _{a=1}^{\infty }\frac{\#V_{p^{a}}^{\times }}{p^{2a}} =1+\frac{1}{p}+O\left( \frac{1}{p^{\frac{3}{2}}}\right) , \end{aligned}$$

if $p\not \mid \Delta$. We can now state our first asymptotic formula, which will be the object of Sect. 6.

### Proposition 2.2

Let $\eta >0$. There exist absolute constants $c_2>c_1>0$ such that

$$\begin{aligned} \#{{\mathscr {L}}}_1(B)=2 \mathfrak {S}_1 B \int _{\begin{array}{c} \textbf{y}\in {\mathbb {R}}^4\\ c_1B^{\frac{1}{4}+\frac{\eta }{2}} \leqslant |\textbf{y}|< c_2B^{\frac{3}{8}-\frac{\eta }{2}} \end{array}} \frac{\textrm{d}\textbf{y}}{|\textbf{y}|^2\max (|Q_1(\textbf{y})|,|Q_2(\textbf{y})|)} +O_\eta \left( B\sqrt{\log B}\right) . \end{aligned}$$

Next, we show that $\#{{\mathscr {L}}}_2(B)$ can be estimated asymptotically, as $B\rightarrow \infty$. This will be achieved using the Hardy–Littlewood circle method. Let

2.9 $$\begin{aligned} \tau _\infty = \int _{-\infty }^\infty \int _{[-1,1]^6} {\text {e}}\left( \theta \left( L_1(\textbf{x})y_1^2+\cdots + L_4(\textbf{x})y_4^2\right) \right) \textrm{d}\textbf{x}\textrm{d}\textbf{y}\textrm{d}\theta \end{aligned}$$

and

2.10 $$\begin{aligned} A_q=\sum _{\begin{array}{c} a\bmod {q}\\ \gcd (a,q)=1 \end{array}} \sum _{\textbf{b}\in ({\mathbb {Z}}/q{\mathbb {Z}})^4}\sum _{\begin{array}{c} {\textbf{c}}\in ({{\mathbb {Z}}}/q{{\mathbb {Z}}})^2\\ \gcd (q,{\textbf{c}})=1 \end{array}} {\text {e}}_q\left( a\sum _{i=1}^{4}L_i({\textbf{c}})b_i^2\right) , \end{aligned}$$

for any $q\in {\mathbb {N}}$, where ${\text {e}}_q={\text {e}}(\frac{\cdot }{q})$. We may then define

2.11 $$\begin{aligned} \mathfrak {S}_2=\sum _{q=1}^\infty \frac{A_q}{q^6}\prod _{p\mid q}\left( 1-\frac{1}{p^{2}}\right) ^{-1}. \end{aligned}$$

The convergence of $\tau _\infty$ and $\mathfrak {S}_2$ are established in (7.24) and (7.26), respectively. We may now record the following result, which will be proved in Sect. 7.

### Proposition 2.3

Let $$\eta >0$$. Then

$$\begin{aligned} \#{{\mathscr {L}}}_2(B)=\frac{\tau _\infty \mathfrak {S}_2}{4\zeta (2)^2} B\log B +O(\eta ^{\frac{1}{2}}\log B) +O_\eta (1). \end{aligned}$$

Finally, for $\#{{\mathscr {L}}}_3(B)$ we shall produce the following upper bound.

### Proposition 2.4

Let $$\eta >0$$. Then

$$\begin{aligned} \#{{\mathscr {L}}}_3(B)= O\left( \eta B\log B +\frac{B\log B}{\log \log B}\right) . \end{aligned}$$

### Proof

Recalling (2.5), We have already remarked that $\#{{\mathscr {M}}}(X,Y)=0$ unless $X\ll Y^2$, which implies that $X\ll B^{\frac{1}{2}}$. Let ${\mathscr {X}}_1$ denote the set of $(X,Y)\in {\mathbb {N}}^2$, where *X*, *Y* run over non-negative powers of two such that $XY^{2}\ll B$ and $B^{\frac{1}{4}}\ll X\ll B^{\frac{1}{4}+\eta }.$ Similarly, let ${\mathscr {X}}_2$ denote the corresponding set in which the final inequalities are replaced by $B^{\frac{1}{2}-\eta }\ll X\ll B^{\frac{1}{2}}.$ Then it follows that

$$\begin{aligned} \#{{\mathscr {L}}}_3(B)\leqslant \sum _{\begin{array}{c} (X,Y)\in {\mathscr {X}}_1 \end{array}} \#{\mathscr {M}}(X,Y)+ \sum _{\begin{array}{c} (X,Y)\in {\mathscr {X}}_2 \end{array}} \#{\mathscr {M}}(X,Y). \end{aligned}$$

But Theorem 2.1 implies that

$$\begin{aligned} \sum _{\begin{array}{c} (X,Y)\in {\mathscr {X}}_1 \end{array}} \#{\mathscr {M}}(X,Y)&\ll \sum _{\begin{array}{c} (X,Y)\in {\mathscr {X}}_1 \end{array}} \left( XY^2 + X^{\frac{1}{4}}Y^{\frac{5}{2}} \frac{\log Y}{\log \log Y}\right) \\&\ll B\sum _{X}1 + \frac{B^{\frac{5}{4}}\log B}{\log \log B} \sum _X \frac{1}{X} \\&\ll \eta B\log B +\frac{B\log B}{\log \log B}, \end{aligned}$$

on summing over *X* and *Y*. Similarly, we obtain

$$\begin{aligned} \sum _{\begin{array}{c} (X,Y)\in {\mathscr {X}}_2 \end{array}} \#{\mathscr {M}}(X,Y)&\ll \eta B\log B+B. \end{aligned}$$

The statement of the lemma follows. $\square$

We shall combine these estimates in Sect. 8, which is where the proof of Theorem 1.1 will be drawn to a close.

### Notation

For any $D>0$, we shall write $d\sim D$ to mean $D\leqslant d<2D$, and we shall write $d\asymp D$ to mean that there exist constants $c_2>c_1>0$ (depending only on the linear forms $L_1,\dots ,L_4$ in (1.1)) such that $c_{1}D\leqslant d \leqslant c_{2}D$. Moreover, we shall often adopt the notation

$$\begin{aligned} X_1 \swarrow S\nearrow X_2 \end{aligned}$$

within a sum, in order to denote that a dyadic parameter *S* runs over an interval $X_1\ll S\ll X_2$, with implied constants depending only on the setting. We shall write $S\nearrow X$ to mean that the dyadic parameter *S* runs over an interval $S\ll X$.

## Preliminary technical results

### Character sum estimates

The following result is a straightforward consequence of the Burgess bound [7].

#### Lemma 3.1

(Burgess) Let $\chi$ be a non-principal Dirichlet character modulo *q*, let $\theta >\frac{3}{16}$ and let $\sigma \geqslant 1$. Then

$$\begin{aligned} \sum _{n>N}\frac{\chi (n)}{n^\sigma }\ll _\theta N^{\frac{1}{2}-\sigma }q^\theta . \end{aligned}$$

#### Proof

The Burgess bound, proved in [7], asserts that

$$\begin{aligned} \sum _{n\leqslant N}\chi (n)\ll _\theta N^\frac{1}{2}q^\theta , \end{aligned}$$

for any $\theta >\frac{3}{16}$. Using Abel’s summation formula, we obtain

$$\begin{aligned} \sum _{N<n\leqslant M}\frac{\chi (n)}{n^\sigma }&=\frac{1}{M^\sigma }\left( \sum _{N<n\leqslant M}\chi (n)\right) +\sum _{N<n\leqslant M}\left( \frac{1}{n^\sigma }-\frac{1}{(n+1)^\sigma }\right) \sum _{N<l\leqslant n}\chi (l), \end{aligned}$$

for every $M>N$. Applying the Burgess bound to the inner sum of the second term, we see that it is

$$\begin{aligned} \ll _\theta \sum _{N<n\leqslant M}\left( \frac{1}{n^\sigma }-\frac{1}{(n+1)^\sigma }\right) n^\frac{1}{2}q^\theta \ll N^{\frac{1}{2}-\sigma }q^\theta , \end{aligned}$$

uniformly for all $M>N$. On the other hand, the Burgess bound shows that the first term is $\ll _\theta M^{-\sigma +\frac{1}{2}}q^\theta <N^{-\sigma +\frac{1}{2}}q^\theta$, which completes the proof of the lemma. $\square$

We will also require a generalisation of the Pólya–Vinogradov bound that involves products of linear polynomials.

#### Lemma 3.2

Let $J_1,\dots ,J_{k}\in {\mathbb {Z}}[x_1,x_2]$ be pairwise non-proportional linear forms. Given $n\in {\mathbb {Z}}$ we write

$$\begin{aligned} J_{i,n}(x)=J_i(n,x) \in {\mathbb {Z}}[x], \end{aligned}$$

for $1\leqslant i\leqslant k$. Let ${\textbf{d}}=(d_{1},\dots ,d_{k})\in {{\mathbb {N}}}^{k}$ and put $D=d_1\cdots d_{k}$. Suppose that there exists $A\in {\mathbb {N}}$ such that $\gcd (d_i,d_j)\mid A$, for all $i\ne j$. Let $r\in {{\mathbb {N}}}$ be square-free, let $q\in {\mathbb {N}}$, let $a\in {\mathbb {Z}}/q{\mathbb {Z}}$ and let $I\subset {\mathbb {R}}$ be an interval. Assume that $\gcd (r,qD)=1$. Then for any $n\in {\mathbb {Z}}$ we have

$$\begin{aligned} \sum _{\begin{array}{c} x\in I\cap {\mathbb {Z}}\\ x\equiv a\bmod {q}\\ d_i\mid J_{i,n}(x) \end{array}} \left( \frac{J_{1,n}(x)\cdots J_{k,n}(x)}{r}\right) \ll _\varepsilon \left( \frac{{{\,\textrm{vol}\,}}(I)}{r^{\frac{1}{2}}[q, D]} + r^{\frac{1}{2}} \log (qD) \right) r^{\varepsilon }\gcd (rD,n), \end{aligned}$$

for any $\varepsilon >0$, where the implied constant is only allowed to depend on $A, J_1,\dots ,J_{k}$ and the choice of $\varepsilon$.

#### Proof

Let $$\Sigma _n(I)$$ denote the sum that we are trying to estimate. Suppose that $$ J_i(x_1,x_2)=a_ix_1+b_ix_2$$ for $$1\leqslant i\leqslant k$$, for $$a_i,b_i\in {\mathbb {Z}}$$. Put

$$\begin{aligned} \Delta _0= \prod _{1\leqslant i<j\leqslant k}|a_ib_j-a_jb_i|. \end{aligned}$$

Then $\Delta _0\ne 0$ since the linear forms are assumed to be non-proportional. Put

$$\begin{aligned} G(x)=J_{1,n}(x)\cdots J_{k,n}(x) \end{aligned}$$

and let $D=d_{1}\cdots d_{k}$. Recalling that $\gcd (r,qD)=1$, we may complete the sum by breaking into residue classes modulo *r*[*q*, *D*]. This yields

3.1 $$\begin{aligned} \begin{aligned} \Sigma _n(I)&=\sum _{\begin{array}{c} y\bmod r[q,D]\\ y\equiv a\bmod {q}\\ d_i\mid J_{i,n}(y) \end{array}} \left( \frac{G(y)}{r}\right) \sum _{\begin{array}{c} x\in I\cap {\mathbb {Z}} \end{array}} \frac{1}{r[q,D]}\sum _{\alpha \bmod r[q,D]} {\text {e}}_{r [q,D]}(\alpha (y-x))\\&= \frac{1}{r[q, D]}\sum _{\alpha \bmod r[q,D]}S(\alpha ) \sum _{\begin{array}{c} x\in I\cap {\mathbb {Z}} \end{array}}{\text {e}}_{r[q,D]}(-\alpha x), \end{aligned} \end{aligned}$$

where

$$\begin{aligned} S(\alpha )= \sum _{\begin{array}{c} y\bmod r[q,D]\\ y\equiv a\bmod {q}\\ d_i\mid J_{i,n}(z) \end{array}}\left( \frac{G(y)}{r}\right) {\text {e}}_{r[q,D]}(\alpha y). \end{aligned}$$

Any $\alpha \in {{\mathbb {Z}}}/r[q,D]{{\mathbb {Z}}}$ has a representative satisfying $|\alpha |\leqslant \frac{1}{2}r[q,D]$. Thus we clearly have

3.2 $$\begin{aligned} \left| \sum _{\begin{array}{c} x\in I\cap {\mathbb {Z}} \end{array}}{\text {e}}_{r[q,D]}(-\alpha x)\right| \ll {\left\{ \begin{array}{ll} {{\,\textrm{vol}\,}}(I)&{} \text { if } \alpha =0,\\ \frac{r[q,D]}{|\alpha |} &{}\text { if } \alpha \ne 0. \end{array}\right. } \end{aligned}$$

We now proceed with a detailed study of $S(\alpha )$. Since $\gcd (r,[q,D])=1$, any $y\bmod r[q,D]$ can be decomposed as $y= y_{1}[q,D]\overline{[q,D]} +y_{2}r\bar{r},$ for $y_{1}\bmod r$ and $y_{2}\bmod [q,D]$. (Here, $\overline{[q,D]}\in {\mathbb {Z}}$ is the multiplicative inverse of [*q*, *D*] modulo *r*, and $\overline{r}\in {\mathbb {Z}}$ is the inverse of *r* modulo [*q*, *D*].) Under this change of variables we obtain

$$\begin{aligned} S(\alpha )&= T(\alpha ;r) \sum _{\begin{array}{c} y_2\bmod [q,D]\\ y_2\equiv a \bmod {q}\\ d_i\mid J_{i,n}(y_2) \end{array}} {\text {e}}_{D}(\alpha y_{2}\overline{r}), \end{aligned}$$

where

$$\begin{aligned} T(\alpha ;r)= \sum _{y_{1}\bmod r}\left( \frac{G(y_1)}{r}\right) {\text {e}}_{r }(\alpha y_{1}\overline{[q,D]} ). \end{aligned}$$

But then it follows that

3.3 $$\begin{aligned} |S(\alpha )|\leqslant |T(\alpha ;r)| N(q;\textbf{d}), \end{aligned}$$

where

$$\begin{aligned} N(q;\textbf{d})= \#\left\{ y_2\bmod [q,D]: y_2\equiv a \bmod {q}, ~ d_i\mid J_{i,n}(y_2)\ \text {for}\ 1\leqslant i\leqslant k \right\} . \end{aligned}$$

It remains to estimate $T(\alpha ;r)$ and $N(q,\textbf{d})$.

We begin by estimating $T(\alpha ;r)$ for a square-free integer $r\in {\mathbb {N}}$. We shall prove that

3.4 $$\begin{aligned} T(\alpha ;r)\ll _\varepsilon r^{\frac{1}{2}+\varepsilon }\gcd (r,n)^{\frac{1}{2}}, \end{aligned}$$

for any $\varepsilon >0$, uniformly in $\alpha \in {\mathbb {Z}}/r{\mathbb {Z}}$. By multiplicativity, it will suffice to prove that

$$\begin{aligned} \sum _{y\bmod p}\left( \frac{G(y)}{p}\right) {\text {e}}_{p}(\alpha y)\ll {\left\{ \begin{array}{ll} \sqrt{p} &{} \text { if} p\not \mid n,\\ p &{} \text { if} p\mid n, \end{array}\right. } \end{aligned}$$

for any prime $p\mid r$, where the implied constant only depends on $\Delta _0$. The result is trivial if $p\mid \Delta _0$ and so we can assume that $p\not \mid \Delta _0$. But then it follows that the two linear polynomials $a_in+b_iT$ and $a_jn+b_jT$ are non-proportional modulo *p* if and only if $p\not \mid n$. Thus *G* is separable modulo *p* if and only if $p\not \mid n$. If $p\mid n$ we take the trivial bound for the exponential sum. If $p\not \mid n$, on the other hand, the desired bound follows from Weil’s resolution of the Riemann hypothesis for curves [28]. This completes the proof of (3.4).

Turning to $N(q;\textbf{d})$ we shall prove that

3.5 $$\begin{aligned} N(q;\textbf{d})=O(\gcd (D,n)), \end{aligned}$$

for an implied constant that is only allowed to depend on *A* and $J_1,\dots ,J_{k}$. Before doing so, let us see how it suffices to complete the proof of the lemma. Combining it with (3.4) in (3.3), we deduce that

$$\begin{aligned} S(\alpha )\ll _\varepsilon r^{\frac{1}{2}+\varepsilon }\gcd (r,n)^{\frac{1}{2}} \gcd (D,n)\leqslant r^{\frac{1}{2}+\varepsilon }\gcd (rD,n), \end{aligned}$$

since *r* and *D* are coprime. Once coupled with (3.2) in (3.1), we are finally led to the bound

$$\begin{aligned} \Sigma _n(I)&\ll _\varepsilon \frac{1}{r[q, D]} \left( {{\,\textrm{vol}\,}}(I)+ \sum _{0<|\alpha |\leqslant \frac{1}{2}r[q,D]} \frac{r[q,D]}{|\alpha |}\right) r^{\frac{1}{2}+\frac{\varepsilon }{2}}\gcd (rD,n)\\&\ll _\varepsilon \left( \frac{{{\,\textrm{vol}\,}}(I)}{r^{\frac{1}{2}}[q, D]} + r^{\frac{1}{2}} \log (qD) \right) r^{\varepsilon }\gcd (rD,n), \end{aligned}$$

as claimed in the lemma.

Returning to (3.5). It suffices to examine the case $N_p=N(p^\alpha ;p^{\beta _1},\dots ,p^{\beta _{k}})$, for any prime *p*, by the Chinese remainder theorem. We may suppose without loss of generality that $\alpha \geqslant 0$ and $0\leqslant \beta _1\leqslant \cdots \leqslant \beta _{k}$. It then follows from the hypotheses of the lemma that $p^{\beta _{k-1}}\mid A$. We now have

$$\begin{aligned} N_p \leqslant \#\left\{ y\bmod {p^{\max (\alpha ,\beta _1+\cdots +\beta _{k})}}: y\equiv a \bmod {p^\alpha }, ~ b_{k}y\equiv -a_{k}n\bmod {p^{\beta _{k}}} \right\} . \end{aligned}$$

If $\alpha \geqslant \beta _1+\cdots +\beta _{k}$, then we trivially have $N_p\leqslant 1$. If $\alpha < \beta _1+\cdots +\beta _{k}$, then

$$\begin{aligned} N_p \leqslant p^{\beta _1+\cdots +\beta _{k-1}} \#\left\{ y\bmod {p^{\beta _{k}}}: b_{k}y\equiv -a_{k}n\bmod {p^{\beta _{k}}} \right\} . \end{aligned}$$

Our remark above shows that $p^{\beta _1+\cdots +\beta _{k-1}}\leqslant p^{(k-1)v_p(A)}$. Moreover,

$$\begin{aligned} \#\left\{ y\bmod {p^{\beta _{k}}}: b_{k}y\equiv -a_{k}n\bmod {p^{\beta _{k}}} \right\} \leqslant \gcd (p^{\beta _{k}}, a_{k}n, b_{k}). \end{aligned}$$

If $b_{k}\ne 0$ this is at most $p^{v_p(b_{k})}$. On the other hand, if $b_{k}=0$, then this is at most $p^{v_p(a_k)}\gcd (p^{\beta _{k}},n)\leqslant p^{v_p(a_k)}\gcd (p^{\beta _1+\cdots +\beta _{k}},n)$. Taking a product over all primes, the bound in (3.5) easily follows. This completes the proof of the lemma. $\square$

### Pairs of quadrics modulo prime powers

Our first result concerns the roots of the pair of diagonal quadratic forms $Q_1,Q_2$ in (2.2). Recalling the definition (2.1) of $\Delta$, we have the following result.

#### Lemma 3.3

Let *p* be a prime and let $a\in {\mathbb {N}}$. Let

$$\begin{aligned} \nu _p={\left\{ \begin{array}{ll} 1 &{}\text { if}\,\, \, p=2,\\ 0 &{}\text { if}\,\, \, p>2. \end{array}\right. } \end{aligned}$$

Let $\textbf{u}\in \left( {\mathbb {Z}}/p^{a}{\mathbb {Z}}\right) ^{4}$ such that $p\not \mid \textbf{u}$ and $Q_{1}(\textbf{u})\equiv Q_{2}(\textbf{u})\equiv 0 \bmod {p^{a}}$. Then, for any integer $b>a$, we have

$$\begin{aligned} \#\left\{ \textbf{v}\in \left( {\mathbb {Z}}/p^{b}{\mathbb {Z}}\right) ^{4}:\begin{array}{l}Q_{1}(\textbf{v})\equiv Q_{2}(\textbf{v})\equiv 0 \bmod {p^{b}} \\ \textbf{v}\equiv \textbf{u}\bmod {p^{a}} \end{array}\right\} \leqslant {\left\{ \begin{array}{ll} p^{2(b-a)} &{}\text {if}\ p\not \mid \Delta ,\\ p^{2\nu _p+3(b-a)} &{}\text {if}\ p\mid \Delta . \end{array}\right. } \end{aligned}$$

#### Proof

Let us write $S_a(p^b)$ for the set whose cardinality is to be estimated. We begin by treating the case $p>2$, in which case $\nu _p=0$. We claim that

$$\begin{aligned} \#S_a(p^{b})\leqslant \#S_a(p^{b-1}) \times {\left\{ \begin{array}{ll} p^{2} &{}\text { if} p\not \mid \Delta ,\\ p^{3} &{}\text { if} p\mid \Delta , \end{array}\right. } \end{aligned}$$

for any $b> a$. In particular, this implies that $b\geqslant 2$, since $a\geqslant 1$. Noting that $\#S_a(p^a)=1$, an inductive argument completes the proof when $p>2$. To check the claim we note that any $\textbf{v}'\in S_a(p^b)$ can be written $\textbf{v}'=\textbf{v}+p^{b-1}\textbf{w}$ for $\textbf{v}\in S_a(p^{b-1})$ and $\textbf{w}\in ({\mathbb {Z}}/p{\mathbb {Z}})^4$. In particular $\textbf{v}'\equiv \textbf{u}\bmod {p^a}$. Moreover, the condition $p^b\mid Q_{i}(\textbf{v}')$ for $i=1,2$ implies that

$$\begin{aligned} p^{-b+1}Q_{i}(\textbf{v})+\nabla Q_{i}(\textbf{v})\cdot {\textbf{w}}\equiv 0\bmod {p}, \end{aligned}$$

for $i=1,2$. Note that $\nabla Q_1(\textbf{v})=2(a_1v_1,\dots , a_4v_4)$ and $\nabla Q_2(\textbf{v})=-2(b_1v_1,\dots , b_4v_4)$. Moreover, we have $\textbf{v}\equiv \textbf{u}\bmod {p}$, since $\textbf{v}\in S_a(p^{b-1})$. It follows that we are interested in counting ${\textbf{w}}\in ({\mathbb {Z}}/p{\mathbb {Z}})^4$ for which $p^{-b+1}Q_{i}(\textbf{v})+{\textbf{c}}_i\cdot {\textbf{w}}\equiv 0\bmod {p}$, where ${\textbf{c}}_i=\nabla Q_{i}(\textbf{u})$. If $p\not \mid \Delta$ then ${\textbf{c}}_1$ and ${\textbf{c}}_2$ are non-proportional modulo *p* and there are $p^{2}$ choices for ${\textbf{w}}$. If $p\mid \Delta$ but $p\not \mid {\textbf{c}}_1$ then we get at most $p^{3}$ choices for ${\textbf{w}}$. Similarly, if $p\mid \Delta$ but $p\not \mid {\textbf{c}}_2$. Finally, we note that $p\mid ({\textbf{c}}_1,{\textbf{c}}_2)$ is impossible, since it would then follow that $a_iu_i\equiv b_iu_i\equiv 0 \bmod {p}$ for $1\leqslant i\leqslant 4$, which contradicts the assumption $p\not \mid \textbf{u}$, since $\gcd (a_i,b_i)=1$ for $1\leqslant i\leqslant 4$.

It remains to deal with the case $p=2$. This time we note that any $\textbf{v}\in S_a(2^b)$ can be written $\textbf{v}=\textbf{u}+2^a {\textbf{w}}$, where $\textbf{w}\in ({\mathbb {Z}}/2^{b-a}{\mathbb {Z}})^4$. If $2^b\mid Q_1(\textbf{v})$ then it easily follows $2^{a+1}\mid Q_1(\textbf{u})$, whence

$$\begin{aligned} A+\sum _{i=1}^4 a_iu_iw_i +2^{a-1}Q_1({\textbf{w}}) \equiv 0\bmod {2^{b-a-1}}, \end{aligned}$$

where $$A=Q_1(\textbf{u})/2^{a+1}\in {\mathbb {Z}}$$. Similarly,

$$\begin{aligned} B+\sum _{i=1}^4 b_iu_iw_i +2^{a-1}Q_2({\textbf{w}}) \equiv 0\bmod {2^{b-a-1}}, \end{aligned}$$

where $$B=Q_2(\textbf{u})/2^{a+1}\in {\mathbb {Z}}$$. Since $$2\not \mid \textbf{u}$$, we may assume without loss of generality that $$2\not \mid u_1$$. Moreover, since $$\gcd (a_1,b_1)=1$$, we may further assume that $$2\not \mid a_1$$. Hence, for $$2^{3(b-a)}$$ choices of $$w_2,w_3,w_4$$, we are left with counting the number of $$w_1 \in {\mathbb {Z}}/2^{b-a}{\mathbb {Z}}$$ such that $$f(w_1)\equiv 0 \bmod {2^{b-a-1}}$$, where $$ f(x)=2^{a-1}a_1x^2+a_1u_1x+C, $$ for an appropriate integer *C*. Since $$f'(x)=2^a a_1x+a_1u_1$$ is always odd, so it follows from Hensel’s lemma that the congruence $$f(x)\equiv 0 \bmod {2^{b-a-1}}$$ has at most 2 roots modulo $$2^{b-a-1}$$, for fixed $$w_2,w_3,w_4$$. This therefore implies that $$ \#S_a(2^b)\leqslant 2^{2+3(b-a)}, $$ which completes the proof of the lemma. $$\square $$

Of special interest in our work will be the functions

3.6 $$\begin{aligned} \varrho (q)=\#\{\textbf{y}\in ({\mathbb {Z}}/q{\mathbb {Z}})^4:Q_1(\textbf{y})\equiv Q_2(\textbf{y})\equiv 0\bmod q\} \end{aligned}$$

and

3.7 $$\begin{aligned} \varrho ^*(q)=\#\{\textbf{y}\in ({\mathbb {Z}}/q{\mathbb {Z}})^4: \gcd (\textbf{y},q)=1,~ Q_1(\textbf{y})\equiv Q_2(\textbf{y})\equiv 0\bmod q\}, \end{aligned}$$

for any $q\in {\mathbb {N}}$. These counting functions have already featured in work of Browning and Munshi [6], leading to the following result.

#### Lemma 3.4

Let *p* be a prime and let $r\in {\mathbb {N}}$. Then we have

(i) $$\varrho ^*(p^r)= p^{2r}(1+O(p^{-\frac{1}{2}}))$$ if $$p\not \mid \Delta $$.
(ii) $$\varrho (p^r)=O(rp^{2r})$$.

#### Proof

If $p\not \mid \Delta$, then the curve $Q_{1}=Q_{2}=0$ defines a smooth curve over ${\mathbb {F}}_{p}$ and (i) follows from combining Lemma  3.3 with the Weil bound. Alternatively, for any *p*, (ii) follows from the proof of [6, Lemma 2]. $\square$

### Geometry of numbers and a special lattice

We shall also care deeply about the shape of a certain lattice that features in our work. For any $d\in {\mathbb {N}}$, we can define an equivalence relation on $\left( {\mathbb {Z}}/d{\mathbb {Z}}\right) ^{4}$, by saying $\textbf{u}$ is equivalent to $\textbf{v}$ if and only if there exists $\lambda \in \left( {\mathbb {Z}}/d{\mathbb {Z}}\right) ^{\times }$ such that $\lambda \textbf{u}\equiv \textbf{v}\bmod {d}$. We shall be interested in the set of equivalence classes

3.8 $$\begin{aligned} V_{d}^{\times }=\{\textbf{u}\in \left( {\mathbb {Z}}/d{\mathbb {Z}}\right) ^{4}: \gcd (\textbf{u},d)=1,~Q_{1}(\textbf{u})\equiv Q_{2}(\textbf{u})\equiv 0 \bmod {d}\}/\left( {\mathbb {Z}}/d{\mathbb {Z}}\right) ^{\times }.\nonumber \\ \end{aligned}$$

For any $\textbf{u}\in \left( {\mathbb {Z}}/d{\mathbb {Z}}\right) ^{4}$ such that $\gcd (\textbf{u},d)=1$ and $Q_{1}(\textbf{u})\equiv Q_{2}(\textbf{u})\equiv 0 \bmod {d}$, we will denote by $[\textbf{u}]$ its class in $V_{d}^{\times }$. For any $[\textbf{u}]\in V_{d}^{\times }$, and any $k\mid d$, the main goal of this section is to discuss various properties of the lattice

3.9 $$\begin{aligned} \Lambda _{[\textbf{u}],k}=\left\{ \textbf{y}\in {\mathbb {Z}}^{4}:\text {} \exists \lambda \in {\mathbb {Z}}\text { such that } \textbf{y}\equiv \lambda \textbf{u}\bmod {k}\right\} . \end{aligned}$$

This definition is clearly independent of the particular choice of representative $\textbf{u}\in [\textbf{u}]$. The lattice $\Lambda _{[\textbf{u}],k}$ has rank 4 and determinant $k^{3}$. We denote by $1\leqslant s_{1,[\textbf{u}],k}\leqslant \cdots \leqslant s_{4,[\textbf{u}],k}$ the associated successive minima. It follows from Minkowski’s second theorem that

3.10 $$\begin{aligned} k^3\ll s_{1,[\textbf{u}],k} \cdots s_{4,[\textbf{u}],k}\ll k^3. \end{aligned}$$

We will need good estimates for the size of $V_d^\times$ and also for the number of classes in $V_d^\times$ which reduce modulo *k* to a given class in $V_k^\times$, for any $k\mid d$. First we introduce some notation. For any $n\in {\mathbb {N}}$, we write

3.11 $$\begin{aligned} n_{\Delta }=\prod _{p\mid \Delta }p^{v_{p}(n)}. \end{aligned}$$

With this in mind, we shall prove the following result.

#### Lemma 3.5

Let $d,k\in {\mathbb {N}}$ such that $k\mid d$. Let $[\textbf{u}]\in V_k^\times$. Then

$$\begin{aligned} \#\{[\textbf{v}]\in V_{d}^{\times }:[\textbf{v}\bmod {k}]=[\textbf{u}]\}\ll \left( \frac{d}{k}\right) _{\Delta }\cdot \frac{d}{k}. \end{aligned}$$

Moreover, we have

$$\begin{aligned} \#V_{d}^{\times }\ll _{\varepsilon }d \cdot d_{\Delta }^{\varepsilon } \cdot \prod _{\begin{array}{c} p\mid d\\ p\not \mid \Delta \end{array}}(1+O(p^{-\frac{1}{2}})). \end{aligned}$$

#### Proof

By the Chinese remainder theorem it will suffice to treat the case that $k=p^a$ and $d=p^b$ for a prime *p* and integers $0\leqslant a<b$. For any $[\textbf{u}]\in V_{p^a}^\times$, we observe that

3.12 $$\begin{aligned} \begin{aligned} \#&\left\{ \textbf{v}\in \left( {\mathbb {Z}}/p^{b}{\mathbb {Z}}\right) ^{4}:\begin{array}{l}Q_{1}(\textbf{v})\equiv Q_{2}(\textbf{v})\equiv 0\bmod {p^b}\\ \textbf{v}\bmod {p^{a}}\in [\textbf{u}] \end{array}\right\} \\&\qquad \qquad = \varphi (p^{b})\#\left\{ [\textbf{v}]\in V_{p^{b}}^{\times }: [\textbf{v}\bmod {p^{a}}]=[\textbf{u}]\right\} . \end{aligned} \end{aligned}$$

Taking $a=0$, we deduce that

3.13 $$\begin{aligned} \#V_{p^b}^\times =\frac{\varrho ^*(p^b)}{p^{b}}\left( 1-\frac{1}{p}\right) ^{-1}, \end{aligned}$$

for any $b\in {\mathbb {N}}$, where $\varrho ^*(p^b)$ is defined in (3.7). The second part of the lemma is now a consequence of Lemma 3.4.

To handle the first part of the lemma, we may clearly assume that $a\geqslant 1$. We observe that the left hand side of (3.12) is

$$\begin{aligned} \sum _{\textbf{u}'\in [\textbf{u}]}\#\left\{ \textbf{v}\in \left( {\mathbb {Z}}/p^{b}{\mathbb {Z}}\right) ^{4}:\begin{array}{l}Q_{1}(\textbf{v})\equiv Q_{2}(\textbf{v})\equiv 0\bmod {p^{b}}\\ \textbf{v}\equiv \textbf{u}' \bmod {p^{a}} \end{array}\right\} . \end{aligned}$$

The number of $\textbf{u}'$ in the outer sum is $\varphi (p^a)$. Moreover, we can use Lemma 3.3 to estimate the remaining cardinality, which easily completes the proof of the lemma, since $\varphi (p^b)=\varphi (p^a)p^{b-a}$ if $a\geqslant 1$. $\square$

We take this opportunity to record the following facts about $\#V_{p^b}^\times$ for generic primes.

#### Corollary 3.6

For any prime $p\not \mid \Delta$ and $b\in {\mathbb {N}}$, we have $\#V_{p^b}^\times =p^b(1+O(p^{-\frac{1}{2}}))$.

#### Proof

This follows on combining (3.13) with Lemma 3.4 in the case $p\not \mid \Delta$. $\square$

## Counting points on quadric surfaces

For given non-zero $A_1,\dots ,A_4\in {\mathbb {Z}}$, let $Q(\textbf{y})=A_1y_1^2+A_2y_2^2+A_3y_3^2+A_4y_4^2$ be a fixed diagonal quadratic form. In this section we record some estimates for the counting function

4.1 $$\begin{aligned} N(Q;B)=\#\{\textbf{y}\in {{\mathbb {Z}}}_{{\text {prim}}}^4:Q(\textbf{y})=0,|\textbf{y}|\leqslant B\}, \end{aligned}$$

which have the key feature that they depend uniformly on $A_1,\dots ,A_4$. Our first estimate is based on the geometry of numbers arguments used in [4], and our second is based on a circle method analysis [3].

Let $\varDelta _Q=A_1A_2A_3A_4$ be the discriminant of *Q* and let $\Vert Q\Vert =\max _{1\leqslant i\leqslant 4}|A_i|$ be its height. We define a Dirichlet character $\chi _Q$ induced by the Kronecker symbol $(\frac{\varDelta _Q}{\cdot })$. Let $\varpi$ be the multiplicative arithmetic function defined by

4.2 $$\begin{aligned} \varpi (m)=\prod _{p\mid m}\left( 1+\frac{1}{p}\right) \end{aligned}$$

and set

4.3 $$\begin{aligned} \varDelta _{{\text {bad}}}=\prod _{\begin{array}{c} p^e\Vert \varDelta _Q\\ e\geqslant 2 \end{array}}p^e. \end{aligned}$$

Combining the argument in [4, p.3] with the main result of [4], we obtain the following bound

### Lemma 4.1

Let $\varepsilon >0$. If $\varDelta _{{\text {bad}}}\leqslant B^\frac{1}{20}$, then

$$\begin{aligned} N(Q;B)\ll _{\varepsilon } \varpi (\varDelta _Q)\varDelta _{{\text {bad}}}^{\frac{1}{4}+\varepsilon }\left( \frac{\Vert Q\Vert ^4}{|\varDelta _Q|}\right) ^\frac{5}{8} \left( B^\frac{4}{3}+\frac{B^2}{|\varDelta _Q|^\frac{1}{4}}\right) L(\sigma _B,\chi _Q), \end{aligned}$$

where $$\sigma _B=1+\frac{1}{\log B}$$.

The appearance of the factor $\varDelta _{{\text {bad}}}^{\frac{1}{4}+\varepsilon }$ will be problematic when $\varDelta _{{\text {bad}}}$ is large. We now revisit the arguments in [4] to show that the dependence on $\varDelta _{{\text {bad}}}$ can be mitigated at the expense of an additional $B^\varepsilon$-factor. This is summarised in the following result.

### Lemma 4.2

Let $$\varepsilon >0$$. Then

$$\begin{aligned} N(Q;B)\ll _\varepsilon B+B^\varepsilon \sum _{\begin{array}{c} {\textbf{c}}\in {{\mathbb {Z}}}_{{\text {prim}}}^4\\ |{\textbf{c}}|\ll B^\frac{1}{3}\\ Q^*({\textbf{c}})\ne 0 \end{array}}\left( 1+B\frac{\Vert Q\Vert }{|\varDelta _Q|^\frac{1}{2}}\frac{\gcd (\varDelta _{{\text {bad}}}^3,Q^*({\textbf{c}})^2)^\frac{1}{6}}{|{\textbf{c}}|}\right) , \end{aligned}$$

where $Q^*$ is the dual quadratic form.

### Proof

We sketch the proof of Lemma 4.1. The main idea is to use Siegel’s lemma to cover with plane sections the integer solutions to the equation $Q(\textbf{y})=0$ which lie in the box $|\textbf{y}|\leqslant B$. Thus any such point lies on at least one plane ${\textbf{c}}\cdot \textbf{y}=0$, where ${\textbf{c}}\in {{\mathbb {Z}}}_{{\text {prim}}}^4$ satisfies $|{\textbf{c}}|\ll B^\frac{1}{3}$, for an absolute implied constant. This produces a union of conics $Q_{\textbf{c}}$, as in [4, Lemma 2.1]. We cover points on each conic $Q_{\textbf{c}}$ using a family of ellipsoids, the number of which is effectively bounded in terms of the dual form $Q^*$ and ${\textbf{c}}$. This is the object of [4, Lemma 2.2]. In this way, the problem reduces to counting lattice points in a conic within a fixed ellipsoid, which can be transformed to counting points on conics in unequal boxes.

For the purposes of the lemma the main idea is to not use the inequality displayed above [4, Eq. (2.16)], but to take the trivial bounds $\log (2+|{\textbf{c}}|^2\Vert Q\Vert ^3/|Q^*({\textbf{c}})|)=O_\varepsilon (B^\varepsilon )$ and $R(Q^*({\textbf{c}}))=O_\varepsilon (B^\varepsilon )$ at the close of [4, § 2.2]. The statement of the lemma easily follows. $\square$

Our next estimate for *N*(*Q*; *B*) in (4.1) is based on the circle method. Let us begin with a few remarks about the singular series $\mathfrak {S}(Q)$. This is defined to be

4.4 $$\begin{aligned} \mathfrak {S}(Q)=\prod _p\sigma _p, \end{aligned}$$

where

$$\begin{aligned} \sigma _p=\lim _{k\rightarrow \infty }\frac{\#\left\{ \textbf{x}\in ({\mathbb {Z}}/p^k{\mathbb {Z}})^4: Q(\textbf{x})\equiv 0 \bmod {p^k} \right\} }{p^{3k}}. \end{aligned}$$

The following result is concerned with an upper bound for $\mathfrak {S}(Q)$.

### Lemma 4.3

Let $\varepsilon >0$. Assume that there exists $A\in {\mathbb {N}}$ such that $\gcd (A_i,A_j)\mid A$, for all distinct $i,j\in \{1,\dots , 4\}$. Then

$$\begin{aligned} \mathfrak {S}(Q)\ll _{\varepsilon ,A} \varDelta _{{\text {bad}}}^\varepsilon L(1,\chi _Q), \end{aligned}$$

where $\varDelta _{{\text {bad}}}$ is given by (4.3) and the implied constant depends on $\varepsilon$ and *A*.

### Proof

On revisiting the proof of [5, Lemma 4.10], it is shown that $\prod _{p\not \mid 2\varDelta }\sigma _p\ll L(1,\chi _{Q}).$ Moreover, by [5, Lemma 4.8], we have $\sigma _p=1$ if $p\mid \varDelta _Q$ but $p\not \mid 2\varDelta _{{\text {bad}}}$. Thus

$$\begin{aligned} \mathfrak {S}(Q)\ll L(1,\chi _Q) \prod _{p\mid 2\varDelta _{{\text {bad}}}} \sigma _p. \end{aligned}$$

To estimate the remaining product, we examine $\sigma _p$ for a given prime *p*. Let $f_{i}=v_p(A_i)$, for $1\leqslant i\leqslant 4$, and assume without loss of generality that $f_{1}\leqslant f_2\leqslant \cdots \leqslant f_{4}$. Then the last part of the proof of [5, Lemma 4.10] gives $\sigma _p\ll p^{(f_1+f_2+f_3)/3}.$ It follows that $\sigma _p=O_A(1)$ for an implied constant that is allowed to depend on *A*. We obtain the statement of the lemma on taking the product over all $p\mid 2\varDelta _{{\text {bad}}}$. $\square$

The next result has the advantage that there is no restriction on the size of $\varDelta _{{\text {bad}}}$, or on the size of coefficients, but it comes at the expense of a worse error term.

### Lemma 4.4

Let $\varepsilon >0$ and let $m(Q)=\min _{1\leqslant i\leqslant 4} |A_i|$. Assume that there exists $A\in {\mathbb {N}}$ such that $\gcd (A_i,A_j)\mid A$, for all distinct $i,j\in \{1,\dots , 4\}$. Then

$$\begin{aligned} N(Q;B)\ll _{\varepsilon ,A} \frac{ \varDelta _{{\text {bad}}}^\varepsilon L(1,\chi _Q) }{(m(Q)\Vert Q\Vert )^\frac{1}{2}}B^2+\frac{\Vert Q\Vert ^{11+\varepsilon }}{m(Q)^6|\varDelta _Q|^\frac{1}{2}}B^{\frac{3}{2}+\varepsilon }. \end{aligned}$$

### Proof

In fact we shall prove the same upper bound for the quantity $N'(Q;B)$, in which the stipulation that $\textbf{y}$ is primitive is dropped. To do so, we shall actually apply an asymptotic formula for a smoothly weighted version of this counting function. Consider the non-negative smooth weight function

$$\begin{aligned} w(x)={\left\{ \begin{array}{ll} \exp \left( -(1-x^2)^{-1}\right) &{} \text { if } |x|<1\\ 0 &{} \text { if } |x|\geqslant 1, \end{array}\right. } \end{aligned}$$

and define

$$\begin{aligned} \omega (x)= \left( \int _{-\infty }^{\infty }w(x){\text {d}}x\right) ^{-1}3 \int _{x-\frac{2}{3}}^{x-\frac{1}{3}}w(3y){\text {d}}y. \end{aligned}$$

This is a smooth function that takes values in [0, 1] and is supported on (0, 1). Now for $\textbf{x}\in {{\mathbb {R}}}^4$, we put

$$\begin{aligned} w_1(\textbf{x})=w(x_1-2)\prod _{i=2}^4\omega \left( 1-\frac{x_i}{x_1}\right) . \end{aligned}$$

Then $w_1$ is supported on the set $\{\textbf{x}\in {{\mathbb {R}}}^4:1\leqslant x_1\leqslant 3,~ 0\leqslant x_2,x_3,x_4\leqslant x_1\}.$ We define the weighted counting function

$$\begin{aligned} N^\prime _{w_1}(Q;B)= \sum _{\begin{array}{c} \textbf{y}\in {{\mathbb {Z}}}^4\\ Q(\textbf{y})=0 \end{array}}w_1\left( \frac{\textbf{y}}{B}\right) . \end{aligned}$$

Under the assumption that $\varDelta _Q\ne \square$, it follows from [3, Prop. 2] that

$$\begin{aligned} N^\prime _{w_1}(Q;B)=\sigma _{\infty ,w_1}(Q)\mathfrak {S}(Q)B^2+O_\varepsilon \left( \frac{\Vert Q\Vert ^{11+\varepsilon }}{|A_1|^6|\varDelta _Q|^\frac{1}{2}}B^{\frac{3}{2}+\varepsilon }\right) , \end{aligned}$$

for any $\varepsilon >0$. Here, $\mathfrak {S}(Q)$ is given by (4.4) and $\sigma _{\infty ,w_1}(Q)\geqslant 0$ is the singular integral, which is shown to satisfy $\sigma _{\infty ,w_1}(Q)\ll (|A_1|\Vert Q\Vert )^{-\frac{1}{2}}$ in [3, Eq. (2.6)].

Finally, to obtain a uniform bound for the counting function $N^\prime (Q;B)$, we follow the argument in [3, p. 18]. Let $Q^\sigma$ denote the quadratic forms obtained by permuting the coefficients of *Q*, for any $\sigma \in S_4$. On decomposing the interval $[-B,B]$ into dyadic intervals, we have

$$\begin{aligned} N^\prime (Q;B)&\ll 1+\sum _{\sigma \in S_4}\sum _{j=0}^{\infty }N^\prime _{w_1}(Q^\sigma ; 2^{-j}B) \ll _\varepsilon \frac{\mathfrak {S}(Q)B^2}{(m(Q)\Vert Q\Vert )^\frac{1}{2}} +\frac{\Vert Q\Vert ^{11+\varepsilon }}{m(Q)^6|\varDelta _Q|^\frac{1}{2}}B^{\frac{3}{2}+\varepsilon }. \end{aligned}$$

An application of Lemma 4.3 now completes the proof. $\square$

## Upper bounds for $\#{{\mathscr {M}}}(X,Y)$ and related quantities

Let $X,Y\geqslant 1$. The main goal of this section is to prove Theorem  2.1, which is concerned with estimating $\#{{\mathscr {M}}}(X,Y)$, where ${{\mathscr {M}}}(X,Y)$ is defined in (2.5). Along the way we shall establish several auxiliary estimates that will have their own role to play. This section should be seen as an analogy to [5, § 2], the principal results of which are [5, Lemmas 2.1 and 2.7]. However, unlike the variety studied in [5], we have less symmetry and fewer variables. This prohibits the ability to apply the arguments based on Hua’s inequality that were used to great effect in [5]. Rather, our proof of Theorem 2.1 relies on a number of different upper bounds that will be played off against each other and which we proceed to record here.

We begin by dealing with the counting problem in which the condition (2.3) fails. Thus let

$$\begin{aligned} {\mathscr {M}}^\square (X,Y)=\left\{ (\textbf{x},\textbf{y})\in {\mathbb {Z}}_{\text {prim}}^{2}\times {\mathbb {Z}}^{4}: \begin{array}{l} (1.1)\ \text {and}\ (1.4)\ \text {hold}\\ |\textbf{x}|\leqslant X, ~|\textbf{y}|\leqslant Y \end{array}\right\} , \end{aligned}$$

for $X,Y\geqslant 1$. We have the following estimate.

### Lemma 5.1

Let $$\varepsilon >0$$. Then $$\#{\mathscr {M}}^\square (X,Y)\ll _\varepsilon X^\varepsilon Y^{2+\varepsilon }$$.

### Proof

For a fixed choice of $\textbf{x}$, the quadric in (1.1) admits $O_\varepsilon (Y^{2+\varepsilon })$ solutions $\textbf{y}\in {\mathbb {Z}}^4$ with $|\textbf{y}|\leqslant Y$, thanks to work of Heath-Brown [13, Thm. 2]. It is important to emphasise here that the implied constant is only allowed to depend on $\varepsilon >0$, and not on $\textbf{x}$. It remains to estimate the number of zeros $(z,\textbf{x})\in {\mathbb {Z}}^3$ of the equation $z^{2}=L_{1}(\textbf{x})\cdots L_{4}(\textbf{x}),$ with $\gcd (x_1,x_2)=1$ and $|\textbf{x}|\leqslant X$. This equation defines a genus 1 curve in weighted projective space ${\mathbb {P}}(2,1,1)$ and so it has $O_\varepsilon (X^\varepsilon )$ solutions by the theory of Néron heights. The statement of the lemma follows. $\square$

We now return to the task of estimating $\#{{\mathscr {M}}}(X,Y)$. For any $\textbf{x}\in {{\mathbb {Z}}}_{{\text {prim}}}^2$ such that $L_1(\textbf{x})\cdots L_4(\textbf{x})\ne 0$, we define

5.1 $$\begin{aligned} \varDelta _{{\text {bad}}}(\textbf{x})=\prod _{\begin{array}{c} p^e\Vert L_1(\textbf{x})\cdots L_4(\textbf{x})\\ e\geqslant 2 \end{array}}p^e. \end{aligned}$$

For given $D\geqslant 1$, we let

5.2 $$\begin{aligned} {{\mathscr {M}}}_1(X,Y;D)=\{(\textbf{x},\textbf{y})\in {{\mathscr {M}}}(X,Y):\varDelta _{{\text {bad}}}(\textbf{x})\leqslant D\} \end{aligned}$$

and

5.3 $$\begin{aligned} {{\mathscr {M}}}_2(X,Y;D)=\{(\textbf{x},\textbf{y})\in {{\mathscr {M}}}(X,Y):\varDelta _{{\text {bad}}}(\textbf{x})> D\}. \end{aligned}$$

We shall prove the following upper bound for the size of the first set.

### Proposition 5.2

Let $\varepsilon >0$ and assume that $D\leqslant Y^{\frac{1}{20}}$. Then

$$\begin{aligned} \#{{\mathscr {M}}}_1(X,Y;D)\ll _{\varepsilon } XY^2+X^2Y^\frac{4}{3} +(D X)^\varepsilon \left( D^{\frac{3}{4}}(X^\frac{15}{8}Y^\frac{4}{3}+X^\frac{7}{8}Y^2)\right) . \end{aligned}$$

The main tool in the proof of this result is [4, Thm. 1.1], which is recorded in Lemma 4.1 and which requires $\varDelta _{{\text {bad}}}(\textbf{x})$ to be sufficiently small. It is worth taking a moment to compare with the analogous situation in [5]. There, a version of Proposition 5.2 is proved using [4, Thm. 1.1], in which there is no appearance of any power of *D*. In our situation, the factor $\varDelta _{{\text {bad}}}(\textbf{x})^{1/4+\varepsilon }$ in [4, Thm. 1.1] becomes a major technical issue. At the expense of allowing an additional $(XY)^\varepsilon$-factor, we will show that the argument behind Proposition 5.2 can be adjusted to prove the following result.

### Proposition 5.3

Let $$\varepsilon >0$$. Then

$$\begin{aligned} \#{{\mathscr {M}}}_2(X,Y;D)\ll _\varepsilon (XY)^\varepsilon \left( \frac{XY^2+X^\frac{5}{2}Y}{D^\frac{1}{16}}+X^\frac{3}{2}Y+X^\frac{1}{2}Y^{\frac{4}{3}}\right) . \end{aligned}$$

Unfortunately, Propositions 5.2 and 5.3 are not quite enough to provide a satisfactory estimate for $\#{{\mathscr {M}}}(X,Y)$ when *X* is a very small power of *Y*. However, in this particular case, we can invoke the following upper bound.

### Proposition 5.4

Let $$\varepsilon >0$$. Then

$$\begin{aligned} \#{{\mathscr {M}}}(X,Y)\ll _\varepsilon XY^2+X^{11} Y^{\frac{3}{2}+\varepsilon }. \end{aligned}$$

This result can be viewed as a weak version of Theorem 2.1 and is based on Lemma 4.4. While the principal terms $XY^2$ agree, Proposition 5.4 is much worse when *X* is large. Later in our argument it will be useful to have a good upper bound for the quantity

5.4 $$\begin{aligned} {\mathscr {M}}^*(X,Y)=\left\{ (\textbf{x},\textbf{y})\in {{\mathbb {Z}}}_{{\text {prim}}}^2\times {\mathbb {Z}}^4: (1.1)\ \text {holds}, ~ |\textbf{x}|\leqslant X,~|\textbf{y}|\leqslant Y\right\} , \end{aligned}$$

for any $X,Y\geqslant 1$. Note that $\#{{\mathscr {M}}}(X,Y)\leqslant \#{{\mathscr {M}}}^*(2X,Y)$, since we have merely dropped from ${{\mathscr {M}}}(X,Y)$ the constraint that $\textbf{y}$ be primitive, as well as the condition (2.3). We can combine Propositions  5.2–5.4 to deduce the following result.

### Corollary 5.5

Let $\varepsilon >0$ and let $X,Y\geqslant 1$ such that $X\leqslant Y^{2/3}\log Y$. Then

$$\begin{aligned} \#{{\mathscr {M}}}^*(X,Y)\ll _\varepsilon Y^{2+\varepsilon }+ XY^2+X^2Y^{4/3}. \end{aligned}$$

### Proof

We would like to insert the condition (2.3) into ${\mathscr {M}}^*(X,Y)$. But the overall contribution from those $(\textbf{x},\textbf{y})$ for which (2.3) fails is $O_\varepsilon (Y^{2+\varepsilon })$, thanks to Lemma 5.1 and the fact that $X\leqslant Y^{2/3}\log Y\ll Y$. Sorting the remaining contribution according to the greatest common divisor of the coordinates of $\textbf{y}$, and breaking the $\textbf{x}$-sum into dyadic intervals, it easily follows that

5.5 $$\begin{aligned} \#{{\mathscr {M}}}^*(X,Y)\ll _\varepsilon Y^{2+\varepsilon } +\sum _{d\leqslant Y}\sum _{X_0 \nearrow X}\#{{\mathscr {M}}}(X_0,Y/d), \end{aligned}$$

for any $\varepsilon >0$. We claim that there exists $\varepsilon >0$ such that

5.6 $$\begin{aligned} \#{{\mathscr {M}}}(X,Y/d)\ll _\varepsilon \frac{XY^2+X^2Y^{4/3}}{d^{1+\varepsilon }}, \end{aligned}$$

if $X\leqslant Y^{2/3}\log Y$. Once inserted into (5.5), this will clearly suffice to complete the proof of the corollary.

Now if $X\leqslant Y^{1/100}$, it is clear that (5.6) follows from Proposition 5.4. Thus we may proceed under the assumption that $Y^{1/100}\leqslant X$. Under the further assumption $X\leqslant Y^{2/3}\log Y$, we proceed by inspecting some of the terms in Proposition 5.3. Note that

$$\begin{aligned} (XY/d)^\varepsilon X^\frac{3}{2}\left( \frac{Y}{d}\right) \ll _\varepsilon \frac{XY^{4/3+2\varepsilon }}{d^{1+\varepsilon }}\ll \frac{XY^2}{ d^{1+\varepsilon }}, \end{aligned}$$

on assuming that $\varepsilon \leqslant 1/3$. Moreover, $(XY/d)^\varepsilon X^\frac{1}{2}(Y/d)^{\frac{4}{3}}\leqslant d^{-\frac{4}{3}}XY^2$ and

$$\begin{aligned} (XY/d)^\varepsilon X^{\frac{5}{2}}\left( \frac{Y}{d}\right) \ll _\varepsilon \frac{XY^{2+2\varepsilon }}{d^{1+\varepsilon }}. \end{aligned}$$

Turning to the terms in Proposition 5.2, we have

$$\begin{aligned} (D X)^\varepsilon \left( D^{\frac{3}{4}}(X^\frac{15}{8}(Y/d)^\frac{4}{3}+X^\frac{7}{8}(Y/d)^2)\right)&\ll \frac{D^{\frac{3}{4}+\varepsilon }}{d^{\frac{4}{3}}} \left( X (Y^{2/3})^{\frac{7}{8}+\varepsilon } Y^{\frac{4}{3}} +X^{\frac{7}{8}+\varepsilon }Y^2\right) \\&\ll \frac{XY^2 D^{\frac{3}{4}+\varepsilon } }{d^{\frac{4}{3}}}\left( \frac{1}{Y^{\frac{1}{12}-\varepsilon }} + \frac{1}{X^{\frac{1}{8}-\varepsilon }}\right) \\&\ll \frac{XY^2}{d^{\frac{4}{3}}} \frac{D^{\frac{3}{4}+\varepsilon } }{X^{\frac{1}{8}-2\varepsilon }}, \end{aligned}$$

if $$X\leqslant Y^{2/3}\log Y$$.

Hence, on combining Propositions 5.2 and 5.3, we deduce that

$$\begin{aligned} \#{{\mathscr {M}}}(X,Y/d)\ll _\varepsilon \frac{XY^2+X^2Y^{\frac{4}{3}}}{d^{1+\varepsilon }}\left( 1+ \min \left( \frac{D^{\frac{3}{4}+\varepsilon } }{X^{\frac{1}{8}-2\varepsilon }}, \frac{Y^{2\varepsilon }}{D^{\frac{1}{16}}} \right) \right) , \end{aligned}$$

for any $D\leqslant Y^{1/20}$ and $Y^{1/100}<X\leqslant Y^{2/3}\log Y$. But then

$$\begin{aligned} \min \left( \frac{D^{\frac{3}{4}+\varepsilon } }{X^{\frac{1}{8}-2\varepsilon }}, \frac{Y^{2\varepsilon }}{D^{\frac{1}{16}}} \right)&\leqslant \min \left( \frac{D^{\frac{3}{4}} }{Y^{\frac{1}{800}}}, \frac{1}{D^{\frac{1}{16}}} \right) Y^{2\varepsilon }. \end{aligned}$$

We can ensure that this is *O*(1) by taking $D=Y^{1/800}$ and choosing a sufficiently small value of $\varepsilon$. This completes the proof of (5.6). $\square$

The results so far are efficient when *X* is small compared to *Y*. The following result is proved using completely different methods and allows us to handle the opposite case.

### Proposition 5.6

We have

$$\begin{aligned} \#{{\mathscr {M}}}(X,Y) \ll XY^{2}+\left( \frac{Y^{4}}{X^2} +X^{\frac{1}{4}}Y^{\frac{5}{2}}\right) \frac{\log Y}{\log \log Y}. \end{aligned}$$

Once in possession of Propositions  5.2–5.6, we are now positioned to prove our main upper bound for $\#{{\mathscr {M}}}(X,Y)$.

### Proof of Theorem 2.1

Let us put $J=(\log Y)/(\log \log Y)$. If $X>Y^{2/3}J^{4/7}$, then the statement of the theorem follows from Proposition 5.6. If $X\leqslant Y^{2/3}J^{4/7}$, on the other hand, then it follows from taking $d=1$ in (5.6). $\square$

The rest of this section is concerned with proving Propositions  5.2, 5.3, 5.4 and 5.6. Propositions 5.2 and 5.3 will be proved using Lemmas  4.1 and 4.2 in Sect. 5.1. Proposition 5.4 will be proved in Sect. 5.2 and uses the circle method bound proved in Lemma 4.4. Finally, in Sect. 5.3 we shall prove Proposition 5.6. During the course of this work, given $X_1,X_2\geqslant 1$, we shall often use the elementary inequality

5.7 $$\begin{aligned} X_1+X_2\geqslant \max (X_1,X_2)\geqslant X_1^\alpha X_2^{1-\alpha }, \end{aligned}$$

for any $$0\leqslant \alpha \leqslant 1$$.

### Proof of Propositions 5.2 and 5.3

Let us begin by fixing some notation. Let $L_1,\dots ,L_4\in {\mathbb {Z}}[x_1,x_2]$ be the pairwise non-proportional linear forms featuring in (1.1). We define

5.8 $$\begin{aligned} {{\mathscr {D}}}=\prod _{1\leqslant i<j\leqslant 4}{{\,\mathrm{{\text {Res}}}\,}}(L_{i},L_{j}), \end{aligned}$$

where ${{\,\mathrm{{\text {Res}}}\,}}(L_i,L_j)$ is the resultant of $L_i$ and $L_j$, which is defined to be the absolute value of the determinant of the $2\times 2$ matrix formed from the coefficient vectors. Then, in what follows, we shall make frequent use of the fact that

5.9 $$\begin{aligned} \gcd (L_i(\textbf{x}),L_j(\textbf{x}))\mid {{\,\mathrm{{\text {Res}}}\,}}(L_i,L_j), \quad \text {for any}\ \textbf{x}\in {{\mathbb {Z}}}_{{\text {prim}}}^2, \end{aligned}$$

for each choice $i\ne j\in \{1,\dots ,4\}$. We shall also exploit the following elementary observation.

#### Lemma 5.7

Let $L_1,\dots ,L_4\in {\mathbb {Z}}[x_1,x_2]$ be pairwise non-proportional linear forms, as above. Let $\textbf{x}\in {{\mathbb {R}}}^2$ such that $|\textbf{x}|\sim X$. Then there exist $c_0\in (0,1)$ and $d_0>0$, both depending on the coefficients of $L_1,\dots ,L_4$, such that

$$\begin{aligned} |L_{i_0}(\textbf{x})|\leqslant c_0 X \Longrightarrow |L_i(\textbf{x})|\geqslant d_0 X \text { for every}\ i\ne i_0. \end{aligned}$$

#### Proof

Assume without loss of generality that $|\textbf{x}|=|x_1|$. Suppose that $|L_1(\textbf{x})|\leqslant c_1 X$, say, for a certain $0<c_1<1$, to be specified in due course. Then necessarily $L_1(\textbf{x})\ne \pm nx_1$ for any $n\in {{\mathbb {Z}}}_{\ne 0}$. Then, for $i\in \{2,3,4\}$, there exist $(\lambda _i,\mu _i)\in {\mathbb {Q}}\times {\mathbb {Q}}^\times$ such that $x_1=\lambda _iL_1(\textbf{x})+\mu _iL_i(\textbf{x})$. But then it follows that $X\leqslant |x_1| \leqslant |\lambda _i|c_1X+|\mu _i||L_i(\textbf{x})|,$ which implies that $|L_i(\textbf{x})|\geqslant |\mu _i|^{-1} (1-|\lambda _i|c_1)X$. The lemma follows on demanding that $c_1<1/|\lambda _i|$ for $2\leqslant i\leqslant 4$. $\square$

#### Proof of Proposition 5.3

We now estimate the cardinality of ${{\mathscr {M}}}_2(X,Y;D)$, as defined in (5.3). We can assume that $D\gg 1$ for an implied constant that depends on $L_1,\dots ,L_4$. According to the estimate following Lemma 2.7 in [5], the condition $\varDelta _{{\text {bad}}}(\textbf{x}) >D$ implies that either $\gcd (L_i(\textbf{x}),L_j(\textbf{x}))> D^\frac{1}{24}$ for certain indices $i\ne j$, or else $L_i(\textbf{x})^\square >D^\frac{1}{8}$. In view of (5.9), only the second possibility can happen if $D\gg 1$. Hence there exists $e\geqslant D^\frac{1}{16}$ and $i_0\in \{1,\dots ,4\}$ such that $e^2\mid L_{i_0}(\textbf{x})$. Without loss of generality we study the contribution corresponding to $i_0=1$. It therefore suffices to estimate the size of the set

5.10 $$\begin{aligned} {{\mathscr {M}}}_{2}(X,Y;e)=\{(\textbf{x},\textbf{y})\in {{\mathscr {M}}}(X,Y):e^2\mid L_{1}(\textbf{x})\}, \end{aligned}$$

for each $e\geqslant D^\frac{1}{16}$. On recalling (2.3) and (2.5), we see that any $(\textbf{x},\textbf{y})\in {{\mathscr {M}}}(X,Y)$ satisfies $L_1(\textbf{x})\ne 0$. Hence $e\ll X^\frac{1}{2}$, since $e^2\mid L_{1}(\textbf{x})$.

It will be convenient to define the set

5.11 $$\begin{aligned} {{\mathscr {S}}}(X)=\{\textbf{x}=(x_1,x_2)\in {\mathbb {Z}}_{\text {prim}}^2: |\textbf{x}|\leqslant 2X, (2.3) \text {holds}\}. \end{aligned}$$

Then for any $\textbf{x}\in {{\mathscr {S}}}(X)$, we may define the quaternary quadratic form

5.12 $$\begin{aligned} Q_{\textbf{x}}(\textbf{y})=L_1(\textbf{x})y_1^2+\dots +L_4(\textbf{x})y_4^2. \end{aligned}$$

Adopting the notation (4.1) and applying Lemma 4.2, we therefore obtain

$$\begin{aligned} \#{{\mathscr {M}}}_{2}(X,Y;e)&\ll _\varepsilon Y\sum _{\begin{array}{c} \textbf{x}\in {{\mathscr {S}}}(X)\\ e^2\mid L_{1}(\textbf{x}) \end{array}}1~+~Y^\varepsilon \sum _{\begin{array}{c} \textbf{x}\in {{\mathscr {S}}}(X) \\ e^2\mid L_{1}(\textbf{x}) \end{array}}\sum _{\begin{array}{c} {\textbf{c}}\in {{\mathbb {Z}}}_{{\text {prim}}}^4\\ |{\textbf{c}}|\ll Y^\frac{1}{3} \end{array}}1\\ {}&\quad +XY^{1+\varepsilon }\sum _{\begin{array}{c} \textbf{x}\in {{\mathscr {S}}}(X) \\ e^2\mid L_{1}(\textbf{x}) \end{array}}\sum _{\begin{array}{c} {\textbf{c}}\in {{\mathbb {Z}}}_{{\text {prim}}}^4\\ |{\textbf{c}}|\ll Y^\frac{1}{3},~Q^*_{\textbf{x}}({\textbf{c}})\ne 0 \end{array}}\frac{\gcd (\varDelta _{{\text {bad}}}(\textbf{x})^3,Q^*_{\textbf{x}}({\textbf{c}})^2)^\frac{1}{6}}{|{\textbf{c}}|\prod _{i=1}^4|L_i(\textbf{x})|^\frac{1}{2}}\\&=W_0(X,Y;e)+W_1(X,Y;e)+W_2(X,Y;e), \end{aligned}$$

say. On appealing to [12, Lemma 2], it easily follows that

5.13 $$\begin{aligned} W_0(X,Y;e)\ll \left( \frac{X^2}{e^2}+1\right) Y \quad \text { and } \quad W_1(X,Y;e)\ll \left( \frac{X^2}{e^2}+1\right) Y^{\frac{4}{3}+\varepsilon }.\qquad \end{aligned}$$

From now on we focus on estimating $W_2(X,Y;e)$. Let us introduce various dyadic parameters *S*, *T* corresponding respectively to the ranges of $\textbf{x}$ and ${\textbf{c}}$. Then, in the light of Lemma  5.7, we obtain

$$\begin{aligned} W_2(X,Y;e)\ll XY^{1+\varepsilon }\sum _{\begin{array}{c} S\nearrow X\\ T\nearrow Y^\frac{1}{3} \end{array}} \frac{1}{S^\frac{3}{2}T}\sum _{i_1=1}^{4}W_{2,i_1}(S,T;e), \end{aligned}$$

where

$$\begin{aligned} W_{2,i_1}(S,T;e)=\sum _{\begin{array}{c} \textbf{x}\in {{\mathscr {S}}}(X), ~e^2\mid L_{1}(\textbf{x})\\ |\textbf{x}|\sim S\\ i\ne i_1 \Rightarrow |L_{i}(\textbf{x})|\gg |L_{i_1}(\textbf{x})| \end{array}}\sum _{\begin{array}{c} {\textbf{c}}\in {{\mathbb {Z}}}_{{\text {prim}}}^4\\ |{\textbf{c}}|\sim T,~ Q^*_{\textbf{x}}({\textbf{c}})\ne 0 \end{array}}\frac{\gcd (\varDelta _{{\text {bad}}}(\textbf{x})^3,Q^*_{\textbf{x}}({\textbf{c}})^2)^\frac{1}{6}}{|L_{i_1}(\textbf{x})|^\frac{1}{2}}. \end{aligned}$$

By further decomposing the size of $L_{i_1}(\textbf{x})$ into dyadic intervals using a dyadic parameter *R*, we obtain

$$\begin{aligned} W_{2,i_1}(S,T;e)\leqslant \sum _{R \nearrow S}\frac{1}{R^\frac{1}{2}}W_{2,i_1}(R,S,T;e), \end{aligned}$$

where

5.14 $$\begin{aligned} W_{2,i_1}(R,S,T;e)=\sum _{\begin{array}{c} \textbf{x}\in {{\mathscr {S}}}(X), ~e^2\mid L_{1}(\textbf{x})\\ |\textbf{x}|\sim S,~ |L_{i_1}(\textbf{x})|\sim R \end{array}}\sum _{\begin{array}{c} {\textbf{c}}\in {{\mathbb {Z}}}_{{\text {prim}}}^4\\ |{\textbf{c}}|\sim T,~Q^*_{\textbf{x}}({\textbf{c}})\ne 0 \end{array}}\gcd (\varDelta _{{\text {bad}}}(\textbf{x})^3,Q^*_{\textbf{x}}({\textbf{c}})^2)^\frac{1}{6}.\nonumber \\ \end{aligned}$$

Recall the definition (5.8) of ${{\mathscr {D}}}$ and let $d\mid \gcd (\varDelta _{{\text {bad}}}(\textbf{x})^3,Q^*_{\textbf{x}}({\textbf{c}})^2)$. We claim that there exists $d_{\mathscr {D}}=O(1)$ and $d_1,\dots ,d_4\in {\mathbb {N}}$ such that $d_{\mathscr {D}}d=d_1\cdots d_4$ and $d_i\mid \gcd (L_i(\textbf{x})^3,c_i^6)$. If $d\mid \varDelta _{{\text {bad}}}(\textbf{x})^3$ then $d\mid (L_1(\textbf{x})\cdots L_4(\textbf{x}) )^3$. Let us put $d_i=\gcd (L_i(\textbf{x})^3,d)$ for $1\leqslant i\leqslant 4$. Then $\gcd (d_i,d_j)\mid {\mathscr {D}}$ for $i\ne j$. Hence $d_1\cdots d_4=d d_{{\mathscr {D}}}$, for a suitable positive integer $d_{\mathscr {D}}=O(1)$. It remains to prove that $d_i\mid c_i^6$. Suppose that $p^\lambda \Vert d_i$ with $\lambda \in {\mathbb {N}}$. Let $p^\nu \Vert L_1(\textbf{x})$. Then $\lambda \leqslant 3\nu$. On the other hand, we have $p^\lambda \mid Q^*_{\textbf{x}}({\textbf{c}})^2$, whence

$$\begin{aligned} p^{\lceil \frac{\lambda }{2}\rceil }\mid Q^*_{\textbf{x}}({\textbf{c}})&= \sum _{\{i,j,k,l\}=\{1,2,3,4\}}L_i(\textbf{x})L_j(\textbf{x})L_k(\textbf{x})c_l^2\\&=L_1(\textbf{x})\sum _{\{j,k,l\}=\{2,3,4\}}L_j(\textbf{x})L_k(\textbf{x})c_l^2+L_2(\textbf{x})L_3(\textbf{x})L_4(\textbf{x})c_1^2. \end{aligned}$$

Under the condition (2.3), it follows that $\prod _{i=1}^{4}L_i(\textbf{x})\ne 0$, whence $p^{\min (\nu ,\lceil \frac{\lambda }{2}\rceil )}\mid c_1^2$. If $\nu \leqslant \lceil \frac{\lambda }{2}\rceil$ then $p^{3\nu }\mid c_1^6$, whence $p^\lambda \mid c_1^6$. If $\nu > \lceil \frac{\lambda }{2}\rceil$, then $p^{\lceil \frac{\lambda }{2}\rceil }\mid c_1^2$, whence $p^{\lambda }\mid c_1^4$. This completes the proof of the claim.

We now continue estimating $W_{2,i_1}(R,S,T;e)$ in (5.14). Using the claim in the previous paragraph, we therefore obtain

$$\begin{aligned} W_{2,i_1}(R,S,T;e)&=\sum _{\begin{array}{c} \textbf{x}\in {{\mathscr {S}}}(X), ~e^2\mid L_{1}(\textbf{x})\\ |\textbf{x}|\sim S,~ |L_{i_1}(\textbf{x})|\sim R \end{array}} \sum _{\begin{array}{c} d\mid \varDelta _{{\text {bad}}}(\textbf{x})^3 \end{array}}d^\frac{1}{6}\sum _{\begin{array}{c} {\textbf{c}}\in {{\mathbb {Z}}}_{{\text {prim}}}^4,~|{\textbf{c}}|\sim T\\ d\mid Q^*_{\textbf{x}}({\textbf{c}})\ne 0 \end{array}}1\\&\ll \sum _{\begin{array}{c} \textbf{x}\in {{\mathscr {S}}}(X), ~e^2\mid L_{1}(\textbf{x})\\ |\textbf{x}|\sim S,~|L_{i_1}(\textbf{x})|\sim R \end{array}}\sum _{\begin{array}{c} d\mid \varDelta _{{\text {bad}}}(\textbf{x})^3 \end{array}} {d}^\frac{1}{6}\sum _{\begin{array}{c} d_{{\mathscr {D}}}d=d_1\cdots d_4\\ d_i\mid L_i(\textbf{x})^3 \end{array}} \sum _{\begin{array}{c} {\textbf{c}}\in {{\mathbb {Z}}}_{{\text {prim}}}^4,~|{\textbf{c}}|\sim T\\ d_i\mid c_i^6 \end{array}}1. \end{aligned}$$

The condition $|\textbf{x}|\sim S$ implies each $d_i\ll S^3$, since $\prod _{i=1}^{4}L_i(\textbf{x})\ne 0$. Hence

$$\begin{aligned} W_{2,i_1}(R,S,T;e) \ll \sum _{\begin{array}{c} \textbf{x}\in {{\mathscr {S}}}(X),~e^2\mid L_{1}(\textbf{x})\\ |\textbf{x}|\sim S,~|L_{i_1}(\textbf{x})|\sim R \end{array}}\sum _{\begin{array}{c} d\mid \varDelta _{{\text {bad}}}(\textbf{x})^3 \end{array}}d^\frac{1}{6}\sum _{\begin{array}{c} d_{{\mathscr {D}}}d=d_1\cdots d_4\\ \min _{1\leqslant i\leqslant 4}d_i\ll T^6\\ \max _{1\leqslant i\leqslant 4}d_i\ll S^2 \end{array}}\prod _{i=1}^{4}\left( \frac{T}{d_i^\frac{1}{6}}+1\right) , \end{aligned}$$

where the condition $\min _{1\leqslant i\leqslant 4}d_i\ll T^6$ is deduced from the fact that at least one of the components of ${\textbf{c}}$ must be non-zero. Therefore, for each factorisation $d_{{\mathscr {S}}}d=d_1\cdots d_4$, we have

$$\begin{aligned} d^\frac{1}{6}\prod _{i=1}^{4}\left( \frac{T}{d_i^\frac{1}{6}}+1\right)&\ll T^4+T^3\sum _{j=1}^4 d_j^\frac{1}{6}+ T^2 \hspace{-0.3cm} \sum _{k\ne l\in \{1,2,3,4\}}(d_kd_l)^\frac{1}{6}+T \hspace{-0.3cm} \sum _{\begin{array}{c} k,l,m\in \{1,2,3,4\}\\ \text {distinct} \end{array}}(d_kd_ld_m)^\frac{1}{6}\\ {}&\ll T^4+T^3S^\frac{1}{2}+T^2S+TS^\frac{3}{2}. \end{aligned}$$

This is $O(T^4+TS^\frac{3}{2})$. (Here, the absence of the constant term in the product is thanks to the fact that $\min _{1\leqslant i\leqslant 4}d_i\ll T^6$.)

Returning to $W_{2,i_1}(R,S,T;e)$ and using [12, Lemma 2] once more, we arrive at the bound

5.15 $$\begin{aligned} \begin{aligned} W_{2,i_1}(R,S,T;e)&\ll (T^4+TS^\frac{3}{2}) \sum _{\begin{array}{c} \textbf{x}\in {{\mathscr {S}}}(X),~e^2\mid L_{1}(\textbf{x})\\ |\textbf{x}|\sim S,~|L_{i_1}(\textbf{x})|\sim R \end{array}}\sum _{\begin{array}{c} d\mid \varDelta _{{\text {bad}}}(\textbf{x})^3 \end{array}} \tau _4(d)\\&\ll _\varepsilon (T^4+TS^\frac{3}{2}) \sum _{\begin{array}{c} \textbf{x}\in {{\mathscr {S}}}(X),~e^2\mid L_{1}(\textbf{x})\\ |\textbf{x}|\sim S,~|L_{i_1}(\textbf{x})|\sim R \end{array}}\varDelta _{{\text {bad}}}(\textbf{x})^\varepsilon \\&\ll _\varepsilon S^\varepsilon (T^4+TS^\frac{3}{2})\left( \frac{SR}{e^2}+1\right) , \end{aligned} \end{aligned}$$

for any $\varepsilon >0$. Summing over the dyadic parameter *R*, we therefore obtain

$$\begin{aligned} W_{2,i_1}(S,T;e)= \sum _{R \nearrow S}\frac{1}{R^\frac{1}{2}}W_{2,i_1}(R,S,T;e)\ll _\varepsilon S^{2\varepsilon }(T^4+TS^\frac{3}{2}) \left( \frac{S^{\frac{3}{2}}}{e^2}+1\right) . \end{aligned}$$

Recall that our dyadic parameter *S* goes to *X*, and *T* goes to $Y^\frac{1}{3}$. Moreover, we have seen that $e\ll S^\frac{1}{2}$, if $e^2\mid L_{1}(\textbf{x})$. Thus, on summing over *S*, *T*, we obtain

$$\begin{aligned} \sum _{S,T}\frac{1}{S^\frac{3}{2}T} \frac{S^{\frac{3}{2}+2\varepsilon }}{e^2}(T^4+TS^\frac{3}{2}) \ll (XY)^{2\varepsilon } \left( \frac{Y+X^\frac{3}{2}}{e^2}\right) \end{aligned}$$

and

$$\begin{aligned} \sum _{S,T}\frac{1}{S^\frac{3}{2}T} S^{2\varepsilon } (T^4+TS^\frac{3}{2})\ll X^{2\varepsilon }\sum _{T}\left( \frac{T^3}{e^3}+1\right) \ll (XY)^{2\varepsilon }\left( \frac{Y}{e^3}+1\right) . \end{aligned}$$

On redefining the choice of $\varepsilon$, we finally obtain

$$\begin{aligned} W_2(X,Y;e)\ll _\varepsilon (XY)^\varepsilon \left( \frac{XY^2+X^\frac{5}{2}Y}{e^2}+XY\right) . \end{aligned}$$

Taking $\alpha =\frac{1}{3}$ in (5.7), it follows that $X^2 Y^\frac{4}{3}\ll XY^2+X^\frac{5}{2}Y$. Hence, on combining our bound for $W_2(X,Y;e)$ with the contributions (5.13), we are led to the bound

$$\begin{aligned} \#{{\mathscr {M}}}_{2}(X,Y;e)\ll _\varepsilon (XY)^\varepsilon \left( \frac{XY^2+X^\frac{5}{2}Y}{e^2}+XY+Y^\frac{4}{3}\right) , \end{aligned}$$

for the cardinality of (5.10). Finally, it follows that

$$\begin{aligned} \#{{\mathscr {M}}}_2(X,Y;D)&\ll \hspace{-0.3cm} \sum _{D^\frac{1}{16}<e\ll \sqrt{X}} \hspace{-0.35cm} \#{{\mathscr {M}}}_{2}(X,Y;e) \ll _\varepsilon (XY)^\varepsilon \left( \frac{XY^2+X^\frac{5}{2}Y}{D^\frac{1}{16}}+X^\frac{3}{2}Y+X^\frac{1}{2}Y^{\frac{4}{3}}\right) . \end{aligned}$$

This completes the proof of Proposition 5.3. $\square$

Later in our argument we will also need to deal with summations over $\textbf{x}$ in which one of the linear forms $L_1,\dots ,L_4$ takes a particularly small value. We take this opportunity to prove the following analogue of [5, Lemma 2.1], whose proof is a minor modification of the one that we use to prove Proposition  5.3.

#### Lemma 5.8

Let $\varepsilon >0$. Define ${{\mathscr {M}}}_{i_1,\delta }(X,Y)=\{(\textbf{x},\textbf{y})\in {{\mathscr {M}}}(X,Y): |L_{i_1}(\textbf{x})|\leqslant |\textbf{x}|^\delta \}$, for any $\delta \in (0,1)$ and $i_1\in \{1,\dots ,4\}$, where ${{\mathscr {M}}}(X,Y)$ is given by (2.5). Then

$$\begin{aligned} \#{{\mathscr {M}}}_{i_1,\delta }(X,Y)\ll _{\varepsilon ,\delta } (XY)^\varepsilon (X^{\frac{1}{2}(1+\delta )} Y^2+X^{\frac{1}{2}(4+\delta )}Y). \end{aligned}$$

#### Proof

Assume without loss of generality that $i_1=1$. Then Lemma 5.7 implies that $|L_i(\textbf{x})|\gg _\delta X$ for any $i\in \{2,3,4\}$. We maintain the notation from the proof of Proposition 5.3. On recalling (5.14) and (5.15), we introduce an extra dyadic parameter *R* for the range of $L_{1}(\textbf{x})$ and similarly obtain

$$\begin{aligned} \#{{\mathscr {M}}}_{1,\delta }(X,Y)&\ll \sum _{\begin{array}{c} \textbf{x}\in {{\mathscr {S}}}(X)\\ |L_{1}(\textbf{x})|\ll X^\delta \end{array}} N(Q_{\textbf{x}};Y)\\&\ll _{\varepsilon ,\delta } X^{1+\delta }Y^{\frac{4}{3}+\varepsilon } +\frac{Y^{1+\varepsilon }}{X^\frac{1}{2}}\sum _{\begin{array}{c} R \nearrow X^\delta \\ T\nearrow Y^\frac{1}{3} \end{array}}\frac{1}{R^\frac{1}{2}T}W_{2,1}(R,X,T;1)\\&\ll _{\varepsilon ,\delta } X^{1+\delta }Y^{\frac{4}{3}+\varepsilon } + \frac{X^{1+\varepsilon } Y^{1+\varepsilon }}{X^\frac{1}{2}}\sum _{\begin{array}{c} R \nearrow X^\delta \\ T\nearrow Y^\frac{1}{3} \end{array}}\frac{R(T^4+TX^\frac{3}{2})}{R^\frac{1}{2}T}\\&\ll _{\varepsilon ,\delta } (XY)^\varepsilon (X^{1+\delta }Y^\frac{4}{3}+X^{\frac{1}{2}(1+\delta )} Y^2+X^{\frac{1}{2}(4+\delta )}Y). \end{aligned}$$

Using (5.7) with $\alpha =\frac{1}{3}$, we have $X^{\frac{1}{2}(1+\delta )} Y^2+X^{\frac{1}{2}(4+\delta )}Y\geqslant X^{1+\delta }Y^\frac{4}{3}.$ $\square$

It is now time to analyse the size of the set ${{\mathscr {M}}}_1(X,Y;D)$ that was defined in (5.2).

#### Proof of Proposition 5.2

We note that

$$\begin{aligned} \#{{\mathscr {M}}}_1(X,Y;D)=\sum _{\textbf{x}\in {{\mathscr {S}}}(X), ~\varDelta _{{\text {bad}}}(\textbf{x})\leqslant D}N(Q_\textbf{x};Y), \end{aligned}$$

where ${{\mathscr {S}}}(X)$ is given by (5.11), $Q_\textbf{x}$ by (5.12) and $N(Q_\textbf{x};Y)$ is the counting function defined in (4.1). Let us put

5.16 $$\begin{aligned} \Vert Q_\textbf{x}\Vert =\max _{1\leqslant i\leqslant 4}|L_i(\textbf{x})|,\quad \varDelta (\textbf{x})=\prod _{i=1}^{4}L_i(\textbf{x}). \end{aligned}$$

Under the assumption that $\varDelta _{{\text {bad}}}(\textbf{x})\leqslant Y^\frac{1}{20}$, it follows from Lemma 4.1 that

$$\begin{aligned} N(Q_\textbf{x};Y) \ll _\varepsilon \varpi \left( \Delta (\textbf{x})\right) \varDelta _{{\text {bad}}}(\textbf{x})^{\frac{1}{4}+\varepsilon } \left( Y^\frac{4}{3}N_1(\textbf{x},Y)+Y^2N_2(\textbf{x},Y)\right) , \end{aligned}$$

for any $\varepsilon >0$, where the arithmetic function $\varpi$ is defined by (4.2), and

$$\begin{aligned} N_1(\textbf{x},Y)=\frac{\Vert Q_\textbf{x}\Vert ^\frac{5}{2}}{|\varDelta (\textbf{x})|^\frac{5}{8}}L(\sigma _Y,\chi _{Q_\textbf{x}}) \quad \text { and } \quad N_2(\textbf{x},Y)=\frac{\Vert Q_\textbf{x}\Vert ^\frac{5}{2}}{|\varDelta (\textbf{x})|^\frac{7}{8}}L(\sigma _Y,\chi _{Q_\textbf{x}}). \end{aligned}$$

We can alternatively write $\varpi (m)=\sum _{t\mid m}\frac{\mu ^2(t)}{t}.$ Under the assumption $D\leqslant Y^\frac{1}{20}$, it therefore follows that

$$\begin{aligned} \#{{\mathscr {M}}}_1(X,Y;D)&\ll _\varepsilon \sum _{\begin{array}{c} r\in {{\mathbb {N}}}\\ r~\square \text {-full}\\ r\leqslant D \end{array}}r^{\frac{1}{4}+\varepsilon }\sum _{\begin{array}{c} \textbf{x}\in {{\mathscr {S}}}(X)\\ \varDelta _{{\text {bad}}}(\textbf{x})=r \end{array}}\left( \sum _{\begin{array}{c} t\mid \varDelta (\textbf{x}) \end{array}}\frac{\mu ^2(t)}{t}\right) \left( Y^\frac{4}{3}N_1(\textbf{x},Y)+Y^2N_2(\textbf{x},Y)\right) . \end{aligned}$$

Hence

5.17 $$\begin{aligned} \#{{\mathscr {M}}}_1(X,Y;D)\ll \sum _{\begin{array}{c} r,t\in {{\mathbb {N}}}\\ t~\square \text {-free},~t\ll X^4\\ r~\square \text {-full},~r\leqslant D \end{array}}\frac{r^{\frac{1}{4}+\varepsilon }}{t} \sum _{\begin{array}{c} {\textbf{d}}\in {{\mathbb {N}}}^4\\ \ d_1\cdots d_4=[r,t] \end{array}}N_{{\textbf{d}}}(X,Y), \end{aligned}$$

where

$$\begin{aligned} N_{{\textbf{d}}}(X,Y)=\sum _{\begin{array}{c} \textbf{x}\in {{\mathscr {S}}}(X)\\ d_i\mid L_i(\textbf{x}) \end{array}}\left( Y^\frac{4}{3}N_1(\textbf{x},Y)+Y^2N_2(\textbf{x},Y)\right) . \end{aligned}$$

We recall that

$$\begin{aligned} L(\sigma _Y,\chi _{Q_\textbf{x}})=\sum _{n=1}^{\infty }\frac{\chi _{Q_\textbf{x}}(n)}{n^{\sigma _Y}}, \end{aligned}$$

where $\sigma _Y=1+\frac{1}{\log Y}>1$. We have $L(\sigma _Y,\chi _{Q_\textbf{x}})\geqslant 0$. By Lemma 5.7 we can assume without loss of generality that $|L_i(\textbf{x})|\gg |L_1(\textbf{x})|$ for $i\geqslant 2$. On introducing dyadic decomposition parameters *S* for $|\textbf{x}|$ and *R* for $|L_{1}(\textbf{x})|$, and on dropping the primitivity condition on $\textbf{x}$, it follows that

5.18 $$\begin{aligned} N_{{\textbf{d}}}(X,Y) \ll \sum _{S\nearrow X}\sum _{R \nearrow S}\left( \frac{S^\frac{5}{8}Y^\frac{4}{3}}{R^\frac{5}{8}}+\frac{Y^2}{S^\frac{1}{8}R^\frac{7}{8}}\right) N_{{\textbf{d}}}(S,R,Y), \end{aligned}$$

where

5.19 $$\begin{aligned} \begin{aligned} N_{{\textbf{d}}}(S,R,Y)&=\sum _{\begin{array}{c} \textbf{x}\in {{\mathbb {Z}}}^2\\ |\textbf{x}|\sim S,~|L_{1}(\textbf{x})|\sim R\\ \prod _{i=1}^{4}L_i(\textbf{x})\ne \square \\ d_{i}\mid L_i(\textbf{x}) \end{array}} \sum _{n=1}^{\infty }\frac{\chi _{Q_\textbf{x}}(n)}{n^{\sigma _Y}}. \end{aligned} \end{aligned}$$

In particular, we may assume that $d_1\cdots d_4\ll S^4$. We have $L_i(\textbf{x})=a_ix_1+b_ix_2$ for $1\leqslant i\leqslant 4$, where $\gcd (a_i,b_i)=1$. But then there exists a matrix $\textbf{M}\in \textrm{SL}_2({\mathbb {Z}})$ with first row equal to $(a_1,b_1)$. Making the change of variables $\textbf{y}=\textbf{M}\textbf{x}$, we let $J_i(\textbf{y})=L_i(\textbf{M}^{-1}\textbf{y})$, for $1\leqslant i\leqslant 4$. We can thus rewrite (5.19) as

$$\begin{aligned} N_{{\textbf{d}}}(S,R,Y)= \sum _{\begin{array}{c} \textbf{y}\in {{\mathbb {Z}}}^2\\ |y_1|\sim R, ~|\textbf{M}^{-1}\textbf{y}|\sim S\\ \prod _{i=1}^{4}J_i(\textbf{y})\ne \square \\ d_{i}\mid J_i(\textbf{y}) \end{array}}\sum _{n=1}^{\infty }\frac{\chi _{\textbf{y}}(n)}{n^{\sigma _Y}}, \end{aligned}$$

where $$ \chi _{\textbf{y}}(\cdot )=(\frac{J_1(\textbf{y})\cdots J_4(\textbf{y})}{\cdot }). $$

Let $\theta >\frac{3}{16}$. We introduce the truncation parameter

5.20 $$\begin{aligned} N_1=(S^3R)^{2\theta }. \end{aligned}$$

Then since $\prod _{i=1}^{4}J_i(\textbf{y})\ne \square$, the character $\chi _{\textbf{y}}$ is a non-principal Dirichlet character of modulus at most $\prod _{i=1}^{4}|J_i(\textbf{y})|\ll \prod _{i=1}^{4}|L_i(\textbf{x})|\ll S^3R.$ The Burgess bound in Lemma 3.1 implies that

$$\begin{aligned} \sum _{n>N_1}\frac{\chi _{\textbf{y}}(n)}{n^{\sigma _Y}}\ll _\theta N_1^{-\frac{1}{2}}(S^3R)^\theta \ll 1. \end{aligned}$$

Thus it follows that

5.21 $$\begin{aligned} \sum _{\begin{array}{c} \textbf{y}\in {{\mathbb {Z}}}^2\\ |y_1|\sim R, ~|\textbf{M}^{-1}\textbf{y}|\sim S\\ \prod _{i=1}^{4}J_i(\textbf{y})\ne \square \\ d_{i}\mid J_i(\textbf{y}) \end{array}} \sum _{n>N_1}\frac{\chi _{\textbf{y}}(n)}{n^{\sigma _Y}}\ll _\theta \frac{SR}{d_1\cdots d_4}+S. \end{aligned}$$

It remains to study the contribution

$$\begin{aligned} \sum _{\begin{array}{c} \textbf{y}\in {{\mathbb {Z}}}^2\\ |y_1|\sim R, ~|\textbf{M}^{-1}\textbf{y}|\sim S\\ \prod _{i=1}^{4}J_i(\textbf{y})\ne \square \\ d_{i}\mid J_i(\textbf{y}) \end{array}} \sum _{n\leqslant N_1}\frac{\chi _{\textbf{y}}(n)}{n^{\sigma _Y}}. \end{aligned}$$

We observe that $\gcd (d_i,d_j)\mid {{\mathscr {D}}}$ for each $1\leqslant i< j\leqslant 4$. We need to separate out the contribution from those $\textbf{y}$ for which $\prod _{i=1}^{4}J_i(\textbf{y})=\square$. For such $\textbf{y}$, the *n*-sum contributes $O_\varepsilon (N_1^\varepsilon ).$ Moreover, there are $O(Y^\varepsilon )$ primitive vectors $|\textbf{y}|\leqslant Y$ for which $\prod _{i=1}^{4}J_i(\textbf{y})=\square$, by the proof of Lemma  5.1, leading to an overall contribution $O_\varepsilon (S^{1+\varepsilon }),$ on extracting possible common divisors from $y_1$ and $y_2$. Hence

5.22 $$\begin{aligned} \sum _{\begin{array}{c} \textbf{y}\in {{\mathbb {Z}}}^2\\ |y_1|\sim R, ~|\textbf{M}^{-1}\textbf{y}|\sim S\\ \prod _{i=1}^{4}J_i(\textbf{y})\ne \square \\ d_{i}\mid J_i(\textbf{y}) \end{array}} \sum _{n\leqslant N_1}\frac{\chi _{\textbf{y}}(n)}{n^{\sigma _Y}} = O_\varepsilon (S^{1+\varepsilon } N_1^\varepsilon )+ \sum _{n\leqslant N_1}\frac{1}{n^{\sigma _Y}}T_{{\textbf{d}}}(n;S,R), \end{aligned}$$

where

$$\begin{aligned} T_{{\textbf{d}}}(n;S,R)=\sum _{ \begin{array}{c} \textbf{y}\in {{\mathbb {Z}}}^2\\ |y_1|\sim R, ~|\textbf{M}^{-1}\textbf{y}|\sim S\\ d_{i}\mid J_i(\textbf{y}) \end{array}} \left( \frac{J_1(\textbf{y})\cdots J_4(\textbf{y})}{n}\right) . \end{aligned}$$

Note that once $y_1$ is fixed, there exists an interval $K_{y_1}$ of length *O*(*S*), such that $|\textbf{M}^{-1}\textbf{y}|\sim S$ if and only if $y_2\in K_{y_1}$. For fixed $y_1$, we are now in a position to apply Lemma 3.2 to estimate the character sum involving $y_2$. Note that there exists a factorisation $n=n_0n_1^2$, with $n_0$ square-free. Applying Lemma  3.2 with $A={{\mathscr {D}}}$, $q=1$ and $k=4$, we therefore obtain

$$\begin{aligned} T_{{\textbf{d}}}(n;S,R)&\ll _\varepsilon \sum _{|y_1|\sim R} \left( \frac{S}{n_0^{\frac{1}{2}}d_1\cdots d_4} +n_0^{\frac{1}{2}} \log (d_1\cdots d_4)\right) n_0^{\varepsilon /2} \gcd (y_1,n_0d_1\cdots d_4)\\&\ll _\varepsilon (d_1\cdots d_4n_0)^\varepsilon \left( \frac{RS}{n_0^{\frac{1}{2}}d_1\cdots d_4} +n_0^{\frac{1}{2}}R\right) , \end{aligned}$$

for any $\varepsilon >0$, since $\sum _{y\leqslant Y}\gcd (y,a)\leqslant \sum _{d\mid a} d\#\{y\leqslant Y: d\mid y\}\leqslant \tau (a)Y,$ for any $a\in {\mathbb {N}}$. Since $d_1\cdots d_4\ll S^4$, it now follows that

$$\begin{aligned} \sum _{n\leqslant N_1}\frac{1}{n^{\sigma _Y}}T_{{\textbf{d}}}(n;S,R)&\ll _\varepsilon (d_1\cdots d_4)^\varepsilon \sum _{n_0\leqslant N_1} \frac{1}{n_0} \left( \frac{SR}{n_0^{\frac{1}{2}-\varepsilon }d_1\cdots d_4}+Rn_0^{\frac{1}{2}+\varepsilon } \right) \\&\ll _\varepsilon \frac{SR}{(d_1\cdots d_4)^{1-\varepsilon }}+R N_1^{\frac{1}{2}+\varepsilon }S^{4\varepsilon }. \end{aligned}$$

We may now record our final estimate for (5.19). Combining the previous line with (5.21) and (5.22), and recalling the choice of $N_1$ made in (5.20), we therefore deduce that

$$\begin{aligned} N_{{\textbf{d}}}(S,R,Y)&\ll _{\varepsilon , \theta } \frac{SR}{(d_1\cdots d_4)^{1-\varepsilon }}+S+ S^{1+\varepsilon } N_1^\varepsilon + R N_1^{\frac{1}{2}+\varepsilon }S^{4\varepsilon } \\&\ll _{\varepsilon , \theta } \frac{SR}{(d_1\cdots d_4)^{1-\varepsilon }}+ S^{1+2\varepsilon } + R (S^3R)^{\theta }S^{8\varepsilon }. \end{aligned}$$

On rescaling $\varepsilon$, we finally obtain

5.23 $$\begin{aligned} N_{{\textbf{d}}}(S,R,Y)\ll _{\varepsilon ,\theta } \frac{SR}{(d_1\cdots d_4)^{1-\varepsilon }}+ S^{\varepsilon }\left( S+S^{3\theta }R^{1+\theta }\right) , \end{aligned}$$

for any $$\theta >\frac{3}{16}$$.

We are now ready to sum over all dyadic intervals in (5.18). Inserting (5.23), it follows that

$$\begin{aligned} N_{{\textbf{d}}}(X,Y)&\ll _{\varepsilon ,\theta } \sum _{S\nearrow X}\sum _{R \nearrow S}\left( \frac{S^\frac{5}{8}Y^\frac{4}{3}}{R^\frac{5}{8}}+\frac{Y^2}{S^\frac{1}{8}R^\frac{7}{8}}\right) \left( \frac{SR}{(d_1\cdots d_4)^{1-\varepsilon }}+ S^{\varepsilon }\left( S+S^{3\theta }R^{1+\theta }\right) \right) \\&\ll _{\varepsilon ,\theta } Y^\frac{4}{3}\left( \frac{X^2}{(d_1\cdots d_4)^{1-\varepsilon }}+X^\varepsilon \left( X^{\frac{13}{8}}+X^{1+4\theta }\right) \right) \\&\qquad +Y^2\left( \frac{X}{(d_1\cdots d_4)^{1-\varepsilon }}+X^\varepsilon \left( X^{\frac{7}{8}}+X^{4\theta }\right) \right) . \end{aligned}$$

Making the choice $\theta =\frac{7}{32}$, we obtain

$$\begin{aligned} N_{{\textbf{d}}}(X,Y)\ll _{\varepsilon } \frac{X^2Y^\frac{4}{3}+XY^2}{(d_1\cdots d_4)^{1-\varepsilon }}+X^\varepsilon \left( X^\frac{15}{8}Y^\frac{4}{3}+X^\frac{7}{8}Y^2\right) \end{aligned}$$

in (5.17). It remains to sum over all *r*, *t*. Using the trivial bound for the divisor function $\tau _4$, we obtain

$$\begin{aligned} \sum _{\begin{array}{c} r,t\in {{\mathbb {N}}}\\ t~\square \text {-free},~t\ll X^4\\ r~\square \text {-full},~r\leqslant D \end{array}}\frac{r^{\frac{1}{4}+\varepsilon }}{t} \tau _4([r,t])\ll _\varepsilon X^\varepsilon D^{\frac{3}{4}+\varepsilon }. \end{aligned}$$

Similarly, in view of the lower bound $[r,t]\geqslant \max (r,t)\geqslant r^\frac{7}{8} t^\frac{1}{8}$, we have

$$\begin{aligned} \sum _{\begin{array}{c} r,t\in {{\mathbb {N}}}\\ t~\square \text {-free},~t\ll X^4\\ r~\square \text {-full},~r\leqslant D \end{array}}\frac{r^{\frac{1}{4}+\varepsilon }}{t} \frac{\tau _4([r,t])}{[r,t]^{1-\varepsilon }}\ll _\varepsilon \sum _{\begin{array}{c} r,t\in {{\mathbb {N}}}\\ t~\square \text {-free},~t\ll X^4\\ r~\square \text {-full},~r\leqslant D \end{array}}\frac{1}{r^{\frac{5}{8}-2\varepsilon }t^{\frac{9}{8}-2\varepsilon }} \ll _\varepsilon 1. \end{aligned}$$

The statement of Proposition 5.2 is now clear. $\square$

### Proof of Proposition 5.4

Recall the definition (5.11) of ${{\mathscr {S}}}(X)$. Then we have

$$\begin{aligned} \#{{\mathscr {M}}}(X,Y)=\sum _{\begin{array}{c} \textbf{x}\in {{\mathscr {S}}}(X) \end{array}}N(Q_\textbf{x};Y), \end{aligned}$$

where $Q_\textbf{x}$ is given by (5.12) and $N(Q_\textbf{x};Y)$ is the counting function defined in (4.1). We continue to adopt the notation $\Vert Q_\textbf{x}\Vert$ and $\varDelta (\textbf{x})$ that was introduced in (5.16). In this section we see what can be deduced from an application of Lemma 4.4. Letting $m(Q_\textbf{x})=\min _{1\leqslant i\leqslant 4} |L_i(\textbf{x})|$, we obtain

$$\begin{aligned} N(Q_\textbf{x};Y)\ll _\varepsilon \frac{ \varDelta _{{\text {bad}}}(\textbf{x})^\varepsilon L(1,\chi _{Q_\textbf{x}}) }{(m(Q_\textbf{x})\Vert Q_\textbf{x}\Vert )^\frac{1}{2}}Y^2+\frac{\Vert Q_\textbf{x}\Vert ^{11+\varepsilon }}{m(Q_\textbf{x})^6| \varDelta (\textbf{x})|^\frac{1}{2}}Y^{\frac{3}{2}+\varepsilon }, \end{aligned}$$

for any $\varepsilon >0$. Lemma 5.7 implies that $|\Delta (\textbf{x})|^{\frac{1}{2}}\gg \Vert Q_\textbf{x}\Vert ^{\frac{3}{2}} m(Q_\textbf{x})^{\frac{1}{2}}.$ Since $\Vert Q_\textbf{x}\Vert \ll X$, it follows that

5.24 $$\begin{aligned} \#{{\mathscr {M}}}(X,Y)\ll _\varepsilon Y^2\sum _{\begin{array}{c} \textbf{x}\in {{\mathscr {S}}}(X) \end{array}} \frac{\varDelta _{{\text {bad}}}(\textbf{x})^\varepsilon L(1,\chi _{Q_\textbf{x}})}{(m(Q_{\textbf{x}})\Vert Q_\textbf{x}\Vert )^\frac{1}{2}} + X^{\frac{19}{2}+\varepsilon }Y^{\frac{3}{2}+\varepsilon } \hspace{-0.2cm} \sum _{\begin{array}{c} \textbf{x}\in {{\mathscr {S}}}(X) \end{array}}\frac{1}{m(Q_{\textbf{x}})^{\frac{13}{2}}}.\nonumber \\ \end{aligned}$$

But a standard dyadic decomposition procedure yields

$$\begin{aligned} \sum _{\begin{array}{c} \textbf{x}\in {{\mathscr {S}}}(X) \end{array}}\frac{1}{m(Q_{\textbf{x}})^{\frac{13}{2}}}&\ll \sum _{i_1=1}^4\sum _{S\nearrow X}\frac{1}{S^\frac{13}{2}} \#\{\textbf{x}\in {{\mathscr {S}}}(X): |L_{i_1}(\textbf{x})|\sim S\} \ll X. \end{aligned}$$

Thus the overall contribution from the second term is $\ll _\varepsilon X^{\frac{21}{2}+\varepsilon } Y^{\frac{3}{2}+\varepsilon }\ll X^{11} Y^{\frac{3}{2}+\varepsilon }.$

In what follows we focus on the first summand. Much as in the proof of Proposition 5.2, we carry out two dyadic decompositions. Then, for fixed $i_1\in \{1,\dots ,4\}$, we are reduced to estimating

5.25 $$\begin{aligned} \sum _{S\nearrow X}\sum _{R \nearrow S}\frac{1}{S^\frac{1}{2}R^\frac{1}{2}} W_{i_1}(S,R), \end{aligned}$$

where

5.26 $$\begin{aligned} W_{i_1}(S,R)= \sum _{\begin{array}{c} \textbf{x}\in {{\mathscr {S}}}(X)\\ |\textbf{x}|\sim S,~|L_{i_1}(\textbf{x})|\sim R \end{array}}\varDelta _{{\text {bad}}}(\textbf{x})^\varepsilon L(1,\chi _{Q_\textbf{x}}). \end{aligned}$$

One of the ingredients we will need in our treatment of $W_{i_1}(S,R)$ is a proof that there are relatively few $\textbf{x}$ for which $\varDelta _{{\text {bad}}}(\textbf{x})$ is large. This is achieved in the following result.

#### Lemma 5.9

Let $\delta \geqslant 0$ and let $i_1\in \{1,\dots ,4\}$. Then

$$\begin{aligned} \#\left\{ \textbf{x}\in {\mathbb {Z}}_{\text {prim}}^2: |\textbf{x}|\sim S,~|L_{i_1}(\textbf{x})|\sim R, ~\varDelta _{{\text {bad}}}(\textbf{x})>(SR)^\delta \right\} \ll (SR)^{1-\frac{\delta }{8}}. \end{aligned}$$

#### Proof

Let $N_\delta (S,R)$ denote the quantity that is to be estimated. First, we observe that the condition $\varDelta _{{\text {bad}}}(\textbf{x})>(SR)^\delta$ implies that at least one of $L_1(\textbf{x}),\dots , L_4(\textbf{x})$ has a square-full part that exceeds $(SR)^\frac{\delta }{4}$. Let us assume that $L_{i_0}$ has square-full part $>(SR)^\frac{\delta }{4}$, with $i_0\ne i_1$. Upon a non-singular change of variables, we may therefore assume that $L_{i_0}(\textbf{x})=x_1$ and $L_{i_1}(\textbf{x})=x_2$. But then, on summing trivially over $x_2$, we obtain

$$\begin{aligned} N_\delta (S,R)&\ll R\sum _{\begin{array}{c} (SR)^\frac{\delta }{4}\ll a\ll S\\ a~\square \text {-full} \end{array}}\sum _{\begin{array}{c} x_1\in {{\mathbb {Z}}}_{\ne 0}\\ |x_1|\leqslant S,~a\mid x_1 \end{array}} 1 \ll (SR)^{1-\frac{\delta }{8}}, \end{aligned}$$

which is satisfactory. Alternatively, if $L_{i_1}(\textbf{x})$ is the term with large square-full part, then a similar manipulation yields

$$\begin{aligned} N_\delta (S,R)&\ll S\sum _{\begin{array}{c} (SR)^\frac{\delta }{4}\ll a\ll R\\ a~\square \text {-full} \end{array}}\sum _{\begin{array}{c} x_1\in {{\mathbb {Z}}}_{\ne 0}\\ |x_1|\leqslant R,~a\mid x_1 \end{array}} 1 \ll (SR)^{1-\frac{\delta }{8}}, \end{aligned}$$

which completes the proof of the lemma. $\square$

We are now ready to estimate the quantity in (5.26).

#### Lemma 5.10

Let $$\delta \geqslant 0$$. Then

$$\begin{aligned} W_{i_1}(S,R)\ll _{\varepsilon }SR+S^\varepsilon \left( (SR)^{\frac{\delta }{2}} \left( S+S^{\frac{21}{32}}R^{\frac{39}{32}}\right) +(SR)^{1-\frac{\delta }{8}}\right) . \end{aligned}$$

#### Proof

For given $\delta \geqslant 0$, we write

$$\begin{aligned} W_{i_1}(S,R)=W_{i_1,1}(S,R;\delta )+W_{i_1,2}(S,R;\delta ), \end{aligned}$$

where the sum $W_{i_1,1}(S,R;\delta )$ is subject to the condition $\varDelta _{{\text {bad}}}(\textbf{x})\leqslant (SR)^\delta$ and $W_{i_1,2}(S,R;\delta )$ has $\varDelta _{{\text {bad}}}(\textbf{x})>(SR)^\delta$.

Observe that

5.27 $$\begin{aligned} \varDelta _{{\text {bad}}}(\textbf{x})^\varepsilon L(1,\chi _{Q_\textbf{x}}) \ll |\textbf{x}|^{4\varepsilon } \log \left( 2+\Vert Q_\textbf{x}\Vert \right) \ll _{\varepsilon } X^{5\varepsilon }. \end{aligned}$$

Rescaling $\varepsilon$ and applying Lemma 5.9, we get a satisfactory bound for the term $W_{i_1,2}(S,R;\delta )$. Turning to $W_{i_1,1}(S,R;\delta )$, we begin in the same way as in the proof of Proposition 5.2. Thus

$$\begin{aligned} W_{i_1,1}(S,R;\delta )&\leqslant \sum _{\begin{array}{c} d\leqslant (SR)^\delta \\ d~\square \text {-full} \end{array}}d^\varepsilon \sum _{\begin{array}{c} {\textbf{d}}\in {\mathbb {N}}^4\\ d=d_1\cdots d_4 \end{array}}N^{\prime }_{i_1,{\textbf{d}}}(S,R), \end{aligned}$$

where

$$\begin{aligned} N^{\prime }_{i_1,{\textbf{d}}}(S,R)=\sum _{\begin{array}{c} \textbf{x}\in {{\mathscr {S}}}(X)\\ |\textbf{x}|\sim S,~|L_{i_1}(\textbf{x})|\sim R\\ d_{i}\mid L_i(\textbf{x}) \end{array}}L(1,\chi _{Q_\textbf{x}}). \end{aligned}$$

This is essentially the same sum that we already met in (5.19) and we can directly apply the bound (5.23). Taking $\theta =\frac{7}{32}$, we get

$$\begin{aligned} N^{\prime }_{i_1,{\textbf{d}}}(S,R)\ll _{\varepsilon } \frac{SR}{(d_1\cdots d_4)^{1-\varepsilon }}+ S^{\varepsilon }\left( S+S^{\frac{21}{32}}R^{\frac{39}{32}}\right) , \end{aligned}$$

whence $$ W_{i_1,1}(S,R;\delta )\ll _{\varepsilon } SR+S^\varepsilon (SR)^{\frac{\delta }{2}}(S+S^{\frac{21}{32}}R^{\frac{39}{32}}). $$ $$\square $$

Inserting Lemma 5.10 into (5.25) and summing over dyadic intervals, we are now led to the conclusion that

$$\begin{aligned} \sum _{S\nearrow X}\sum _{R \nearrow S}\frac{1}{S^\frac{1}{2}R^\frac{1}{2}}W_{i_1}(S,R)\ll _{\varepsilon } X+X^\varepsilon \left( X^{\frac{7}{8}+\delta }+X^{1-\frac{\delta }{4}}\right) . \end{aligned}$$

Taking $\delta =\frac{1}{10}$, the right hand side is *O*(*X*), on taking $\varepsilon$ sufficiently small. Therefore the overall contribution of the first term in (5.24) is $O(XY^2)$, thereby completing the proof of Proposition  5.2.

### Proof of Proposition 5.6

Our proof of Proposition 5.6 relies on viewing the equation (1.1) in the form (1.3), where $Q_1,Q_2$ are the diagonal quadratic forms defined in (2.2), where $\gcd (a_i,b_i)=1$ for $1\leqslant i\leqslant 4$. As usual, we proceed under the assumption that the pencil $Q_1=Q_2=0$ defines a smooth curve $Z\subset {\mathbb {P}}^3$ of genus 1, such that $Z({\mathbb {R}})= \emptyset$.

We will be led to make crucial use of properties of the lattice $\Lambda _{[\textbf{u}],k}$ that was defined in (3.9). In particular, we will need to show that its successive minima are not typically lop-sided. We begin, however, with the following basic estimate.

#### Lemma 5.11

Let $\varepsilon >0$ and let $D,E>0$ such that $1\ll E\ll D^{3/4}.$ Then

$$\begin{aligned} \sum _{d\asymp D}\sum _{\begin{array}{c} \textbf{v}\in {\mathbb {Z}}^4\\ |\textbf{v}|\leqslant E\\ d\mid Q_{i}(\textbf{v}), ~i=1,2 \end{array}}1\ll E^{2+\varepsilon } + \frac{E^{4}}{D}\cdot \frac{\log E}{\log \log E}. \end{aligned}$$

#### Proof

If the left hand side is non-zero, then there exists a vector $\textbf{v}\in {\mathbb {Z}}^4$ such that $|\textbf{v}|\leqslant E$ and $d\mid Q_i(\textbf{v})$ for $i=1,2$. But then $D\ll E^2$, since $Q_1(\textbf{v}), Q_2(\textbf{v})$ cannot both vanish. Thus we may proceed under the assumption that $D\ll E^2$.

We sort the sum on the left according to the value of $\gcd (Q_{1}(\textbf{v}), Q_{2}(\textbf{v}))$. This gives

$$\begin{aligned} \sum _{d\asymp D}\sum _{\begin{array}{c} |\textbf{v}|\leqslant E\\ d|Q_{i}(\textbf{v})\text { for }i=1,2 \end{array}}1= \sum _{c=0}^{\log (ME^{2}/D)}\sum _{g\sim 2^{c}D}\tau _{D}(g)\sum _{\begin{array}{c} |\textbf{v}|\leqslant E\\ g=\gcd (Q_{1}(\textbf{v}),Q_{2}(\textbf{v})) \end{array}}1, \end{aligned}$$

where $\tau _{D}(g)=\#\{d\asymp D: d\mid g\}$ and $M=\max \left( |a_{1}|+\cdots +|a_4|,|b_{1}|+\cdots +|b_4|\right) .$ We claim that

$$\begin{aligned} \tau _{D}(g)\ll \min \left( 2^c, {\text {e}}^{\frac{2\log E}{\log \log E}}\right) . \end{aligned}$$

Indeed, if $g\in [2^{c}D,2^{c+1}D)$ and $g=fd$ for some $d\asymp D$, then $2^{c}\ll f\leqslant 2^{c}$. This implies that $\tau _{D}(g)\ll 2^{c}$. On the other hand,

$$\begin{aligned} \tau _{D}(g)\leqslant \tau (g)\leqslant {\text {e}}^{(\log 2+o(1))\frac{\log g}{\log \log g}}\ll {\text {e}}^{\frac{\log g}{\log \log g}}, \end{aligned}$$

by Tenenbaum [27, Thm. 5.4], for example. The claim follows, since $g\leqslant ME^{2}$.

Next, we observe that if $g=\gcd (Q_{1}(\textbf{v}),Q_{2}(\textbf{v}))$ then

$$\begin{aligned} \frac{Q_{1}(\textbf{v})}{g}\cdot Q_{2}(\textbf{v})=Q_{1}(\textbf{v})\cdot \frac{Q_{2}(\textbf{v})}{g}, \end{aligned}$$

where $\gcd (g^{-1}Q_{1}(\textbf{v}),g^{-1}Q_{2}(\textbf{v}))=1$. Moreover, we also have

$$\begin{aligned} \max \left( \frac{|Q_{1}(\textbf{v})|}{g},\frac{|Q_{2}(\textbf{v})|}{g}\right) \leqslant \frac{ME^{2}}{2^{c}D}. \end{aligned}$$

It follows that

5.28 $$\begin{aligned} \sum _{g\sim 2^{c}D}\sum _{\begin{array}{c} |\textbf{v}|\leqslant E\\ g=\gcd (Q_{1}(\textbf{v}),Q_{2}(\textbf{v})) \end{array}}1 \leqslant \#{{\mathscr {M}}}^*\left( \frac{ME^{2}}{2^{c}D}, E\right) , \end{aligned}$$

in the notation of (5.4). Hence Corollary 5.5 yields

5.29 $$\begin{aligned} \begin{aligned} \#{{\mathscr {M}}}^*\left( \frac{ME^{2}}{2^{c}D}, E\right)&\ll _\varepsilon E^{2+\varepsilon } + \frac{E^{4}}{2^{c}D}+ \frac{E^{16/3}}{4^{c}D^2}, \end{aligned} \end{aligned}$$

for any $\varepsilon >0$, provided that $ME^{2}/(2^{c}D)\leqslant E^{2/3}\log E.$ The latter is equivalent to $ME^{4/3}\leqslant 2^c D\log E$, which is implied by the hypothesis of the lemma.

We shall argue differently according to the size of *c*. Let $L>0$ be a parameter to be selected in due course. For small *c*, it follows from (5.28) and (5.29) that

$$\begin{aligned} \sum _{c=0}^L\sum _{g\sim 2^{c}D}\tau _D(g)\sum _{\begin{array}{c} |\textbf{v}|\leqslant E\\ g=\gcd (Q_{1}(\textbf{v}),Q_{2}(\textbf{v})) \end{array}}1&\ll _\varepsilon \sum _{c=0}^L 2^c \left( E^{2+\varepsilon } + \frac{E^{4}}{2^{c}D}+ \frac{E^{16/3}}{4^{c}D^2}\right) \\&\ll _\varepsilon 2^L E^{2+\varepsilon } + \frac{LE^{4}}{D}+ \frac{E^{16/3}}{D^2}. \end{aligned}$$

On the other hand, for the remaining *c*, the (5.28) and (5.29) yield

$$\begin{aligned} \sum _{c>L}\sum _{g\sim 2^{c}D}\tau _D(g)\sum _{\begin{array}{c} |\textbf{v}|\leqslant E\\ g=\gcd (Q_{1}(\textbf{v}),Q_{2}(\textbf{v})) \end{array}}1&\ll _\varepsilon {\text {e}}^{\frac{2\log E}{\log \log E}} \sum _{c>L} \left( E^{2+\varepsilon } + \frac{E^{4}}{2^{c}D}+ \frac{E^{16/3}}{4^{c}D^2}\right) \\&\ll {\text {e}}^{\frac{2\log E}{\log \log E}}\left( E^{2+\varepsilon } \log E+ \frac{E^{4}}{ 2^L D}+ \frac{E^{16/3}}{4^LD^2} \right) . \end{aligned}$$

We shall take $L=\frac{4\log E}{\log \log E}$. It now follows that

$$\begin{aligned} \sum _{d\asymp D}\sum _{\begin{array}{c} |\textbf{v}|\leqslant E\\ d|Q_{i}(\textbf{v})\text { for }i=1,2 \end{array}}1\ll _\varepsilon E^{2+2\varepsilon } + \frac{E^{4}}{D}\cdot \frac{\log E}{\log \log E}+ \frac{E^{16/3}}{D^2}, \end{aligned}$$

since $c^L\log E=O_\varepsilon (E^\varepsilon )$ for any $c\geqslant 1$. Now $E^{16/3}/D^2\leqslant E^4/D$ if $E\leqslant D^{3/4}$. Hence the lemma follows on redefining the choice of $\varepsilon$. $\square$

We can use this result to assess the average size of the smallest successive minimum $s_{1,[\textbf{u}],k}$ of the lattice lattice $\Lambda _{[\textbf{u}],k}$ that was defined in (3.9), for $k\in {\mathbb {N}}$ and $[\textbf{u}]\in V_k^\times$, in the notation of (3.8). At this point it is convenient to recall the notation (3.11), where $\Delta$ is the product of bad primes defined in (2.1). The following result is rather general, since it will be used in more than one context in what follows.

#### Lemma 5.12

Let $f,e,m,D\in {\mathbb {N}}$ and assume that $m\mid e$. Then

$$\begin{aligned} \sum _{\begin{array}{c} d\asymp D\\ f|d \end{array}}\sum _{[\textbf{u}]\in V_{de}^{\times }}\frac{1}{s_{1,[\textbf{u}],\frac{de}{mf}}^{3}}\ll m_{\Delta } f_{\Delta }\cdot (mf)^{\frac{5}{4}}\cdot (De)^{-\frac{1}{4}}\frac{\log De}{\log \log De}. \end{aligned}$$

#### Proof

Let $\textbf{v}\in {\mathbb {Z}}^4$ be a non-zero vector in the lattice $\Lambda _{[\textbf{u}],\frac{de}{mf}}$, with Euclidean length equal to $s_{1,[\textbf{u}],\frac{de}{mf}}$. This implies that $\frac{de}{mf} \mid Q_i(\textbf{v})$ for $i=1,2$. Since we cannot have $Q_1(\textbf{v})=Q_2(\textbf{v})=0$, so it follows that $\frac{de}{mf}\ll |\textbf{v}|^2$. Once combined with (3.10), this therefore implies that the smallest successive minimum of $\Lambda _{[\textbf{u}],\frac{de}{mf}}$ satisfies

$$\begin{aligned} \left( \frac{de}{mf}\right) ^{\frac{1}{2}}\ll s_{1,[\textbf{u}],\frac{de}{mf}} \ll \left( \frac{de}{mf}\right) ^{\frac{3}{4}}. \end{aligned}$$

Splitting the sum in the lemma into dyadic intervals, we obtain

5.30 $$\begin{aligned} \sum _{\begin{array}{c} d\asymp D\\ f|d \end{array}}\sum _{[\textbf{u}]\in V_{de}^{\times }}\frac{1}{s_{1,[\textbf{u}],\frac{de}{mf}}^{3}}\ll \sum _{ \left( \frac{De}{mf}\right) ^{\frac{1}{2}} \swarrow E\nearrow \left( \frac{De}{mf}\right) ^{\frac{3}{4}}}\frac{S(E)}{E^{3}}, \end{aligned}$$

where

$$\begin{aligned} S(E)= \sum _{\begin{array}{c} d\asymp D\\ f|d \end{array}}\sum _{\begin{array}{c} [\textbf{u}]\in V_{de}^{\times }\\ s_{1,[\textbf{u}],\frac{de}{mf}}\sim E \end{array}}1&\leqslant \sum _{\begin{array}{c} \textbf{v}\in {\mathbb {Z}}^4\\ |\textbf{v}|\sim E \end{array}}\sum _{\begin{array}{c} d\asymp D\\ f|d \end{array}}\sum _{\begin{array}{c} [\textbf{u}]\in V_{de}^{\times }\\ \textbf{v}\in \Lambda _{[\textbf{u}],\frac{de}{mf}} \end{array}}1\\ {}&\leqslant \sum _{d'\asymp \frac{D}{f}}\sum _{\ell \mid \frac{d'e}{m}}\sum _{\begin{array}{c} |\textbf{v}|\sim E\\ \gcd (\textbf{v},\frac{d'e}{m})=\ell \\ \frac{d'e}{m}\mid Q_{i}(\textbf{v}),~i=1,2 \end{array}}\text {}\sum _{\begin{array}{c} [\textbf{u}]\in V_{d'ef}^{\times }\\ \textbf{v}\in \Lambda _{[\textbf{u}],\frac{d'e}{m}} \end{array}} 1\\&=\sum _{d'\asymp \frac{D}{f}}\sum _{\ell |\frac{d'e}{m}}\sum _{\begin{array}{c} |\textbf{v}'|\sim E/\ell \\ \gcd (\textbf{v}',\frac{d'e}{\ell m})=1\\ \frac{d'e}{m}\mid Q_{i}(\ell \cdot \textbf{v}'),~i=1,2 \end{array}}\text {}\sum _{\begin{array}{c} [\textbf{u}]\in V_{d'ef}^{\times }\\ \ell \cdot \textbf{v}'\in \Lambda _{[\textbf{u}],\frac{d'e}{m}} \end{array}}1. \end{aligned}$$

We observe that if $\ell \cdot \textbf{v}'\in \Lambda _{[\textbf{u}],\frac{d'e}{m}}$, with $\ell \mid \frac{d'e}{m}$ and $\gcd (\textbf{v}',\frac{d'e}{\ell m})=1$, then there exists $\lambda \in {\mathbb {Z}}$ such that $\ell \cdot \textbf{v}'\equiv \lambda \cdot \textbf{u}\bmod {\frac{d'e}{m}}$, which implies that $\lambda =\ell \lambda '$ for some $\lambda '\in {\mathbb {Z}}$ which is coprime with $\frac{d'e}{\ell m}$. But then it follows that $\textbf{v}'\equiv \lambda '\cdot \textbf{u}\bmod {\frac{d'e}{\ell m}}$ and so $Q_{i}(\textbf{v}')\equiv 0\bmod {\frac{d'e}{\ell m}}$, for $i=1,2$. Hence

$$\begin{aligned} S(E)\leqslant \sum _{d'\asymp \frac{D}{f}}\sum _{\ell |\frac{d'e}{m}}\sum _{\begin{array}{c} |\textbf{v}'|\sim E/\ell \\ \gcd (\textbf{v}',\frac{d'e}{\ell m})=1\\ \frac{d'e}{\ell m}\mid Q_{i}(\textbf{v}'),~i=1,2 \end{array}} \sum _{\begin{array}{c} [\textbf{u}]\in V_{d'ef}^{\times }\\ \textbf{v}'\in \Lambda _{[\textbf{u}],\frac{d'e}{\ell m}} \end{array}}1. \end{aligned}$$

The first part of Lemma 3.5 implies that the inner sum is $O( (\ell _\Delta m_{\Delta } f_{\Delta })\cdot (\ell m f))$. If write $M_{m,f}=m_{\Delta } f_{\Delta }m f$, then it follows that

$$\begin{aligned} \begin{aligned} S(E)&\ll M_{m,f} \sum _{d'\asymp \frac{D}{f}}\sum _{\ell |\frac{d'e}{m}} \sum _{\begin{array}{c} |\textbf{v}'|\sim E/\ell \\ \gcd (\textbf{v}',\frac{d'e}{\ell m})=1\\ \frac{d'e}{\ell m}\mid Q_{i}(\textbf{v}'),~i=1,2 \end{array}} \ell _{\Delta }\ell \ll M_{m,f} \sum _{\begin{array}{c} k\in {\mathbb {N}}\\ p\mid k\Rightarrow p\mid \Delta \end{array}} \sum _{\begin{array}{c} \ell \ll E\\ \ell _{\Delta }=k \end{array}}k \ell \sum _{\begin{array}{c} d'\asymp \frac{D}{f}\\ \ell |\frac{d'e}{m} \end{array}} \sum _{\begin{array}{c} |\textbf{v}'|\sim E/\ell \\ \gcd (\textbf{v}',\frac{d'e}{\ell m})=1\\ \frac{d'e}{\ell m}\mid Q_{i}( \textbf{v}'),~i=1,2 \end{array}}1. \end{aligned} \end{aligned}$$

It now follows from Lemma 5.11 that the inner sum is

$$\begin{aligned} \sum _{\begin{array}{c} d'\asymp \frac{D}{f}\\ \ell |\frac{d'e}{m} \end{array}} \sum _{\begin{array}{c} |\textbf{v}'|\sim E/\ell \\ \gcd (\textbf{v}',\frac{d'e}{\ell m})=1\\ \frac{d'e}{\ell m}\mid Q_{i}(\textbf{v}'),~i=1,2 \end{array}}1&\leqslant \sum _{\begin{array}{c} d''\asymp \frac{De}{\ell mf} \end{array}} \sum _{\begin{array}{c} |\textbf{v}'|\sim E/\ell \\ d '' \mid Q_{i}( \textbf{v}'),~i=1,2 \end{array}}1 \ll _\varepsilon \left( \frac{E}{\ell }\right) ^{2+\varepsilon }+ \frac{mfE^{4}}{\ell ^{3} De}\cdot \frac{\log E}{\log \log E}, \end{aligned}$$

for any $\varepsilon >0$, since clearly

$$\begin{aligned} \frac{E}{\ell }\ll \left( \frac{De}{\ell mf} \right) ^{3/4} \end{aligned}$$

in (5.30). Thus

$$\begin{aligned} S(E)\ll _\varepsilon M_{m,f} \left( E^{2+\varepsilon } U(1+\varepsilon ) +\frac{mf E^4}{De}\cdot \frac{\log E}{\log \log E}\cdot U(2)\right) , \end{aligned}$$

where

$$\begin{aligned} U(\theta )= \sum _{\begin{array}{c} k\in {\mathbb {N}}\\ p\mid k\Rightarrow p\mid \Delta \end{array}} \sum _{\begin{array}{c} \ell \ll E\\ \ell _{\Delta }=k \end{array}}\frac{k}{\ell ^\theta }. \end{aligned}$$

Finally, for any $\theta >1$, we note that

$$\begin{aligned} U(\theta )\leqslant \sum _{\begin{array}{c} k \ll E\\ p\mid k\Rightarrow p\mid \Delta \end{array}} \sum _{\begin{array}{c} \ell '\ll E \end{array}}\frac{k^{1-\theta }}{{\ell '}^\theta }\ll \sum _{\begin{array}{c} k \ll E\\ p\mid k\Rightarrow p\mid \Delta \end{array}} k^{1-\theta }\ll _\theta 1. \end{aligned}$$

Hence

$$\begin{aligned} S(E)\ll _\varepsilon M_{m,f} \left( E^{2+\varepsilon } +\frac{mf E^4}{De}\cdot \frac{\log E}{\log \log E}\right) . \end{aligned}$$

Returning to (5.30) and summing over dyadic intervals for *E*, the statement of the lemma easily follows. $\square$

Armed with the previous facts about our lattices $\Lambda _{[\textbf{u}],k}$, we are now ready to establish the following result, which is a critical step towards Proposition  5.6.

#### Lemma 5.13

Let $\varepsilon >0$, let $D\geqslant 1$ and let $e\in {\mathbb {N}}$. Then

$$\begin{aligned} \sum _{d\asymp D}\sum _{\begin{array}{c} \textbf{y}\in {\mathbb {Z}}_{\text {prim}}^4\\ |\textbf{y}|\leqslant Y\\ de|Q_{i}(\textbf{y}),~i=1,2 \end{array}}1\ll _{\varepsilon } \frac{Y^{4}}{De^{2-\varepsilon }}+\frac{Y^3}{(De)^{\frac{1}{4}}}\frac{\log De}{\log \log De}+D^{2}e^{1+\varepsilon }. \end{aligned}$$

#### Proof

Dropping the primitivity condition, we first note that

$$\begin{aligned} \begin{aligned} \sum _{d\asymp D}\sum _{\begin{array}{c} \textbf{y}\in {\mathbb {Z}}_{\text {prim}}^4\\ |\textbf{y}|\leqslant Y\\ de|Q_{i}(\textbf{y}),~i=1,2 \end{array}}1&\leqslant \sum _{d\asymp D}\sum _{[\textbf{u}]\in V_{de}^{\times }}\sum _{\begin{array}{c} |\textbf{y}|\leqslant Y\\ \textbf{y}\in \Lambda _{[\textbf{u}],de} \end{array}}1, \end{aligned} \end{aligned}$$

where the inner sum is now over all $\textbf{y}\in {\mathbb {Z}}^4$. Recalling that $\Lambda _{[\textbf{u}],de}$ is an integer lattice of rank 4 and determinant $(de)^3$, it follows from a lattice point counting result due to Schmidt [23, Lemma 2] that

$$\begin{aligned} \sum _{\begin{array}{c} |\textbf{y}|\leqslant Y\\ \textbf{y}\in \Lambda _{[\textbf{u}],de} \end{array}}1&\ll \frac{Y^{4}}{(de)^{3}}+\sum _{j=1}^{3}\frac{Y^{j}}{s_{1,[\textbf{u}],de}\cdots s_{j,[\textbf{u}],de}}+1 \ll \frac{Y^{4}}{(de)^{3}}+\frac{Y^{3}}{s_{1,[\textbf{u}],de}^{3}}+1, \end{aligned}$$

where $1\leqslant s_{1,[\textbf{u}],de}\leqslant \cdots \leqslant s_{4,[\textbf{u}],de}$ are the successive minima of $\Lambda _{[\textbf{u}],de}$.

Taking $f=m=1$ in Lemma 5.12, we obtain

$$\begin{aligned} \sum _{d\asymp D}\sum _{[\textbf{u}]\in V_{de}^{\times }} \frac{Y^{3}}{s_{1,[\textbf{u}],de}^{3}}\ll \frac{Y^3}{(De)^{\frac{1}{4}}}\frac{\log De}{\log \log De}. \end{aligned}$$

Moreover,

$$\begin{aligned} \begin{aligned} \sum _{d\asymp D}\sum _{[\textbf{u}]\in V_{de}^{\times }}\left( \frac{Y^{4}}{(de)^{3}}+1\right) \ll \left( Y^{4}+D^3e^{3}\right) \sum _{d\asymp D}\frac{\#V_{de}^{\times }}{(de)^{3}}. \end{aligned} \end{aligned}$$

To estimate $\#V_{de}^{\times }$ we may appeal to the second part of Lemma 3.5, which gives

$$\begin{aligned} \#V_{de}^{\times }&\ll _\varepsilon d_{\Delta }^{\varepsilon }e^{1+\varepsilon }\cdot d\cdot \prod _{\begin{array}{c} p|d\\ p\not \mid \Delta \end{array}}\left( 1+O(p^{-\frac{1}{2}})\right) , \end{aligned}$$

for any $\varepsilon >0$. Hence

$$\begin{aligned} \sum _{d\asymp D}\frac{\#V_{de}^{\times }}{(de)^{3}} \ll _\varepsilon \frac{1}{e^{2-\varepsilon }}\sum _{d\asymp D}\frac{d_{\Delta }^{\varepsilon }}{d^{2}}\cdot \prod _{\begin{array}{c} p\mid d\\ p\not \mid \Delta \end{array}}\left( 1+O(p^{-\frac{1}{2}})\right) \ll _\varepsilon \frac{1}{D e^{2-\varepsilon }}. \end{aligned}$$

The statement of the lemma follows on collecting together the various estimates. $\square$

Combing the latter with our earlier work, we can now record the following bound.

#### Corollary 5.14

Let $$D,Y\geqslant 1$$. Then

$$\begin{aligned} \sum _{d\asymp D}\sum _{\begin{array}{c} \textbf{y}\in {\mathbb {Z}}_{\text {prim}}^4\\ |\textbf{y}|\leqslant Y\\ d \mid Q_{i}(\textbf{y}),~i=1,2 \end{array}}1\ll _\varepsilon Y^{2+\varepsilon }+ \frac{Y^{4}}{D}\log YD, \end{aligned}$$

for any $$\varepsilon >0$$.

#### Proof

If $D\ll Y^{\frac{4}{3}}$, then we apply Lemma 5.13 with $e=1$. Otherwise, if $D\gg Y^{\frac{4}{3}}$, the desired bound is a consequence of Lemma 5.11. This completes the proof. $\square$

We now have all the tools in place to complete the proof of Proposition 5.6. On appealing to Lemma  5.1 and breaking the range of $|\textbf{y}|$ into dyadic intervals, we find that

$$\begin{aligned} \#{{\mathscr {M}}}(X,Y)\ll _\varepsilon X^\varepsilon Y^{2+\varepsilon } + \sum _{Y_0\nearrow Y} \sum _{\begin{array}{c} \textbf{y}\in {\mathbb {Z}}_{\text {prim}}^{4}\\ |\textbf{y}|\sim Y_0 \end{array}}M(X,\textbf{y}), \end{aligned}$$

for any $\varepsilon >0$, where $M(X,\textbf{y})=\#\left\{ \textbf{x}\in {\mathbb {Z}}_{\text {prim}}^{2}: (1.3) \text {holds}, ~|\textbf{x}|\sim X\right\} .$ Since $\gcd (x_1,x_2)=1$, (1.3) implies that $x_{1}=\pm Q_{2}(\textbf{y})/d$ and $x_{2}=\pm Q_{1}(\textbf{y})/d$, where $d=\gcd (Q_{1}(\textbf{y}),Q_{2}(\textbf{y}))$. In particular, we must have $\max (|Q_{1}(\textbf{y})|,|Q_{2}(\textbf{y})|)\sim Xd$. Let

$$\begin{aligned} C=\inf _{\textbf{t}\in {\mathbb {R}}^4, |\textbf{t}|\sim 1}\max \left( |Q_{1}(\textbf{t})|,|Q_{2}(\textbf{t})|\right) . \end{aligned}$$

Our assumption that $Z({\mathbb {R}})=\emptyset$ implies that $C>0$, for a constant *C* that depends only on the coefficients of $Q_1,Q_2.$ It follows from homogeneity that $\max (|Q_{1}(\textbf{y})|,|Q_{2}(\textbf{y})|)\asymp Y_0^2,$ for any $|\textbf{y}|\sim Y_0$. In this way we deduce that

$$\begin{aligned} \#{{\mathscr {M}}}(X,Y)\ll _\varepsilon X^\varepsilon Y^{2+\varepsilon } + \sum _{Y_0\nearrow Y} \sum _{d\asymp D} \sum _{\begin{array}{c} \textbf{y}\in {\mathbb {Z}}_{\text {prim}}^{4}\\ |\textbf{y}|\sim Y_0\\ d\mid Q_{i}(\textbf{y}),~i=1,2 \end{array}}1, \end{aligned}$$

for any $\varepsilon >0$, where $D=Y_0^{2}/X$. We now apply Lemma 5.13, which gives

$$\begin{aligned} \#{{\mathscr {M}}}(X,Y)&\ll _\varepsilon X^\varepsilon Y^{2+\varepsilon } + \sum _{Y_0\nearrow Y} \left( \frac{Y_0^{4}}{D}+\frac{Y_0^{3}}{D^{\frac{1}{4}}}\frac{\log D}{\log \log D}+D^{2}\right) \\&=X^\varepsilon Y^{2+\varepsilon } \sum _{Y_0\nearrow Y}\left( XY_0^2+ X^{\frac{1}{4}}Y_0^{\frac{5}{2}}\frac{\log Y_0}{\log \log Y_0}+\frac{Y_0^4}{X^2}\right) , \end{aligned}$$

on taking $\varepsilon =\frac{1}{4}$ and $D=Y_0^2/X$. Proposition 5.6 readily follows on summing over $Y_0$.

## Asymptotics via the geometry of numbers

The purpose of this section is to prove Proposition 2.2, which provides an asymptotic formula for

$$\begin{aligned} \#{{\mathscr {L}}}_1(B) =\#\left\{ (\textbf{x},\textbf{y})\in {{\mathscr {L}}}(B): B^{\frac{1}{4}+\eta }\leqslant |\textbf{x}|\leqslant B^{\frac{1}{2}-\eta } \right\} , \end{aligned}$$

where ${{\mathscr {L}}}(B)$ is given by (2.6). In particular, we note that any $(\textbf{x},\textbf{y})$ counted by $\#{{\mathscr {L}}}_1(B)$ must satisfy $|\textbf{y}|\leqslant B^{\frac{3}{8}-\frac{\eta }{2}}$. Breaking into dyadic intervals, we therefore find that

$$\begin{aligned} \#{{\mathscr {L}}}_1(B) = \hspace{-0.3cm}\sum _{Y\nearrow B^{\frac{3}{8}-\frac{\eta }{2}}} \hspace{-0.3cm} \# \left\{ (\textbf{x},\textbf{y})\in {{\mathbb {Z}}}_{{\text {prim}}}^2\times {{\mathbb {Z}}}_{{\text {prim}}}^4: \begin{array}{l} (1.1) \text {and} (2.3) \text {hold},~ |\textbf{y}|\sim Y\\ B^{\frac{1}{4}+\eta }\leqslant |\textbf{x}|\leqslant \min (B/|\textbf{y}|^2, B^{\frac{1}{2}-\eta }) \end{array}\right\} . \end{aligned}$$

We claim that there is a satisfactory overall contribution from *Y* in the range $Y\leqslant B^{\frac{1}{4}+\frac{\eta }{2}}.$ But it follows from Theorem 2.1 that this contribution is

$$\begin{aligned} \ll \sum _{Y\nearrow B^{\frac{1}{4}+\frac{\eta }{2}}} \left( B^{\frac{1}{2}-\eta }\cdot Y^2 +\left( \frac{B}{Y^2}\right) ^{\frac{1}{4}}Y^{\frac{5}{2}} \frac{\log B}{\log \log B}\right) \ll B, \end{aligned}$$

on breaking the sum over $\textbf{x}$ into dyadic intervals. We can use Lemma 5.1 to handle the overall contribution from those $\textbf{x},\textbf{y}$ for which (2.3) fails. Hence

6.1 $$\begin{aligned} \#{{\mathscr {L}}}_1(B) = \sum _{B^{\frac{1}{4}+\frac{\eta }{2}}\swarrow Y\nearrow B^{\frac{3}{8}-\frac{\eta }{2}}}\#{\widetilde{{\mathscr {M}}}}(Y) + O(B), \end{aligned}$$

where

6.2 $$\begin{aligned} {\widetilde{{\mathscr {M}}}}(Y)=\left\{ (\textbf{x},\textbf{y})\in {{\mathbb {Z}}}_{{\text {prim}}}^2\times {{\mathbb {Z}}}_{{\text {prim}}}^4: \begin{array}{l} (1.1)\ \text {holds},~ |\textbf{y}|\sim Y\\ B^{\frac{1}{4}+\eta }\leqslant |\textbf{x}|\leqslant B/|\textbf{y}|^2 \end{array} \right\} . \end{aligned}$$

The main goal of this section is to produce the following asymptotic formula for the cardinality of ${\widetilde{{\mathscr {M}}}}(Y)$.

### Proposition 6.1

Let $Y\geqslant 1$ such that $B^{\frac{1}{4}+\frac{\eta }{2}}\ll Y\ll B^{\frac{3}{8}-\frac{\eta }{2}}$. Then

$$\begin{aligned} \#{\widetilde{{\mathscr {M}}}}(Y)=2 \mathfrak {S}_1 B \left( \sigma _\infty (Y)+O\left( \frac{Y^2}{B^{\frac{3}{4}-\eta }}\right) \right) +O\left( \frac{B}{\sqrt{\log B}} \right) , \end{aligned}$$

where $\mathfrak {S}_1$ is given by (2.8) and

6.3 $$\begin{aligned} \sigma _\infty (Y)=\int _{\begin{array}{c} \textbf{y}\in {\mathbb {R}}^4\\ |\textbf{y}|\sim Y \end{array}} \frac{\textrm{d} \textbf{y}}{|\textbf{y}|^2\max (|Q_1(\textbf{y})|,|Q_2(\textbf{y})|)}. \end{aligned}$$

We may insert this result into (6.1), noting that $\sum _{Y\nearrow B^{\frac{3}{8}-\frac{\eta }{2}}} Y^2/B^{\frac{3}{4}-\eta }\ll 1.$ Hence we obtain

$$\begin{aligned} \#{{\mathscr {L}}}_1(B) =2\mathfrak {S}_1 B\cdot \sum _{B^{\frac{1}{4}+\frac{\eta }{2}}\swarrow Y\nearrow B^{\frac{3}{8}-\frac{\eta }{2}}} \sigma _\infty (Y) +O\left( B\sqrt{\log B} \right) . \end{aligned}$$

This therefore completes the proof of Proposition 2.2, subject to Proposition 6.1.

### Preliminary steps

We now turn to the task of estimating the cardinality of (6.2). Rewriting (1.1) as (1.3) and extracting the greatest common divisor *d* of $Q_1(\textbf{y})$ and $Q_2(\textbf{y})$, we begin our proof of Proposition 6.1 by writing

$$\begin{aligned} \#{\widetilde{{\mathscr {M}}}}(Y)= \sum _{d=1}^\infty \sum _{\begin{array}{c} \textbf{y}\in {\mathbb {Z}}_{\text {prim}}^4\cap {\mathscr {B}}(Y,d)\\ d=\gcd (Q_{1}(\textbf{y}),Q_{2}(\textbf{y})) \end{array}}2, \end{aligned}$$

where the factor 2 corresponds to the possible parameterisations $(x_1,x_2)=d^{-1}(Q_2(\textbf{y}),Q_1(\textbf{y}))$ and $(x_1,x_2)=-d^{-1}(Q_2(\textbf{y}),Q_1(\textbf{y}))$, and we have put

6.4 $$\begin{aligned} {\mathscr {B}}(Y,d)=\left\{ \textbf{y}\in \mathbb {{\mathbb {R}}}^{4}: |\textbf{y}|\sim Y,~ B^{\frac{1}{4}+\eta }\leqslant \frac{\max (|Q_{1}(\textbf{y})|,|Q_2(\textbf{y})|)}{d}\leqslant \frac{B}{|\textbf{y}|^2}\right\} . \end{aligned}$$

The assumption $Z({\mathbb {R}})=\emptyset$ implies that $\max (|Q_{1}(\textbf{y})|,|Q_2(\textbf{y})|)\asymp Y^2$ for any $\textbf{y}\in {\mathbb {R}}^4$ such that $|\textbf{y}|\sim Y$. Hence $D_1\ll d\ll D_2$ where

6.5 $$\begin{aligned} D_1=\frac{Y^4}{B} \quad \text { and }\quad D_2=\frac{Y^2}{B^{\frac{1}{4}+\eta }}. \end{aligned}$$

It follows that

$$\begin{aligned} \#{\widetilde{{\mathscr {M}}}}(Y)= \sum _{D_1\ll d\ll D_2}\sum _{\begin{array}{c} \textbf{y}\in {\mathbb {Z}}_{\text {prim}}^4\cap {\mathscr {B}}(Y,d)\\ d=\gcd (Q_{1}(\textbf{y}),Q_{2}(\textbf{y})) \end{array}}2. \end{aligned}$$

Let $M,z>0$ be parameters to be chosen in due course. Let

6.6 $$\begin{aligned} T_{1}= \sum _{D_1\ll d\ll D_2}\sum _{\begin{array}{c} \textbf{y}\in {\mathbb {Z}}_{\text {prim}}^4\cap {\mathscr {B}}(Y,d)\\ d\mid Q_i(\textbf{y}),~i=1,2\\ \gcd (d^{-1}Q_{1}(\textbf{y}),d^{-1}Q_{2}(\textbf{y}),P(z))=1 \end{array}}1. \end{aligned}$$

Then we clearly have

6.7 $$\begin{aligned} 2T_{1}-T_{2}-T_{3}\leqslant \#\widetilde{{\mathscr {M}}}(X,Y)\leqslant 2T_{1}, \end{aligned}$$

where

$$\begin{aligned} T_{2}&= \sum _{D_1\ll d\ll D_2}\sum _{\begin{array}{c} \textbf{y}\in {\mathbb {Z}}_{\text {prim}}^4\cap {\mathscr {B}}(Y,d)\\ d\mid Q_i(\textbf{y}),~i=1,2\\ \exists p\in (z,M] \text { s.t. } p\mid \frac{Q_{i}(\textbf{y})}{d},~i=1,2 \end{array}}1, \qquad T_{3}= \sum _{D_1\ll d\ll D_2}\sum _{\begin{array}{c} \textbf{y}\in {\mathbb {Z}}_{\text {prim}}^4\cap {\mathscr {B}}(Y,d)\\ d\mid Q_i(\textbf{y}),~i=1,2\\ \exists p>M \text { s.t. } p\mid \frac{Q_{i}(\textbf{y})}{d},~i=1,2 \end{array}}1. \end{aligned}$$

We shall produce upper bounds for $T_2$ and $T_3$, and an asymptotic formula for $T_1$.

#### Lemma 6.2

Let $$\varepsilon >0$$. Then

$$\begin{aligned} T_{2}&\ll _\eta \frac{B}{z^{1-\varepsilon }}+ B^{1-\frac{\eta }{2}}M^3 \quad \text { and }\quad T_{3} \ll _\varepsilon Y^{2+\varepsilon }+ \frac{B(\log B)^{2}}{M}. \end{aligned}$$

#### Proof

We start by noting that $T_{2}\leqslant \sum _{p\in (z,M]}U_{p}$, where

$$\begin{aligned} U_{p}= \sum _{D_1\ll d\ll D_2}\sum _{\begin{array}{c} \textbf{y}\in {\mathbb {Z}}_{\text {prim}}^4\\ |\textbf{y}|\sim Y\\ dp\mid Q_{i}(\textbf{y}),~i=1,2 \end{array}}1, \end{aligned}$$

for any prime *p*. We may use Lemma 5.13 to estimate $U_p$, on breaking the *d*-sum into dyadic intervals. In this way, on recalling (6.5), we see that

$$\begin{aligned} T_2&\ll _{\varepsilon } \sum _{p\in (z,M]}\left( \frac{Y^{4}}{D_1p^{2-\varepsilon }}+\frac{Y^{3}}{(D_1p)^{\frac{1}{4}}}\frac{\log D_2p}{\log \log D_2p}+D_2^{2}p^{1+\varepsilon }\right) \\&\ll \frac{Y^{4}}{D_1z^{1-\varepsilon }} +\frac{Y^{3}M^{\frac{3}{4}}\log B}{D_1^{\frac{1}{4}}}+D_2^{2}M^{2+\varepsilon }\\&\ll \frac{B}{z^{1-\varepsilon }}+B^{\frac{1}{4}}Y^{2}M^{\frac{3}{4}}\log B+\frac{Y^{4}M^{2+\varepsilon }}{B^{\frac{1}{2}+2\eta }}, \end{aligned}$$

for any $\varepsilon >0$. Since $Y\ll B^{\frac{3}{8}-\frac{\eta }{2}}$, it follows that

$$\begin{aligned} B^{\frac{1}{4}}Y^{2}M^{\frac{3}{4}}\log B\ll B^{1-\eta }M^{\frac{3}{4}}\log B\ll _\eta B^{1-\frac{\eta }{2}}M^3. \end{aligned}$$

Similarly,

$$\begin{aligned} \frac{Y^{4}M^{2+\varepsilon }}{B^{\frac{1}{2}+2\eta }} \ll B^{1-4\eta } M^{2+\varepsilon }\ll B^{1-\frac{\eta }{2}}M^3, \end{aligned}$$

which completes the proof of the first part of the lemma.

We now turn to $T_{3} \leqslant \sum _{p>M}U_{p}.$ If $pd\mid Q_i(\textbf{y})$ for $i=1,2$ then there exists $q\gg D_1M$ such that $q\mid Q_{i}(\textbf{y})$ for $i=1,2$. Under our assumption $Z({\mathbb {R}})=\emptyset$, it follows that

$$\begin{aligned} T_{3}\ll \log Y \sum _{D_1M\ll q\ll Y^{2}} \sum _{\begin{array}{c} \textbf{y}\in {\mathbb {Z}}_{\text {prim}}^4\\ |\textbf{y}|\sim Y\\ q\mid Q_{i}(\textbf{y}),~i=1,2 \end{array}}1, \end{aligned}$$

since there $O(\log Y)$ primes divisors of *q*. Splitting the range of summation over *q* into dyadic intervals, Corollary 5.14 yields

$$\begin{aligned} T_3&\ll \log Y\sum _{D_1M \swarrow G\nearrow Y^{2}} \left( Y^{2+\frac{\varepsilon }{2}}+\frac{Y^{4}}{G}\log B\right) \ll _\varepsilon Y^{2+\varepsilon }+ \frac{Y^{4}}{D_1M}(\log B)^2. \end{aligned}$$

Recalling (6.5), this is also satisfactory for the lemma. $\square$

### Asymptotic formula for $T_{1}$

It remains to deal with the sum (6.6). The first step is to reduce the primitivity condition on $\textbf{y}$ to the requirement that $\gcd (\textbf{y},dP(z))=1$, where $P(z)=\prod _{p\leqslant z}p$. This is the purpose of the following result.

#### Lemma 6.3

Let $$\varepsilon >0$$. Then

$$\begin{aligned} T_{1}=\Sigma _1 + O_\varepsilon \left( Y^{2+\varepsilon }+\frac{B\log B}{z^{3}}\right) , \end{aligned}$$

where

$$\begin{aligned} \Sigma _1= \sum _{D_1\ll d\ll D_2}\sum _{\begin{array}{c} \textbf{y}\in {\mathbb {Z}}^4\cap {\mathscr {B}}(Y,d)\\ \gcd (\textbf{y},dP(z))=1\\ d\mid Q_i(\textbf{y}),~i=1,2\\ \gcd (d^{-1}Q_{1}(\textbf{y}),d^{-1}Q_{2}(\textbf{y}),P(z))=1 \end{array}} \hspace{-0.5cm} 1. \end{aligned}$$

#### Proof

The proof hinges on the observation that

$$\begin{aligned} T_1- \sum _{D_1\ll d\ll D_2}\sum _{\begin{array}{c} \textbf{y}\in {\mathbb {Z}}^4\cap {\mathscr {B}}(Y,d)\\ \gcd (\textbf{y},dP(z))=1\\ d\mid Q_i(\textbf{y}),~i=1,2\\ \gcd (d^{-1}Q_{1}(\textbf{y}),d^{-1}Q_{2}(\textbf{y}),P(z))=1 \end{array}} \hspace{-0.5cm} 1\leqslant \sum _{\begin{array}{c} k>1\\ \gcd (k,P(z))=1 \end{array}} R_k, \end{aligned}$$

where now

$$\begin{aligned} R_k=\sum _{\begin{array}{c} D_1\ll d\ll D_2\\ \gcd (d,k)=1 \end{array}}\sum _{\begin{array}{c} \textbf{y}\in {\mathbb {Z}}^4\cap {\mathscr {B}}(Y,d)\\ k=\gcd (y_1,\dots ,y_4)\\ d\mid Q_i(\textbf{y}),~i=1,2\\ \gcd (d^{-1}Q_{1}(\textbf{y}),d^{-1}Q_{2}(\textbf{y}),P(z))=1 \end{array}} \hspace{-0.5cm} 1. \end{aligned}$$

But clearly

$$\begin{aligned} R_k&\leqslant \sum _{\begin{array}{c} D_1\ll d\ll D_2\\ \gcd (d,k)=1 \end{array}} \sum _{\begin{array}{c} \textbf{y}'\in {\mathbb {Z}}_{\text {prim}}^4\cap k^{-1}{\mathscr {B}}(Y,d)\\ d\mid Q_i(\textbf{y}'),~i=1,2\\ \gcd (d^{-1}Q_{1}(\textbf{y}'),d^{-1}Q_{2}(\textbf{y}'),P(z))=1 \end{array}} \hspace{-0.5cm} 1 \leqslant \sum _{\begin{array}{c} D_1\ll d\ll D_2 \end{array}}\sum _{\begin{array}{c} \textbf{y}'\in {\mathbb {Z}}_{\text {prim}}^4\\ |\textbf{y}'|\sim Y/k\\ d\mid Q_i(\textbf{y}'),~i=1,2 \end{array}} \hspace{-0.5cm} 1. \end{aligned}$$

Breaking the *d*-sum into dyadic intervals, it follows from Corollary 5.14 and (6.5) that

$$\begin{aligned} R_k\ll _\varepsilon \left( \frac{Y}{k}\right) ^{2+\varepsilon } +\frac{(Y/k)^4\log Y}{D_1}\ll \frac{Y^{2+\varepsilon }}{k^2} + \frac{B \log B}{k^4}, \end{aligned}$$

for any $\varepsilon >0$. Hence

$$\begin{aligned} \sum _{\begin{array}{c} k>1\\ \gcd (k,P(z))=1 \end{array}} R_k\ll _\varepsilon Y^{2+\varepsilon } + B\log B\sum _{k>z} \frac{1}{k^4}. \end{aligned}$$

The remaining sum is $O(z^{-3})$, which thereby completes the proof of the lemma. $\square$

We now turn our attention to an asymptotic evaluation of the main term $\Sigma _1$ in the previous lemma. We use the geometry of numbers to prove the following result, in which we recall the notation (3.8) for $V_{de}^\times$.

#### Lemma 6.4

We have

$$\begin{aligned} \Sigma _1= \sum _{\begin{array}{c} \ell _{1}\mid P(z)\\ \ell _{2}\mid P(z)\\ \ell _3\ll D_2 \end{array}}\frac{\mu (\ell _{1}\ell _2\ell _{3})}{\ell _1^4\ell _{2}\ell _{3}} \hspace{-0.3cm} \sum _{\begin{array}{c} e\mid \frac{P(z)}{\ell _{1}}\\ \ell _{2}\mid e\\ \gcd (e,\ell _{3})=1 \end{array}} \hspace{-0.3cm} \frac{\mu (e)}{e^3} S_{\ell _1,\ell _3,e}+O_\eta \left( \left( 2^z +P(z)^4\right) B^{1-\frac{\eta }{2}}\right) , \end{aligned}$$

where

$$\begin{aligned} S_{\ell _1,\ell _3,e}= \sum _{\begin{array}{c} D_1\ll d\ll D_2\\ \gcd (d,\ell _{1})=1\\ \ell _{3}\mid d \end{array}} \frac{ \# V_{de}^{\times } \cdot {{\,\textrm{vol}\,}}{\mathscr {B}}(Y,d)}{d^3}. \end{aligned}$$

#### Proof

Using Möbius inversion to handle $\gcd (d^{-1}Q_{1}(\textbf{y}),d^{-1}Q_{2}(\textbf{y}),P(z))=1$, we see that

$$\begin{aligned} \Sigma _1&= \sum _{e\mid P(z)} \mu (e)\sum _{D_1\ll d\ll D_2}\sum _{\begin{array}{c} \textbf{y}\in {\mathbb {Z}}^4\cap {\mathscr {B}}(Y,d)\\ \gcd (\textbf{y},dP(z))=1\\ de\mid Q_i(\textbf{y}),~i=1,2 \end{array}} 1 =\sum _{e\mid P(z)}\mu (e)\sum _{\begin{array}{c} D_1\ll d\ll D_2 \end{array}}\sum _{[\textbf{u}]\in V_{de}^{\times }}\sum _{\begin{array}{c} \textbf{y}\in {\mathbb {Z}}^4\cap {\mathscr {B}}(Y,d)\\ \gcd (\textbf{y},dP(z))=1\\ \textbf{y}\in \Lambda _{[\textbf{u}],de} \end{array}}1, \end{aligned}$$

where $V_{de}^{\times }$ and $\Lambda _{[\textbf{u}],de}$ are given by (3.8) and (3.9), respectively. Appealing to Möbius inversion once more, we obtain

$$\begin{aligned} \Sigma _{1}=\sum _{e\mid P(z)}\mu (e)\sum _{\begin{array}{c} D_1\ll d\ll D_2 \end{array}}\sum _{[\textbf{u}]\in V_{de}^{\times }}\sum _{\begin{array}{c} \textbf{y}\in {\mathbb {Z}}^4\cap {\mathscr {B}}(Y,d)\\ \textbf{y}\in \Lambda _{[\textbf{u}],de} \end{array}}\sum _{\begin{array}{c} \ell \mid \textbf{y}\\ \ell \mid dP(z) \end{array}}\mu (\ell ). \end{aligned}$$

It will be convenient to observe that

$$\begin{aligned} \begin{aligned} \sum _{\begin{array}{c} \ell \mid \textbf{y}\\ \ell \mid dP(z) \end{array}}\mu (\ell )&=\sum _{\begin{array}{c} c\mid \textbf{y}\\ c\mid de \end{array}}\sum _{\begin{array}{c} \ell _{1}\mid \textbf{y}\\ \ell _{1}\mid P(z)\\ \gcd (\ell _{1},de)=1 \end{array}}\mu (\ell _{1})\mu (c) =\sum _{\begin{array}{c} \ell _{1}\mid \textbf{y}\\ \ell _{1}\mid P(z)\\ \gcd (\ell _{1},de)=1 \end{array}}\sum _{\begin{array}{c} \ell _{2}\mid \textbf{y}\\ \ell _{2}\mid e \end{array}}\sum _{\begin{array}{c} \ell _{3}\mid \textbf{y}\\ \ell _{3}\mid d\\ \gcd (\ell _{3},e)=1 \end{array}}\mu (\ell _{1})\mu (\ell _{2})\mu (\ell _{3}). \end{aligned} \end{aligned}$$

Note that $\mu (\ell _{1})\mu (\ell _{2})\mu (\ell _{3})=\mu (\ell _{1}\ell _2\ell _{3})$ in the summand. But then it follows that

$$\begin{aligned} \begin{aligned} \Sigma _{1}&=\sum _{\begin{array}{c} \ell _{1}\mid P(z)\\ \ell _{2}\mid P(z)\\ \ell _3\ll D_2 \end{array}}\mu (\ell _{1}\ell _2\ell _{3}) \sum _{\begin{array}{c} e\mid \frac{P(z)}{\ell _{1}}\\ \ell _{2}\mid e\\ \gcd (e,\ell _{3})=1 \end{array}}\mu (e)\sum _{\begin{array}{c} D_1\ll d\ll D_2\\ \gcd (d,\ell _{1})=1\\ \ell _{3}\mid d \end{array}} \sum _{[\textbf{u}]\in V_{de}^{\times }}\sum _{\begin{array}{c} \textbf{y}\in {\mathbb {Z}}^4\cap {\mathscr {B}}(Y,d)\\ \ell _{1}\ell _{2}\ell _{3}\mid \textbf{y}\\ \textbf{y}\in \Lambda _{[\textbf{u}],de} \end{array}}1\\&=\sum _{\begin{array}{c} \ell _{1}\mid P(z)\\ \ell _{2}\mid P(z)\\ \ell _3\ll D_2 \end{array}}\mu (\ell _{1}\ell _2\ell _{3}) \sum _{\begin{array}{c} e\mid \frac{P(z)}{\ell _{1}}\\ \ell _{2}\mid e\\ \gcd (e,\ell _{3})=1 \end{array}}\mu (e)\sum _{\begin{array}{c} D_1\ll d\ll D_2\\ \gcd (d,\ell _{1})=1\\ \ell _{3}\mid d \end{array}} \sum _{[\textbf{u}]\in V_{de}^{\times }} N, \end{aligned} \end{aligned}$$

where

$$\begin{aligned} N= \#\left( {\mathbb {Z}}^4\cap (\ell _1\ell _2\ell _3)^{-1}{\mathscr {B}}(Y,d)\cap \Lambda _{[\textbf{u}],\frac{de}{\ell _2\ell _3}}\right) . \end{aligned}$$

Recalling the definition (6.4) of ${\mathscr {B}}(Y,d)$, we now appeal to the lattice point counting result worked out by Schmidt [23, Lemma 2]. This yields

$$\begin{aligned} N= \frac{{{\,\textrm{vol}\,}}\left( (\ell _1\ell _2\ell _3)^{-1}{\mathscr {B}}\left( Y,d\right) \right) \cdot (\ell _{2}\ell _{3})^{3}}{(de)^{3}}+O\left( 1+\frac{Y^{3}}{(\ell _{1}\ell _{2} \ell _{3})^{3}s_{1,[\textbf{u}],\frac{de}{\ell _{2}\ell _{3}}}^{3}}\right) , \end{aligned}$$

where $s_{1,[\textbf{u}],\frac{de}{\ell _{2}\ell _{3}}}$ is the smallest successive minimum of the lattice $\Lambda _{[\textbf{u}],\frac{de}{\ell _2\ell _3}}$.

Since ${{\,\textrm{vol}\,}}\left( (\ell _1\ell _2\ell _3)^{-1}{\mathscr {B}}\left( Y,d\right) \right) = (\ell _1\ell _2\ell _3)^{-4}{{\,\textrm{vol}\,}}{\mathscr {B}}(Y,d),$ it follows that

6.8 $$\begin{aligned} N= \frac{{{\,\textrm{vol}\,}}{\mathscr {B}}(Y,d)}{(de)^{3}\ell _1^4\ell _{2}\ell _{3}} +O\left( 1+\frac{Y^{3}}{(\ell _{1}\ell _{2}\ell _{3})^{3}s_{1,[\textbf{u}],\frac{de}{\ell _{2}\ell _{3}}}^{3}}\right) . \end{aligned}$$

We begin by handling the overall contribution to $\Sigma _1$ from the error terms. Let $E_1$ denote the overall contribution from the term *O*(1) and let $E_2$ denote the contribution from the term involving the first successive minimum. Beginning with the latter, it follows from Lemma 5.12 that

$$\begin{aligned} \sum _{\begin{array}{c} D_1\ll d\ll D_2\\ \ell _{3}\mid d \end{array}}\sum _{[\textbf{u}]\in V_{de}^{\times }}\frac{Y^{3}}{(\ell _{1}\ell _{2}\ell _{3})^{3}s_{1,[\textbf{u}],\frac{de}{\ell _{2}\ell _{3}}}^{3}} \ll \frac{Y^{3}}{\ell _{1}^{3}(\ell _{2}\ell _{3})^{\frac{7}{4}}}\cdot (\ell _{2,\Delta }\ell _{3,\Delta }) \cdot (D_1e)^{-\frac{1}{4}}\frac{\log B}{\log \log B}, \end{aligned}$$

making the overall contribution

$$\begin{aligned} E_2\ll B^{1-\eta }\frac{\log B}{\log \log B}\sum _{\ell _{2}\mid P(z)}\frac{\ell _{2,\Delta }}{\ell _{2}^{\frac{7}{4}}} \sum _{\ell _{3}\ll D_2}\frac{\ell _{3,\Delta }}{\ell _{3}^{\frac{7}{4}}} \sum _{\begin{array}{c} e\mid P(z)\\ \ell _{2}\mid e \end{array}}e^{-\frac{1}{4}}, \end{aligned}$$

since $D_1$ satisfies (6.5) and $Y\ll B^{\frac{3}{8}-\frac{\eta }{2}}$. The inner sum is at most $2^z$ and the sums over $\ell _2,\ell _3$ are *O*(1), since

$$\begin{aligned} \sum _{\ell \in {\mathbb {N}}}\frac{\ell _{\Delta }}{\ell ^{\frac{7}{4}}}\leqslant \sum _{\begin{array}{c} k\in {\mathbb {N}}\\ p\mid k\Rightarrow p\mid \Delta \end{array}} \frac{1}{k^{\frac{3}{4}}} \sum _{\ell '\in {\mathbb {N}}} \frac{1}{{\ell '}^{\frac{7}{4}}}\ll 1. \end{aligned}$$

Hence $E_2=O_\eta (2^z B^{1-\frac{\eta }{2}})$, which is satisfactory.

The remaining error term in the lattice point counting result makes the contribution

6.9 $$\begin{aligned} E_1\ll \sum _{\begin{array}{c} \ell _{1}\mid P(z)\\ \ell _{2}\mid P(z)\\ \ell _3\ll D_2 \end{array}}|\mu (\ell _{1}\ell _2\ell _{3})| \sum _{\begin{array}{c} e\mid P(z)\\ \ell _{2}\mid e \end{array}}\sum _{\begin{array}{c} D_1\ll d\ll D_2 \\ \ell _{3}\mid d \end{array}} \#V_{de}^{\times } \end{aligned}$$

to $\Sigma _1$. We claim that

6.10 $$\begin{aligned} \sum _{\begin{array}{c} e\mid P(z)\\ \ell _{2}\mid e \end{array}}\sum _{\begin{array}{c} d\ll D_2 \\ \ell _{3}\mid d \end{array}} \#V_{de}^{\times } \ll P(z)^2D_2^2 \frac{(\ell _{2,\Delta }\ell _{3,\Delta })^\varepsilon }{\ell _2\ell _3} \prod _{\begin{array}{c} p\mid \ell _2\ell _3\\ p\not \mid \Delta \end{array}} \left( 1+O(p^{-\frac{1}{2}})\right) . \end{aligned}$$

To prove this, we appeal to the second part of Lemma 3.5, which implies that

$$\begin{aligned} \sum _{\begin{array}{c} d\leqslant D \\ \ell _{3}\mid d \end{array}} \#V_{de}^{\times }&\ll _\varepsilon \sum _{\begin{array}{c} d\leqslant D \\ \ell _{3}\mid d \end{array}} de d_\Delta ^\varepsilon e_\Delta ^\varepsilon \prod _{\begin{array}{c} p\mid de\\ p\not \mid \Delta \end{array}} \left( 1+O(p^{-\frac{1}{2}})\right) \\&\ll e e_{\Delta }^\varepsilon \sum _{\begin{array}{c} d'\leqslant D/\ell _3 \end{array}} \ell _3d' (\ell _{3,\Delta }d_\Delta ')^\varepsilon \prod _{\begin{array}{c} p\mid \ell _3d'e\\ p\not \mid \Delta \end{array}} \left( 1+O(p^{-\frac{1}{2}})\right) , \end{aligned}$$

for any $D\geqslant 1$, on writing $d=\ell _{3}d'$. Now

$$\begin{aligned} \sum _{\begin{array}{c} d'\leqslant D/\ell _3 \end{array}}d'd_\Delta '^\varepsilon \prod _{\begin{array}{c} p\mid d'\\ p\not \mid \Delta \end{array}} \left( 1+O(p^{-\frac{1}{2}})\right)&\ll \sum _{\begin{array}{c} k\in {\mathbb {N}}\\ p\mid k\Rightarrow p\mid \Delta \end{array}} k^{1+\varepsilon } \sum _{d''\leqslant D/(k\ell _3)} d'' \prod _{\begin{array}{c} p\mid d'' \end{array}} \left( 1+O(p^{-\frac{1}{2}})\right) \\&\ll \frac{D^2}{\ell _3^2} \sum _{\begin{array}{c} k\in {\mathbb {N}}\\ p\mid k\Rightarrow p\mid \Delta \end{array}} k^{-1+\varepsilon }. \end{aligned}$$

This is $O_\varepsilon ({D^2}/{\ell _3^2})$. Hence we have proved that

6.11 $$\begin{aligned} \sum _{\begin{array}{c} d\leqslant D \\ \ell _{3}\mid d \end{array}} \#V_{de}^{\times } \ll \frac{e e_{\Delta }^\varepsilon \ell _{3,\Delta }^\varepsilon D^2}{\ell _3} \prod _{\begin{array}{c} p\mid \ell _3 e\\ p\not \mid \Delta \end{array}} \left( 1+O(p^{-\frac{1}{2}})\right) , \end{aligned}$$

for any $D\geqslant 1$. Making the change of variable $e=\ell _{2}e'$, and noting that

$$\begin{aligned} \sum _{\begin{array}{c} e'\mid \frac{P(z)}{\ell _{2}} \end{array}}e'e_\Delta '^{\varepsilon } \prod _{\begin{array}{c} p\mid e'\\ p\not \mid \Delta \end{array}} \left( 1+O(p^{-\frac{1}{2}})\right) \ll \frac{P(z)^{2}}{\ell _{2}^{2}}, \end{aligned}$$

the claimed bound (6.10) readily follow.

It follows from (6.5) and $Y\ll B^{\frac{3}{8}-\frac{\eta }{2}}$ that $D_2^2\ll B^{1-4\eta }$. On inserting (6.10) into (6.9) and summing trivially over $\ell _1$, a similar analysis leads to the conclusion that

$$\begin{aligned} E_1\ll _\varepsilon P(z)^3D_2^2 \sum _{\ell _{2}\mid P(z)}\sum _{\ell _3\ll D_2} \frac{(\ell _{2,\Delta }\ell _{3,\Delta })^\varepsilon }{\ell _2\ell _3} \prod _{\begin{array}{c} p\mid \ell _2\ell _3\\ p\not \mid \Delta \end{array}} \left( 1+O(p^{-\frac{1}{2}})\right) \\ \ll _\eta P(z)^4B^{1-\frac{\eta }{2}}, \end{aligned}$$

which is satisfactory for the lemma.

Finally, we note that the main term in our asymptotic formula (6.8) for *N* gives

$$\begin{aligned} \sum _{\begin{array}{c} \ell _{1}\mid P(z)\\ \ell _{2}\mid P(z)\\ \ell _3\ll D_2 \end{array}}\mu (\ell _{1}\ell _2\ell _{3}) \sum _{\begin{array}{c} e\mid \frac{P(z)}{\ell _{1}}\\ \ell _{2}\mid e\\ \gcd (e,\ell _{3})=1 \end{array}}\mu (e) \sum _{\begin{array}{c} D_1\ll d\ll D_2\\ \gcd (d,\ell _{1})=1\\ \ell _{3}\mid d \end{array}} \sum _{[\textbf{u}]\in V_{de}^{\times }} \frac{{{\,\textrm{vol}\,}}{\mathscr {B}}(Y,d)}{(de)^{3}\ell _1^4\ell _{2}\ell _{3}}, \end{aligned}$$

once inserted into our expression for $\Sigma _1$. Finally, on rearranging the terms, we are led to the main term in the lemma. $\square$

We now have everything in place to complete the first step in the proof of Proposition 6.1. We shall take

$$\begin{aligned} z=\frac{\log B}{(\log \log B)^{2}} \quad \text { and }\quad M=B^{\frac{\eta }{10}}. \end{aligned}$$

Mertens’ theorem implies that $P(z)\ll {\text {e}}^{\frac{\log B}{(\log \log B)^2}}\ll _\eta B^{\frac{\eta }{16}}$. Hence we obtain

$$\begin{aligned} \Sigma _1= \hspace{-0.1cm} \sum _{\begin{array}{c} \ell _{1}\mid P(z)\\ \ell _{2}\mid P(z)\\ \ell _3\ll D \end{array}}\frac{\mu (\ell _{1}\ell _2\ell _{3})}{\ell _1^4\ell _{2}\ell _{3}} \hspace{-0.3cm} \sum _{\begin{array}{c} e\mid \frac{P(z)}{\ell _{1}}\\ \ell _{2}\mid e\\ \gcd (e,\ell _{3})=1 \end{array}} \hspace{-0.3cm} \frac{\mu (e)}{e^3} S_{\ell _1,\ell _3,e}+ O_\eta \left( B^{1-\frac{\eta }{4}}\right) , \end{aligned}$$

in Lemma 6.4. Next, it follows from Lemma  6.2 that

$$\begin{aligned} T_{2}+T_3&\ll _\varepsilon \frac{B}{z^{1-\varepsilon }}+ B^{1-\frac{\eta }{2}}M^3+ Y^{2+\varepsilon }+ \frac{B(\log B)^{2}}{M} \ll \frac{B}{\sqrt{\log B}}. \end{aligned}$$

since $Y\ll B^{\frac{3}{8}-\frac{\eta }{2}}$. Inserting these estimates into (6.7), we obtain

$$\begin{aligned} \#{\widetilde{{\mathscr {M}}}}(Y)=2 \sum _{\begin{array}{c} \ell _{1}\mid P(z)\\ \ell _{2}\mid P(z)\\ \ell _3\ll D_2 \end{array}}\frac{\mu (\ell _{1}\ell _2\ell _{3})}{\ell _1^4\ell _{2}\ell _{3}} \sum _{\begin{array}{c} e\mid \frac{P(z)}{\ell _{1}}\\ \ell _{2}\mid e\\ \gcd (e,\ell _{3})=1 \end{array}} \frac{\mu (e)}{e^3} S_{\ell _1,\ell _3,e} +\left( \frac{B}{\sqrt{\log B}}\right) . \end{aligned}$$

We next show that the sum over $\ell _3$ can be truncated with acceptable error, as in the following result.

#### Lemma 6.5

We have

$$\begin{aligned} \#{\widetilde{{\mathscr {M}}}}(Y)=2T_0+\left( \frac{B}{\sqrt{\log B}}\right) , \end{aligned}$$

where

$$\begin{aligned} T_0=\sum _{\begin{array}{c} \ell _{1}\mid P(z)\\ \ell _{2}\mid P(z)\\ \ell _3\mid P(z) \end{array}}\frac{\mu (\ell _{1}\ell _2\ell _{3})}{\ell _1^4\ell _{2}\ell _{3}} \hspace{-0.3cm} \sum _{\begin{array}{c} e\mid \frac{P(z)}{\ell _{1}}\\ \ell _{2}\mid e\\ \gcd (e,\ell _{3})=1 \end{array}} \hspace{-0.3cm} \frac{\mu (e)}{e^3} \sum _{\begin{array}{c} D_1\ll d\ll D_2\\ \gcd (d,\ell _{1})=1\\ \ell _{3}\mid d \end{array}} \frac{ \# V_{de}^{\times } \cdot {{\,\textrm{vol}\,}}{\mathscr {B}}(Y,d)}{d^3}. \end{aligned}$$

#### Proof

Recall the definition of $S_{\ell _1,\ell _3,e}$ from the statement of Lemma 6.4. Then, in order to prove the lemma, it will suffice to bound

$$\begin{aligned} E&= \sum _{\begin{array}{c} \ell _{1}\mid P(z)\\ \ell _{2}\mid P(z) \end{array}} \sum _{\begin{array}{c} \ell _3\ll D_2\\ \ell _3\not \mid P(z) \end{array}} \frac{|\mu (\ell _{1}\ell _2\ell _{3})|}{\ell _1^4\ell _{2}\ell _{3}} \sum _{\begin{array}{c} e\mid P(z)\\ \ell _{2}\mid e \end{array}} \frac{|\mu (e)|}{e^3} S_{\ell _1,\ell _3,e}\\&\ll Y^4 \sum _{\begin{array}{c} \ell _{1}\mid P(z)\\ \ell _{2}\mid P(z) \end{array}} \sum _{\begin{array}{c} \ell _3\ll D_2\\ \ell _3\not \mid P(z) \end{array}} \frac{1}{\ell _1^4\ell _{2}\ell _{3}} \sum _{\begin{array}{c} e\mid P(z)\\ \ell _{2}\mid e \end{array}} \frac{1}{e^3} \sum _{\begin{array}{c} D_1\ll d\ll D_2\\ \ell _{3}\mid d \end{array}} \frac{\# V_{de}^{\times }}{d^3}. \end{aligned}$$

A modest reworking of the proof of (6.10), using partial summation and (6.11) to incorporate the weight $(de)^{-3}$, easily yields

$$\begin{aligned} E\ll _\varepsilon \frac{Y^4}{D_1 } \sum _{\begin{array}{c} \ell _{1}\mid P(z)\\ \ell _{2}\mid P(z) \end{array}} \sum _{\begin{array}{c} \ell _3\ll D_2\\ \ell _3\not \mid P(z) \end{array}} \frac{1}{\ell _1^4\ell _{2}^{3-\varepsilon }\ell _{3}^{2-\varepsilon }}\ll _\varepsilon \frac{Y^4}{D_1 } \sum _{\begin{array}{c} \ell _3>z \end{array}} \frac{1}{\ell _{3}^{2-\varepsilon }}\ll \frac{B}{z^{1-\varepsilon }}, \end{aligned}$$

by (6.5). Our choice of *z* ensures this is satisfactory. $\square$

### Asymptotic formula for $T_{0}$

The final step in the proof of Proposition 6.1 is to analyse the main term in Lemma 6.5, in which we recall that ${\mathscr {B}}(Y,d)$ is given by (6.4) and the piece of notation (3.8). Making the change of variables $e=\ell _2e'$ and $d=\ell _3d'$, we find that

6.12 $$\begin{aligned} T_0=\sum _{\begin{array}{c} \ell _{1}\mid P(z)\\ \ell _{2}\mid P(z)\\ \ell _3\mid P(z) \end{array}}\frac{\mu (\ell _{1}\ell _2\ell _{3})\mu (\ell _2)}{\ell _1^4\ell _{2}^4\ell _{3}^4} \hspace{-0.3cm} \sum _{\begin{array}{c} e'\mid \frac{P(z)}{\ell _{1}\ell _2}\\ \gcd (e',\ell _{3})=1 \end{array}} \hspace{-0.3cm} \frac{\mu (e')}{e'^3}Q_{\ell _1,\ell _2,\ell _3,e'}, \end{aligned}$$

where

6.13 $$\begin{aligned} \begin{aligned} Q_{\ell _1,\ell _2,\ell _3,e'}&= \sum _{\begin{array}{c} D_1/\ell _3\ll d'\ll D_2/\ell _3\\ \gcd (d',\ell _{1})=1 \end{array}} \frac{ \# V_{\ell _2\ell _3 d'e'}^{\times } \cdot {{\,\textrm{vol}\,}}{\mathscr {B}}(Y,\ell _3 d')}{d'^3}\\&=\int _{\begin{array}{c} \textbf{y}\in {\mathbb {R}}^4\\ |\textbf{y}|\sim Y \end{array}} \sum _{\begin{array}{c} D_{1,\textbf{y}}\leqslant d' \leqslant D_{2,\textbf{y}}\\ \gcd (d',\ell _{1})=1 \end{array}} \frac{ \# V_{\ell _2\ell _3 d'e'}^{\times } }{d'^3} \textrm{d}\textbf{y}\end{aligned} \end{aligned}$$

and

6.14 $$\begin{aligned} D_{1,\textbf{y}}= \frac{|\textbf{y}|^2\max (|Q_1(\textbf{y})|,|Q_2(\textbf{y})|)}{\ell _3 B}, \quad D_{2,\textbf{y}}= \frac{\max (|Q_1(\textbf{y})|,|Q_2(\textbf{y})|)}{\ell _3 B^{\frac{1}{4}+\eta }}.\qquad \end{aligned}$$

Here, we have observed that the condition $D_1/\ell _1\ll d'\ll D_2/\ell _3$ is implied by the condition $D_{1,\textbf{y}}\leqslant d' \leqslant D_{2,\textbf{y}}$, since $Z({\mathbb {R}})= \emptyset$. We are therefore led to prove the following result.

#### Lemma 6.6

Let $Z_2>Z_1>0$ and let $c,\ell \in {\mathbb {N}}$ be square-free coprime integers. Then

$$\begin{aligned} \begin{aligned} \sum _{\begin{array}{c} Z_1\leqslant d\leqslant Z_2\\ (d,\ell )=1 \end{array}}\frac{\#V_{cd}^{\times }}{ d^{3}}&=C\#V_{c}^{\times }h_1(c) h_2(\ell )\left( \frac{1}{Z_1}-\frac{1}{Z_2}\right) +O_{\varepsilon }(c^{1+\varepsilon }Z_1^{-\frac{3}{2}+\varepsilon }), \end{aligned} \end{aligned}$$

for any $\varepsilon >0$, where

$$\begin{aligned} \begin{aligned} C&=\prod _{p}\left( 1-\frac{1}{p}\right) \left( 1+\sum _{a=1}^{\infty }\frac{\#V_{p^{a}}^{\times }}{p^{2a}}\right) \\ h_1(c)&=\prod _{p|c}\left( 1+\sum _{a=1}^{\infty }\frac{\#V_{p^{a}}^{\times }}{p^{2a}}\right) ^{-1} \left( 1+\frac{1}{\#V_{p}^{\times }}\sum _{a=1}^{\infty }\frac{\#V_{p^{a+1}}^{\times }}{p^{2a}}\right) \\ h_2(\ell )&=\prod _{p|\ell }\left( 1+\sum _{a=1}^{\infty }\frac{\#V_{p^{a}}^{\times }}{p^{2a}}\right) ^{-1}. \end{aligned} \end{aligned}$$

Moreover, $h_1(c)=O_\varepsilon (c^\varepsilon )$ and $h_2(\ell )=O_\varepsilon (\ell ^\varepsilon )$, for any $\varepsilon >0$.

#### Proof

If $0<Z_1<1$, the result is trivial, so we may assume $Z_1\geqslant 1$. We begin by defining the multiplicative function

$$\begin{aligned} f(p^{a})= {\left\{ \begin{array}{ll} p^{-a}\#V_{p^{a}}^{\times } &{}\text { if}\ p\not \mid c\ell ,\\ p^{-a}\#V_{p^{a+1}}^{\times }/\#V_{p}^{\times } &{}\text { if}\ p\mid c,\\ 0 &{}\text { if}\ p\mid \ell . \end{array}\right. } \end{aligned}$$

It follows form the Hasse bound that $\#V_{p}^{\times }=p-T_{p}+1,$ where $T_{p}=O(\sqrt{p})$. Moreover, the Chinese remainder theorem implies that

$$\begin{aligned} f(d)=\frac{ \#V_{cd}^{\times }}{d\#V_{c}^{\times }} \end{aligned}$$

for any $d\in {\mathbb {N}}$ such that $\gcd (d,\ell )=1$. Hence we can write

$$\begin{aligned} \sum _{\begin{array}{c} Z_1\leqslant d\leqslant Z_2\\ (d,\ell )=1 \end{array}}\frac{\#V_{cd}^{\times }}{ d^{3}}= \#V_{c}^{\times } \sum _{\begin{array}{c} Z_1\leqslant d\leqslant Z_2 \end{array}}\frac{f(d)}{ d^{2}}. \end{aligned}$$

We claim that

6.15 $$\begin{aligned} \sum _{d\leqslant x} f(d) =C h_1(c)h_2(\ell ) x +O\left( x^{\frac{1}{2}+\varepsilon }\right) , \end{aligned}$$

for any $\varepsilon >0$, where $C, h_1,h_2$ are defined in the statement of the lemma. Once achieved, on recalling the bound $\#V_c^\times =O_\varepsilon (c^{1+\varepsilon })$ from Lemma 3.5, an application of partial summation easily leads to the statement of the lemma. Finally, the bounds on $h_1$ and $h_2$ in the last part of the lemma follow easily from Lemma 3.5.

To prove (6.15), we write $$f=1*g$$ as a Dirichlet convolution, noting that $$ g(p^a)= f(p^a)-f(p^{a-1})$$ for any prime *p* and $$a\in {\mathbb {N}}$$. Suppose first that $$p\not \mid \Delta $$. Then it follows from Corollary 3.6 that

$$\begin{aligned} g(p^a) = {\left\{ \begin{array}{ll} O(p^{-\frac{1}{2}}) &{}\text { if}\ a=1\ \text {and}\ p\not \mid \ell ,\\ -1 &{}\text { if}\ a=1\ \text {and}\ p\mid \ell ,\\ 0 &{}\text { otherwise}. \end{array}\right. } \end{aligned}$$

On the other hand, if $p\mid \Delta$ then (3.13) and Lemma 3.4 together yield $g(p^a) =O(a).$ Given any $k\in {\mathbb {N}}$, it therefore follows that $g(k)\ll _\varepsilon k^\varepsilon /\sqrt{k_1},$ where $k_1$ is the part of *k* that is coprime to $\ell \Delta$. Given this, we easily conclude that

$$\begin{aligned} \sum _{d\leqslant x} f(d)&=\sum _{k\leqslant x} g(k) \left( \frac{x}{k} +O(1)\right) =\gamma x+O(x^{\frac{1}{2}+\varepsilon }), \end{aligned}$$

for any $\varepsilon >0$, where

$$\begin{aligned} \gamma =\sum _{k=1}^\infty \frac{g(k)}{k}= \prod _p \left( 1+\sum _{a=1}^\infty \frac{g(p^a)}{p^a}\right) = \prod _p \left( 1-\frac{1}{p}\right) \left( 1+\sum _{a=1}^\infty \frac{f(p^a)}{p^a}\right) . \end{aligned}$$

Inserting the definition of $f(p^a)$, it is straightforward to see that $\gamma =Ch_1(c)h_2(\ell )$, as required to complete the proof of the lemma. $\square$

We now seek to apply this in (6.13). Note from (6.14) that $D_{1,\textbf{y}}\gg Y^4/(\ell _3B)$, since we are assuming that $Z({\mathbb {R}})=\emptyset$. Hence we obtain

$$\begin{aligned} Q_{\ell _1,\ell _2,\ell _3,e'} =~&C\#V_{\ell _2\ell _3e'}^{\times }h_1(\ell _2\ell _3e') h_2(\ell _1) \nu (Y) +O_\varepsilon \left( \frac{(\ell _2e')^{1+\varepsilon }\ell _3^{\frac{5}{2}}B^{\frac{3}{2}+\varepsilon }}{Y^{2}}\right) , \end{aligned}$$

where

$$\begin{aligned} \nu (Y)=\int _{\begin{array}{c} \textbf{y}\in {\mathbb {R}}^4\\ |\textbf{y}|\sim Y \end{array}} \left( \frac{1}{D_{1,\textbf{y}}}-\frac{1}{D_{2,\textbf{y}}}\right) \textrm{d}\textbf{y}\end{aligned}$$

and $D_{1,\textbf{y}}, D_{2,\textbf{y}}$ are given by (6.14). Clearly $\nu (Y) =\ell _3 B \sigma _\infty (Y) +O(\ell _3 B^{\frac{1}{4}+\eta } Y^2),$ in the notation of (6.3). Thus $Q_{\ell _1,\ell _2,\ell _3,e'}$ is equal to

$$\begin{aligned} C\#V_{\ell _2\ell _3e'}^{\times }h_1(\ell _2\ell _3e') h_2(\ell _1) \ell _3 B\left( \sigma _\infty (Y)+O\left( \frac{Y^2}{B^{\frac{3}{4}-\eta }}\right) \right) +O_\varepsilon \left( \frac{(\ell _2e')^{1+\varepsilon }\ell _3^{\frac{5}{2}}B^{\frac{3}{2}+\varepsilon }}{Y^{2}}\right) . \end{aligned}$$

It is now time to insert this estimate into (6.12). First, the overall contribution from the error term is

$$\begin{aligned} \ll _\varepsilon \frac{B^{\frac{3}{2}+\varepsilon }}{Y^{2}} \sum _{\begin{array}{c} \ell _{1}\mid P(z)\\ \ell _{2}\mid P(z)\\ \ell _3\mid P(z) \end{array}}\frac{1}{\ell _1^4\ell _{2}^{3-\varepsilon }\ell _{3}^\frac{3}{2}} \sum _{\begin{array}{c} e'\mid \frac{P(z)}{\ell _{1}\ell _2} \end{array}} \frac{1}{e'^{2-\varepsilon }} \ll _\varepsilon \frac{B^{\frac{3}{2}+\varepsilon }}{Y^{2}}. \end{aligned}$$

Adjoining the contribution from the main term, we therefore obtain

6.16 $$\begin{aligned} T_0= C \cdot J(z) \cdot B\left( \sigma _\infty (Y)+O\left( \frac{Y^2}{B^{\frac{3}{4}-\eta }}\right) \right) + O_\varepsilon \left( \frac{B^{\frac{3}{2}+\varepsilon }}{Y^{2}} \right) , \end{aligned}$$

for any $\varepsilon >0$, where

$$\begin{aligned} J(z)&= \sum _{\begin{array}{c} \ell _{1}\mid P(z)\\ \ell _{2}\mid P(z)\\ \ell _3\mid P(z) \end{array}} \frac{\mu (\ell _{1}\ell _2\ell _{3})\mu (\ell _2) h_1(\ell _2\ell _3e') h_2(\ell _1) }{\ell _1^4\ell _{2}^4\ell _{3}^3} \hspace{-0.3cm} \sum _{\begin{array}{c} e'\mid \frac{P(z)}{\ell _{1}\ell _2}\\ \gcd (e',\ell _{3})=1 \end{array}} \hspace{-0.3cm} \frac{\mu (e')}{e'^3} \cdot \#V_{\ell _2\ell _3e'}^{\times }\\&= \sum _{\begin{array}{c} m\mid P(z) \end{array}} \sum _{\begin{array}{c} \ell _1,\dots ,\ell _4\in {\mathbb {N}}\\ \ell _1\cdots \ell _4 =m \end{array}} \hspace{-0.2cm} \frac{\mu (\ell _1)h_2(\ell _1)}{\ell _1^4} \cdot \frac{\mu (\ell _2)^2h_1(\ell _2)\#V_{\ell _2}^{\times }}{\ell _2^4} \frac{\mu (\ell _3)h_1(\ell _3) \#V_{\ell _3}^{\times }}{\ell _3^3}\\&\quad \cdot \frac{\mu (\ell _4)h_1(\ell _4) \#V_{\ell _4}^{\times }}{\ell _4^3}. \end{aligned}$$

In view of the second part of Lemma 3.5 and the bounds on $h_1$ and $h_2$ from Lemma 6.6, we can extend the sum over *m* to all square-free integers, finding that

$$\begin{aligned} J(z)=J+O(z^{-1}), \end{aligned}$$

where

$$\begin{aligned} J&= \prod _{p} \left( 1-\frac{h_2(p)}{p^{4}}+\frac{h_1(p)\#V_{p}^{\times }}{p^{4}}-\frac{2h_1(p)\#V_{p}^{\times }}{p^{3}}\right) \\&=\prod _{p} \left( 1+\sum _{a=1}^{\infty }\frac{\#V_{p^{a}}^{\times }}{p^{2a}}\right) ^{-1} \left( 1+\sum _{a=1}^{\infty }\frac{\#V_{p^{a}}^{\times }}{p^{2a}}-\frac{1}{p^{4}} +\left( \frac{1}{p^{2}}-\frac{2}{p}\right) \sum _{a=1}^{\infty }\frac{\#V_{p^{a}}^{\times }}{p^{2a}}\right) . \end{aligned}$$

Recalling the definition of *C* from the statement of Lemma  6.6, it follows that $CJ=\mathfrak {S}_1$, in the notation of (2.8). Returning to (6.16) and observing that $z\geqslant \sqrt{\log B}$, it therefore follows that

$$\begin{aligned} T_0= \mathfrak {S}_1 B \left( \sigma _\infty (Y)+O\left( \frac{Y^2}{B^{\frac{3}{4}-\eta }}\right) \right) +O\left( \frac{B}{\sqrt{\log B}} \right) + O_\varepsilon \left( \frac{B^{\frac{3}{2}+\varepsilon }}{Y^{2}} \right) , \end{aligned}$$

where $\sigma _\infty (Y)$ is given by (6.3). Substituting this into Lemma 6.5, and using the lower bound $Y\gg B^{\frac{1}{4}+\frac{\eta }{2}}$, we are finally led to the statement of Proposition 6.1.

## Asymptotics via the circle method

The goal of this section is to prove Proposition 2.3. Recall the notation $\varDelta (\textbf{x})=\prod _{i=1}^4L_i(\textbf{x})$ from (5.16). For any $\textbf{x}$ and any compactly supported weight function $w:{\mathbb {R}}^4\rightarrow {\mathbb {R}}_{\geqslant 0}$, the singular integral is defined to be

$$\begin{aligned} \sigma _{\infty ,w}(\textbf{x})=\int _{-\infty }^{+\infty }\int _{{{\mathbb {R}}}^4}w(\textbf{y}){\text {e}}\left( -\theta (L_1(\textbf{x})y_1^2+\cdots + L_4(\textbf{x})y_4^2)\right) {\text {d}}\textbf{y}{\text {d}}\theta . \end{aligned}$$

In the special case that $w_0$ is the characteristic function of $[-1,1]^4$, we set $\sigma _{\infty }(\textbf{x})=\sigma _{\infty ,w_0}(\textbf{x}).$ We have $\sigma _\infty (\lambda \textbf{x})=\lambda ^{-1}\sigma _\infty (\textbf{x})$, for any $\lambda >0$. Moreover, it follows from [5, Lemma 4.12] that

7.1 $$\begin{aligned} \sigma _{\infty }(\textbf{x}) \ll \frac{1}{|\varDelta (\textbf{x})|^{1/4}} \quad \text { and }\quad \sigma _{\infty ,w}(\textbf{x}) \ll \frac{1}{|\varDelta (\textbf{x})|^{1/4}}, \end{aligned}$$

for any compactly supported smooth weight function $w:{\mathbb {R}}^4\rightarrow {\mathbb {R}}_{\geqslant 0}$.

Finally, we put

$$\begin{aligned} \mathfrak {S}(\textbf{x})=\mathfrak {S}(Q_{\textbf{x}})=\prod _p \sigma _p(\textbf{x}), \end{aligned}$$

where

$$\begin{aligned} \sigma _p(\textbf{x})=\lim _{k\rightarrow \infty }\frac{\#\left\{ \textbf{y}\in ({\mathbb {Z}}/p^k{\mathbb {Z}})^4: L_1(\textbf{x})y_1^2+\cdots + L_4(\textbf{x})y_4^2\equiv 0 \bmod {p^k} \right\} }{p^{3k}}. \end{aligned}$$

With this notation to hand we may now record the first main result of this section, which closely follows the strategy in [5, § 5].

### Proposition 7.1

Let $$\eta >0$$. Then

$$\begin{aligned} \#{{\mathscr {L}}}_2(B) =~&\frac{B}{\zeta (2)}\sum _{\begin{array}{c} \textbf{x}\in {{\mathbb {Z}}}_{{\text {prim}}}^2\\ B^{2\eta }\leqslant |\textbf{x}|\leqslant B^\frac{1}{4}\\ \prod _{i=1}^{4}L_i(\textbf{x})\ne \square \end{array}} \frac{\sigma _{\infty }(\textbf{x})\mathfrak {S}(\textbf{x})}{|\textbf{x}|}+O(\eta ^\frac{1}{2} B\log B)+O_\eta (B^{1-\frac{\eta ^2}{32}}). \end{aligned}$$

The asymptotic behaviour of the leading term in Proposition  7.1 is our next milestone and is summarised in the following result.

### Proposition 7.2

Let $$\eta >0$$. Then

$$\begin{aligned} \sum _{\begin{array}{c} \textbf{x}\in {{\mathbb {Z}}}_{{\text {prim}}}^2\\ B^{2\eta }\leqslant |\textbf{x}|\leqslant B^\frac{1}{4}\\ \prod _{i=1}^{4}L_i(\textbf{x})\ne \square \end{array}}\frac{\sigma _{\infty }(\textbf{x})\mathfrak {S}(\textbf{x})}{|\textbf{x}|} = \frac{\tau _\infty \mathfrak {S}_2}{4\zeta (2)}\log B+O(\eta \log B)+O_\eta (1), \end{aligned}$$

where $\tau _\infty$ is given by (2.9) and $\mathfrak {S}_2$ is given by (2.11).

Combining Propositions 7.1 and 7.2 concludes the proof of Proposition 2.3.

### Proof of Proposition 7.1

Let $w:{\mathbb {R}}^4\rightarrow {\mathbb {R}}_{\geqslant 0}$ be a compactly supported weight function. Then for any $X\geqslant 1$, we define the weighted counting function

7.2 $$\begin{aligned} L_w(B,X)=\sum _{\begin{array}{c} \textbf{x}\in {{\mathbb {Z}}}_{{\text {prim}}}^2\\ |\textbf{x}|\sim X\\ \varDelta (\textbf{x})\ne \square \end{array}}\sum _{\begin{array}{c} \textbf{y}\in {{\mathbb {Z}}}_{{\text {prim}}}^4\\ (1.1) \text { holds} \end{array}} w\left( \frac{|\textbf{x}|^\frac{1}{2}}{B^\frac{1}{2}}\textbf{y}\right) . \end{aligned}$$

When $w=w_0$, as above, then

$$\begin{aligned} \#{{\mathscr {L}}}_2(B)=\sum _{X\nearrow B^{\frac{1}{4}}}L_{w_0}(B,X). \end{aligned}$$

Our strategy for proving Proposition 7.1 is to first produce an asymptotic formula for $L_w(B,X)$ when *w* is a suitable smooth weight, before finally showing how to approximate the counting function $L_{w_0}(B,X)$ by smoothly weighted ones. It turns out that the circle method tools required to produce an asymptotic formula for $L_w(B,X)$ are already in exactly the right form in [5]. This allows us to prove the following analogue of [5, Lemma 5.3].

#### Lemma 7.3

Let $w:{\mathbb {R}}^4\rightarrow {\mathbb {R}}_{\geqslant 0}$ be a compactly supported weight function which vanishes on $[-\eta ,\eta ]^4$. Suppose that $X\geqslant 1$ satisfies

7.3 $$\begin{aligned} B^{2\eta }\ll X\ll B^{\frac{1}{4}-4\eta }. \end{aligned}$$

Then, for $\eta >0$ sufficiently small, we have

$$\begin{aligned} L_w(B,X)=\frac{B}{\zeta (2)}\sum _{\begin{array}{c} \textbf{x}\in {{\mathbb {Z}}}_{{\text {prim}}}^2\\ |\textbf{x}|\sim X\\ \varDelta (\textbf{x})\ne \square \end{array}}\frac{\sigma _{\infty ,w}(\textbf{x})\mathfrak {S}(\textbf{x})}{|\textbf{x}|}+O_{\eta ,w}(B^{1-\frac{1}{16}\eta ^2}). \end{aligned}$$

#### Proof

For $\textbf{x}\in {{\mathbb {Z}}}_{{\text {prim}}}^2$ and $Y\gg 1$, we define

$$\begin{aligned} N_w(Q_\textbf{x};Y)=\sum _{\begin{array}{c} \textbf{y}\in {{\mathbb {Z}}}_{{\text {prim}}}^4\\ Q_\textbf{x}(\textbf{y})=0 \end{array}} w\left( \frac{\textbf{y}}{Y}\right) , \end{aligned}$$

where $Q_\textbf{x}$ is given by (5.12). Then

$$\begin{aligned} L_w(B,X)=\sum _{\begin{array}{c} \textbf{x}\in {{\mathbb {Z}}}_{{\text {prim}}}^2\\ |\textbf{x}|\sim X\\ \varDelta (\textbf{x})\ne \square \end{array}}N_w\left( Q_\textbf{x};\sqrt{\frac{B}{|\textbf{x}|}}\right) . \end{aligned}$$

As in (5.16), we write $\Vert Q_\textbf{x}\Vert =\max _{1\leqslant i\leqslant 4}|L_i(\textbf{x})|$ and we recall the definition (5.1) of $\varDelta _{{\text {bad}}}(\textbf{x})$.

It now follows from [5, Lemma 5.2]

$$\begin{aligned} N_w(Q_\textbf{x};Y)= \frac{\sigma _{\infty ,w}(\textbf{x})\mathfrak {S}(\textbf{x})}{\zeta (2)}Y^2+O_{\eta ,w} \left( \frac{Y^{\frac{5}{3}+5\eta }}{\Vert Q_\textbf{x}\Vert ^\frac{1}{2}}\right) , \end{aligned}$$

for any $Y\geqslant 1$, provided that $Y^\eta \leqslant \Vert Q_\textbf{x}\Vert \leqslant Y^\frac{2}{3}$, $|L_i(\textbf{x})|\geqslant \Vert Q_\textbf{x}\Vert ^{1-\eta }$ for every $1\leqslant i\leqslant 4$, and $\varDelta _{{\text {bad}}}(\textbf{x})\leqslant \Vert Q_\textbf{x}\Vert ^\eta$. Observing that $|\textbf{x}|\ll \Vert Q_\textbf{x}\Vert \ll |\textbf{x}|$, we deduce that

$$\begin{aligned} N_w(Q_\textbf{x};Y)=\frac{\sigma _{\infty ,w}(\textbf{x})\mathfrak {S}(\textbf{x})}{\zeta (2)}Y^2+O_{\eta ,w} \left( \frac{Y^{\frac{5}{3}+5\eta }}{|\textbf{x}|^\frac{1}{2}}\right) , \end{aligned}$$

provided that

7.4 $$\begin{aligned}{} & {} Y^\eta \ll |\textbf{x}|\ll Y^\frac{2}{3}, \end{aligned}$$

7.5 $$\begin{aligned}{} & {} |L_i(\textbf{x})|\gg |\textbf{x}|^{1-\eta }, \text { for every } 1\leqslant i\leqslant 4, \end{aligned}$$

and

7.6 $$\begin{aligned} \varDelta _{{\text {bad}}}(\textbf{x})\ll |\textbf{x}|^\eta . \end{aligned}$$

Under the assumption that *X* satisfies (7.3), the condition (7.4) is always satisfied with $Y=\sqrt{B/|\textbf{x}|}$. Hence it follows that

7.7 $$\begin{aligned} \begin{aligned} \sum _{\begin{array}{c} \textbf{x}\in {{\mathbb {Z}}}_{{\text {prim}}}^2,~|\textbf{x}|\sim X\\ \varDelta (\textbf{x})\ne \square \\ (7.5) (7.6)\text {hold} \end{array}} \sum _{\begin{array}{c} \textbf{y}\in {{\mathbb {Z}}}_{{\text {prim}}}^4\\ (1.1) \text {holds} \end{array}} w\left( \frac{|\textbf{x}|^\frac{1}{2}}{B^\frac{1}{2}}\textbf{y}\right) =~&\frac{B}{\zeta (2)}\sum _{\begin{array}{c} \textbf{x}\in {{\mathbb {Z}}}_{{\text {prim}}}^2,|\textbf{x}|\sim X\\ \varDelta (\textbf{x})\ne \square \\ (7.5) (7.6)\text {hold} \end{array}} \frac{\sigma _{\infty ,w}(\textbf{x})\mathfrak {S}(\textbf{x})}{|\textbf{x}|}\\&\quad +O_{\eta ,w}(B^{\frac{5}{6}+\frac{5}{2}\eta }X^\frac{2}{3}). \end{aligned} \end{aligned}$$

Note that the error term is $O( B^{1-\frac{1}{6}\eta })$, since $X\ll B^{\frac{1}{4}-4\eta }$ in (7.3).

It remains to treat the cases where either (7.5) or (7.6) fails. We start with (7.5) and assume without loss of generality that $|L_{1}(\textbf{x})|\ll |\textbf{x}|^{1-\eta }$. Then it follows from Lemma 5.8, with $\delta =1-\eta$ and $Y=\sqrt{B/X}$, that the overall contribution is

$$\begin{aligned}&\ll _{\eta ,\varepsilon } B^\varepsilon X^{-\frac{1}{2}\eta }\left( B+B^\frac{1}{2}X^2\right) \ll X^{-\frac{1}{2}\eta } B^{1+\varepsilon } \ll B^{1-\eta ^2+\varepsilon }, \end{aligned}$$

under the assumption (7.3). On taking $\varepsilon =\eta ^2/2$, the contribution from this case is therefore $O_{\eta }(B^{1-\eta ^2/2})$. To handle the situation when (7.6) fails, we use Proposition 5.3. Taking $D\gg X^\eta$, we therefore obtain the contribution

$$\begin{aligned}&\ll _{\varepsilon } B^\varepsilon \left( \frac{B+B^\frac{1}{2}X^2}{X^{\frac{\eta }{16}}}+XB^\frac{1}{2}+ B^\frac{2}{3}\right) \ll X^{-\frac{\eta }{16}}B^{1+\varepsilon }+B^{\frac{3}{4}+\varepsilon } \ll B^{1-\frac{\eta ^2}{8}+\varepsilon }. \end{aligned}$$

Taking $\varepsilon =\eta ^2/{16}$, we obtain the overall contribution $O_\eta (B^{1-\eta ^2/16})$.

It remains to extend the sum of $\textbf{x}\in {{\mathbb {Z}}}_{{\text {prim}}}^2$ in (7.7) to the whole range. For this purpose, we consider the sums

7.8 $$\begin{aligned} S_1(X)=\sum _{\begin{array}{c} \textbf{x}\in {{\mathbb {Z}}}_{{\text {prim}}}^2, ~\varDelta (\textbf{x})\ne \square \\ |\textbf{x}|\sim X,~|L_1(\textbf{x})|\ll X^{1-\eta } \end{array}}\frac{\mathfrak {S}(\textbf{x})}{|\textbf{x}||\prod _{i=1}^4L_i(\textbf{x})|^\frac{1}{4}} \end{aligned}$$

and

7.9 $$\begin{aligned} S_2(X)=\sum _{\begin{array}{c} \textbf{x}\in {{\mathbb {Z}}}_{{\text {prim}}}^2,~\varDelta (\textbf{x})\ne \square \\ |\textbf{x}|\sim X,~\varDelta _{{\text {bad}}}(\textbf{x})\gg X^{\eta } \end{array}}\frac{\mathfrak {S}(\textbf{x})}{|\textbf{x}||\prod _{i=1}^4L_i(\textbf{x})|^\frac{1}{4}}. \end{aligned}$$

In both of these sums we can apply Lemma 4.3 to estimate $\mathfrak {S}(\textbf{x})$.

We start by estimating (7.8). Combining Lemmas  4.3 and 5.7 with (5.27), we obtain

$$\begin{aligned} S_1(X)&\ll _{\varepsilon ,\eta } \frac{1}{X^\frac{7}{4}}\sum _{\begin{array}{c} \textbf{x}\in {{\mathbb {Z}}}_{{\text {prim}}}^2,~ \varDelta (\textbf{x})\ne \square \\ |\textbf{x}|\sim X,~|L_1(\textbf{x})|\ll X^{1-\eta } \end{array}} \frac{\varDelta _{{\text {bad}}}(\textbf{x})^\varepsilon L(1,\chi _{Q_\textbf{x}})}{|L_1(\textbf{x})|^\frac{1}{4}}\\&\ll _{\varepsilon ,\eta } \frac{X^{5\varepsilon }}{X^\frac{7}{4}} \sum _{R\nearrow X^{1-\eta }} \frac{1}{R^\frac{1}{4}}\sum _{\begin{array}{c} \textbf{x}\in {{\mathbb {Z}}}_{{\text {prim}}}^2,~\varDelta (\textbf{x})\ne \square \\ |\textbf{x}|\sim X,~|L_1(\textbf{x})|\sim R \end{array}} 1, \end{aligned}$$

on introducing a dyadic parameter for the range of $|L_1(\textbf{x})|$. It follows easily that

$$\begin{aligned} S_1(X) \ll _{\varepsilon ,\eta } \frac{X^{5\varepsilon }}{X^\frac{7}{4}} \sum _{R\nearrow X^{1-\eta }} XR^{\frac{3}{4}} \ll X^{-\frac{3}{4}\eta +5\varepsilon } \ll X^{-\frac{\eta }{2}}, \end{aligned}$$

by fixing $\varepsilon$ to be sufficiently small.

Now we handle $S_2(X)$ similarly. It follows from Lemma  4.3 and (5.27) that

$$\begin{aligned} S_2(X)&\ll _\varepsilon \frac{X^{5\varepsilon }}{X^\frac{7}{4}} \sum _{i_1=1}^4 \sum _{R\nearrow X} \frac{1}{R^\frac{1}{4}}\sum _{\begin{array}{c} \textbf{x}\in {{\mathbb {Z}}}_{{\text {prim}}}^2,~\varDelta (\textbf{x})\ne \square \\ |\textbf{x}|\sim X, ~|L_{i_1}(\textbf{x})|\sim R\\ \varDelta _{{\text {bad}}}(\textbf{x})\gg X^\eta \end{array}}1. \end{aligned}$$

The condition $\varDelta _{{\text {bad}}}(\textbf{x})\gg X^\eta$ implies in particular $\varDelta _{{\text {bad}}}(\textbf{x})\gg (XR)^{\frac{\eta }{2}}$. Applying Lemma  5.9 with $\delta =\frac{\eta }{2}$ and $S=X$, we obtain

$$\begin{aligned} S_2(X)\ll _\varepsilon \frac{X^{5\varepsilon }}{X^\frac{7}{4}}\sum _{R\nearrow X} \frac{X R^{1-\frac{\eta }{16}}}{R^\frac{1}{4}}\ll X^{-\frac{\eta }{16}+5\varepsilon }. \end{aligned}$$

Thus $S_2(X)\ll X^{-\frac{\eta }{32}},$ on taking $\varepsilon$ sufficiently small.

Invoking the bound (7.1) for $\sigma _{\infty ,w}(\textbf{x})$, we may now apply our bounds for $S_1(X)$ and $S_2(X)$ to deduce that there is a satisfactory overall contribution to the main term in (7.7), corresponding to the failure of (7.5) or (7.6). The proof of the lemma is now completed. $\square$

It remains to remove the smooth weights, using the previous result to deduce a similar asymptotic formula for the counting function $L_{w_0}(B,X)$, where $w_0$ is the characteristic function of $[-1,1]^4$.

#### Lemma 7.4

Assume that *X* lies in the range (7.3). Then, for $\eta >0$ sufficiently small, we have

$$\begin{aligned} L_{w_0}(B,X)=\frac{B}{\zeta (2)}\sum _{\begin{array}{c} \textbf{x}\in {{\mathbb {Z}}}_{{\text {prim}}}^2\\ |\textbf{x}|\sim X\\ \varDelta (\textbf{x})\ne \square \end{array}}\frac{\sigma _{\infty }(\textbf{x})\mathfrak {S}(\textbf{x})}{|\textbf{x}|}+O(\eta ^\frac{1}{2}B)+O_{\eta }(B^{1-\frac{1}{16}\eta ^2}). \end{aligned}$$

#### Proof

We mimic the procedure of [5, §5.3], which relates the counting function $L_{w_0}(B,X)$ to one in which smooth weights appear. For each $\eta >0$ sufficiently small, we fix two smooth weight functions $w_1,w_2$ satisfying the requirements of [5, Lemma 4.13]. Thus

7.10 $$\begin{aligned} \sigma _{\infty ,w_i}(\textbf{x})-\sigma _{\infty }(\textbf{x})\ll \frac{\eta ^\frac{1}{2}}{\prod _{i=1}^{4}|L_i(\textbf{x})|^\frac{1}{4}}. \end{aligned}$$

Moreover,

$$\begin{aligned} L_{w_1}(B,X)\leqslant L_{w_0}(B,X)\leqslant L_{w_0}(4\eta ^2B,X)+L_{w_2}(B,X). \end{aligned}$$

We can apply Lemma 7.3 to estimate $L_{w_1}(B,X)$ and $L_{w_2}(B,X)$. Moreover, on recalling (7.2), we deduce from Theorem 2.1 that

$$\begin{aligned} L_{w_0}(4\eta ^2B,X)&\ll \eta ^2 B +\eta ^{4/3}X^{4/3}B^{2/3}\ll \eta B, \end{aligned}$$

since $$X\ll B^\frac{1}{4}$$ in (7.3).

In view of (7.10), for $i=1,2$, it remains to show that

7.11 $$\begin{aligned} S_0(X)=\sum _{\begin{array}{c} \textbf{x}\in {{\mathbb {Z}}}_{{\text {prim}}}^2\\ |\textbf{x}|\sim X\\ \varDelta (\textbf{x})\ne \square \end{array}}\frac{\mathfrak {S}(\textbf{x})}{|\textbf{x}||\prod _{i=1}^4L_i(\textbf{x})|^\frac{1}{4}}=O(1), \end{aligned}$$

in order to complete the proof of the lemma. We can adopt a similar argument to the treatment of $S_1(X)$ and $S_2(X)$ in (7.8) and (7.9), respectively. Appealing to Lemma 4.3 and breaking into dyadic intervals, we obtain

$$\begin{aligned} S_0(X)&\ll \sum _{i_1=1}^{4}\frac{1}{X^{\frac{7}{4}}}\sum _{R \nearrow X}\frac{1}{R^\frac{1}{4}}\sum _{\begin{array}{c} \textbf{x}\in {{\mathbb {Z}}}_{{\text {prim}}}^2\\ |\textbf{x}|\sim X,~|L_{i_1}(\textbf{x})|\sim R\\ \varDelta (\textbf{x})\ne \square \end{array}}\varDelta _{{\text {bad}}}(\textbf{x})^\varepsilon L(1,\chi _{Q_\textbf{x}}). \end{aligned}$$

Recalling (5.26), an application of Lemma 5.10 with $\delta =\frac{1}{16}$ and $S=X$, therefore yields

$$\begin{aligned} S_0(X)&\ll _{\varepsilon } \frac{1}{X^{\frac{7}{4}}}\sum _{R \nearrow X}\frac{1}{R^\frac{1}{4}} \left( XR +X^{\frac{33}{32}+\varepsilon }R^{\frac{1}{32}}+X^{\frac{11}{16}+\varepsilon }R^{\frac{5}{4}}\right) \\&\ll \frac{1}{X^{\frac{7}{4}}}\left( X^\frac{7}{4}+X^\varepsilon (X^\frac{27}{16}+X^{\frac{33}{32}})\right) . \end{aligned}$$

Thus $S_0(X)=O(1)$ on fixing $\varepsilon$ to be small enough. This establishes (7.11), thereby completing the proof of the lemma. $\square$

We are now ready to deduce Proposition 7.1. Lemma 7.4 implies that

$$\begin{aligned} \sum _{B^{2\eta }\ll X\ll B^{\frac{1}{4}-4\eta }}L_{w_0}(B,X)&=\frac{B}{\zeta (2)}\sum _{\begin{array}{c} \textbf{x}\in {{\mathbb {Z}}}_{{\text {prim}}}^2\\ \varDelta (\textbf{x})\ne \square \\ B^{2\eta }\ll |\textbf{x}|\ll B^{\frac{1}{4}-4\eta } \end{array}}\frac{\sigma _{\infty }(\textbf{x})\mathfrak {S}(\textbf{x})}{|\textbf{x}|}\\&\quad +O(\eta ^\frac{1}{2}B\log B)+O_{\eta }(B^{1-\frac{1}{16}\eta ^2}\log B). \end{aligned}$$

We may clearly take $\log B=O(B^{\frac{1}{32}\eta ^2})$ in the second error term. Moreover, on recalling (7.11), we obtain

$$\begin{aligned} \frac{B}{\zeta (2)} \sum _{\begin{array}{c} \textbf{x}\in {{\mathbb {Z}}}_{{\text {prim}}}^2\\ \varDelta (\textbf{x})\ne \square \\ B^{\frac{1}{4}-4\eta } \ll |\textbf{x}|\ll B^\frac{1}{4} \end{array}}\frac{\sigma _{\infty }(\textbf{x})\mathfrak {S}(\textbf{x})}{|\textbf{x}|}&\ll B\sum _{B^{\frac{1}{4}-4\eta } \ll X\ll B^\frac{1}{4}} S_0(X)\ll \eta B\log B. \end{aligned}$$

Moreover, with further recourse to (7.11), it also follows that

$$\begin{aligned} \sum _{B^{2\eta }\ll X\ll B^{\frac{1}{4}-4\eta }}L_{w_0}(B,X)&=\frac{B}{\zeta (2)}\sum _{\begin{array}{c} \textbf{x}\in {{\mathbb {Z}}}_{{\text {prim}}}^2\\ \varDelta (\textbf{x})\ne \square \\ B^{2\eta }\leqslant |\textbf{x}|\leqslant B^{\frac{1}{4}} \end{array}}\frac{\sigma _{\infty }(\textbf{x})\mathfrak {S}(\textbf{x})}{|\textbf{x}|} +O(\eta ^\frac{1}{2}B\log B)\\&\quad +O_{\eta }(B^{1-\frac{1}{32}\eta ^2}). \end{aligned}$$

Finally, it follows from Theorem 2.1 that

$$\begin{aligned} \sum _{\begin{array}{c} X\ll B^{2\eta }\text { or}\\ B^{\frac{1}{4}-4\eta }\ll X\ll B^\frac{1}{4} \end{array}}L_{w_0}(B,X)\ll \eta B\log B. \end{aligned}$$

This therefore finishes the proof of Proposition 7.1.

### Proof of Proposition 7.2: preliminaries

In this section we are concerned with the asymptotic behaviour of the term

7.12 $$\begin{aligned} M(B)= \sum _{\begin{array}{c} \textbf{x}\in {{\mathbb {Z}}}_{{\text {prim}}}^2\\ B^{2\eta }\leqslant |\textbf{x}|\leqslant B^\frac{1}{4}\\ \prod _{i=1}^{4}L_i(\textbf{x})\ne \square \end{array}}\frac{\sigma _{\infty }(\textbf{x})\mathfrak {S}(\textbf{x})}{|\textbf{x}|}. \end{aligned}$$

The line of attack follows [5, §6.2], but we face extra difficulties that are similar to the ones we encountered in §5.1. The basic idea is to restrict each series $\mathfrak {S}(\textbf{x})$ to a sum over small moduli, before interchanging the order of summation. To achieve this, it will be crucial to achieve sufficient cancellation when averaging over the $\textbf{x}$-sum, which is harder in this setting, since we have half the number of $\textbf{x}$-variables compared to the variety (1.6) considered in [5].

For $\textbf{x}\in {{\mathbb {Z}}}_{{\text {prim}}}^2$ and $q\in {{\mathbb {N}}}$, we let

$$\begin{aligned} S_q(\textbf{x})=\sum _{\begin{array}{c} a \bmod {q}\\ \gcd (a,q)=1 \end{array} }\sum _{{\textbf{b}}\in ({{\mathbb {Z}}}/q{{\mathbb {Z}}})^4}{\text {e}}_q\left( a\sum _{i=1}^{4}L_i(\textbf{x})b_i^2\right) . \end{aligned}$$

This is multiplicative in *q* and we have $\mathfrak {S}(\textbf{x})=\sum _{q=1}^\infty q^{-4} S_q(\textbf{x}).$ It will be useful to collect together some estimates for $S_q(\textbf{x})$ that can be extracted from [5, §4.2].

#### Lemma 7.5

Let $\varepsilon >0$, let $\textbf{x}\in {{\mathbb {Z}}}_{{\text {prim}}}^2$ and let $q\in {\mathbb {N}}$. Then the following statements are true.

(i) We have $$S_q(\textbf{x})\ll q^3\gcd (q,\varDelta (\textbf{x}))^\frac{1}{2}.$$
(ii) If $p\not \mid 2\varDelta (\textbf{x})$ and $r\in {{\mathbb {N}}}$, then
  $$\begin{aligned} S_{p^r}(\textbf{x})=\left( \frac{\varDelta (\textbf{x})}{p^r}\right) p^{3r}\varphi ^*(p^r), \end{aligned}$$
  where $\varphi ^*(n)=\varphi (n)/n$. Moreover, $S_{p^r}(\textbf{x})=0$ if $p\mid \varDelta (\textbf{x})$ but $p\not \mid 2\varDelta _{{\text {bad}}}(\textbf{x})$.
(iii) We have
  $$\begin{aligned} \sum _{q\leqslant X}\frac{S_q(\textbf{x})}{q^3} \ll _{\varepsilon } |\varDelta (\textbf{x})|^{\frac{3}{16}+\varepsilon }\varDelta _{{\text {bad}}}(\textbf{x})^\frac{3}{8}X^{\frac{1}{2}+\varepsilon }, \end{aligned}$$
  if $$\varDelta (\textbf{x})\ne \square $$.
(iv) We have
  $$\begin{aligned} \sum _{q\leqslant X}\frac{|S_q(\textbf{x})|}{q^4} \ll _{\varepsilon } X^\varepsilon . \end{aligned}$$

#### Proof

It follows from [5, Lemma 4.5] that $S_q(\textbf{x})\ll q^3\prod _{i=1}^{4}\gcd (q,L_i(\textbf{x}))^\frac{1}{2}.$ But then part (i) is a consequence of the observation (5.9) and the fact that $\textbf{x}$ is primitive. The formulae in (ii) follow from [5, Lemmas 4.6 and 4.8]. Part (iii) is the same as [5, Lemma 4.9]. It remains to prove part (iv). Appealing to part (ii), we obtain

$$\begin{aligned} \sum _{q\leqslant X}\frac{|S_q(x)|}{q^4}&\ll \left| \sum _{\begin{array}{c} q_2\leqslant X\\ q_2\mid (2\varDelta _{{\text {bad}}}(\textbf{x}))^\infty \end{array}}\frac{|S_{q_2}(x)|}{q_2^4}\right| \left| \sum _{q_1\leqslant X/q_2}\frac{\varphi ^*(q_1)}{q_1}\right| \\&\ll \#\left\{ q_2\in {\mathbb {N}}: q_2\leqslant X, ~ q_2\mid (2\varDelta _{{\text {bad}}}(\textbf{x}))^\infty \right\} \log X, \end{aligned}$$

since part (i) implies that $S_{q_2}(\textbf{x})\ll q_2^{\frac{7}{2}}$. The remaining cardinality is easily seen to be $O_\varepsilon (X^\varepsilon \varDelta _{{\text {bad}}}(\textbf{x})^\varepsilon )$, which thereby completes the proof. $\square$

We now carry out the proof of Proposition 7.2 in a series of steps

#### Lemma 7.6

(Reduction to small $\varDelta _{{\text {bad}}}(\textbf{x})$) We have

$$\begin{aligned} \sum _{\begin{array}{c} \textbf{x}\in {{\mathbb {Z}}}_{{\text {prim}}}^2\\ B^{2\eta }\leqslant |\textbf{x}|\leqslant B^\frac{1}{4}\\ \varDelta (\textbf{x})\ne \square \end{array}}\frac{\sigma _{\infty }(\textbf{x})\mathfrak {S}(\textbf{x})}{|\textbf{x}|}=\sum _{\begin{array}{c} \textbf{x}\in {{\mathbb {Z}}}_{{\text {prim}}}^2\\ B^{2\eta }\leqslant |\textbf{x}|\leqslant B^\frac{1}{4}\\ \varDelta (\textbf{x})\ne \square \\ \varDelta _{{\text {bad}}}(\textbf{x})\leqslant B^{\eta /1000} \end{array}}\frac{\sigma _{\infty }(\textbf{x})\mathfrak {S}(\textbf{x})}{|\textbf{x}|}+O_\eta (1). \end{aligned}$$

#### Proof

We apply the bound (7.1) for $\sigma _\infty (\textbf{x})$, together with the bound $\mathfrak {S}(\textbf{x})\ll _{\varepsilon }|\textbf{x}|^{5\varepsilon }$, which follows from Lemma 4.3 and (5.27). On executing the dyadic decomposition in the same way as in the proof of Proposition 5.2, it follows from Lemma 5.9 that

$$\begin{aligned} \sum _{\begin{array}{c} \textbf{x}\in {{\mathbb {Z}}}_{{\text {prim}}}^2\\ B^{2\eta }\leqslant |\textbf{x}|\leqslant B^\frac{1}{4}\\ \varDelta (\textbf{x})\ne \square \\ \varDelta _{{\text {bad}}}(\textbf{x})> B^{\eta /1000} \end{array}}\frac{\sigma _{\infty }(\textbf{x})\mathfrak {S}(\textbf{x})}{|\textbf{x}|}&\ll _{\varepsilon } \sum _{B^{2\eta }\swarrow S\nearrow B^\frac{1}{4}}\sum _{R \nearrow S} \frac{S^{5\varepsilon }(SR)^{1-\eta /400}}{S^\frac{7}{4}R^\frac{1}{4}} \ll _{\varepsilon }B^{-\eta ^2/100+5\varepsilon }, \end{aligned}$$

since $\varDelta _{{\text {bad}}}(\textbf{x})>B^{\eta /1000}\geqslant (SR)^{\eta /500}$. The result follows on choosing $\varepsilon$ small enough. $\square$

#### Lemma 7.7

(First truncation of $\mathfrak {S}(\textbf{x})$) We have

$$\begin{aligned} \sum _{\begin{array}{c} \textbf{x}\in {{\mathbb {Z}}}_{{\text {prim}}}^2\\ B^{2\eta }\leqslant |\textbf{x}|\leqslant B^\frac{1}{4}\\ \varDelta (\textbf{x})\ne \square \end{array}}\frac{\sigma _{\infty }(\textbf{x})\mathfrak {S}(\textbf{x})}{|\textbf{x}|}=\sum _{\begin{array}{c} \textbf{x}\in {{\mathbb {Z}}}_{{\text {prim}}}^2\\ B^{2\eta }\leqslant |\textbf{x}|\leqslant B^\frac{1}{4}\\ \varDelta (\textbf{x})\ne \square \end{array}}\frac{\sigma _{\infty }(\textbf{x})\mathfrak {S}(\textbf{x};B^{100})}{|\textbf{x}|}+O(B^{-1}), \end{aligned}$$

where $$ \mathfrak {S}(\textbf{x};N) =\sum _{q\leqslant N}q^{-4}S_q(\textbf{x}).$$

#### Proof

Applying partial summation, it follows from part (iii) of Lemma 7.5 that

$$\begin{aligned} \sum _{q>B^{100}}\frac{S_q(\textbf{x})}{q^4}\ll |\varDelta (\textbf{x})|^\frac{3}{16}\varDelta _{{\text {bad}}}(\textbf{x})^\frac{3}{8}B^{-20}, \end{aligned}$$

uniformly for any $\textbf{x}\in {{\mathbb {Z}}}_{{\text {prim}}}^2$ such that $\Delta (\textbf{x})\ne \square$ and $|\textbf{x}|\leqslant B^{\frac{1}{4}}$. The bound (7.1) now yields

$$\begin{aligned} \sum _{\begin{array}{c} \textbf{x}\in {{\mathbb {Z}}}_{{\text {prim}}}^2\\ B^{2\eta }\leqslant |\textbf{x}|\leqslant B^\frac{1}{4}\\ \varDelta (\textbf{x})\ne \square \end{array}}\frac{\sigma _{\infty }(\textbf{x})}{|\textbf{x}|}\sum _{q>B^{100}}\frac{S_q(\textbf{x})}{q^4} \ll B^{-20}\sum _{\begin{array}{c} \textbf{x}\in {{\mathbb {Z}}}^2\\ |\textbf{x}|\leqslant B^\frac{1}{4}\\ \varDelta (\textbf{x})\ne 0 \end{array}}|\varDelta (\textbf{x})|^{\frac{5}{16}}\ll B^{-1}, \end{aligned}$$

since $$\varDelta _{{\text {bad}}}(\textbf{x})\leqslant |\varDelta (\textbf{x})|\ll |\textbf{x}|^4.$$ $$\square $$

Having truncated the *q*-sum, we proceed to show that there is a negligible contribution from $\textbf{x}$ such that $\varDelta (\textbf{x})=\square$.

#### Lemma 7.8

We have

$$\begin{aligned} \sum _{\begin{array}{c} \textbf{x}\in {{\mathbb {Z}}}_{{\text {prim}}}^2\\ B^{2\eta }\leqslant |\textbf{x}|\leqslant B^\frac{1}{4}\\ \varDelta (\textbf{x})=\square \end{array}}\frac{\sigma _{\infty }(\textbf{x})\mathfrak {S}(\textbf{x};B^{100})}{|\textbf{x}|}=O_\eta (B^{-\eta }). \end{aligned}$$

#### Proof

To begin with, it follows from part (iv) of Lemma 7.5 that $\mathfrak {S}(\textbf{x};B^{100})=O_{\varepsilon } (B^\varepsilon )$, uniformly for $\textbf{x}\in {{\mathbb {Z}}}_{{\text {prim}}}^2$ with $|\textbf{x}|\leqslant B^\frac{1}{4}$. Moreover, (7.1) implies that $\sigma _\infty (\textbf{x})=O(1)$. Thus

$$\begin{aligned} \sum _{\begin{array}{c} \textbf{x}\in {{\mathbb {Z}}}_{{\text {prim}}}^2\\ B^{2\eta }\leqslant |\textbf{x}|\leqslant B^\frac{1}{4}\\ \varDelta (\textbf{x})=\square \end{array}}\frac{\sigma _{\infty }(\textbf{x})\mathfrak {S}(\textbf{x};B^{100})}{|\textbf{x}|} \ll _\varepsilon B^{-2\eta +\varepsilon } \#\left\{ \textbf{x}\in {{\mathbb {Z}}}_{{\text {prim}}}^2: |\textbf{x}|\leqslant B^\frac{1}{4}, ~\varDelta (\textbf{x})=\square \right\} . \end{aligned}$$

In the spirit of the proof of Proposition 5.2, the condition $\varDelta (\textbf{x})=\square$ implies that $(y,\textbf{x})$ lies on the genus one curve $y^2=\prod _{i=1}^{4}L_i(\textbf{x})$. Thus the number of $\textbf{x}\in {{\mathbb {Z}}}_{{\text {prim}}}^2$ with $|\textbf{x}|\leqslant B^\frac{1}{4}$ which verify this condition is $O_\varepsilon (B^\varepsilon )$. The lemma now follows on taking $\varepsilon =\eta /2$. $\square$

We have now come to the most difficult step in the proof of Proposition 7.2.

#### Proposition 7.9

(Second truncation of $\mathfrak {S}(\textbf{x})$) We have

$$\begin{aligned} \sum _{\begin{array}{c} \textbf{x}\in {{\mathbb {Z}}}_{{\text {prim}}}^2\\ B^{2\eta }\leqslant |\textbf{x}|\leqslant B^\frac{1}{4}\\ \varDelta _{{\text {bad}}}(\textbf{x})\leqslant B^{\eta /1000} \end{array}}\frac{\sigma _{\infty }(\textbf{x})}{|\textbf{x}|}\left( \sum _{B^{\eta /10}<q\leqslant B^{100}}\frac{S_q(\textbf{x})}{q^4}\right) =O_\eta (B^{-\eta /500}). \end{aligned}$$

This result is a direct analogue of [5, Lemma 6.6]. However, in that setting a higher power $|\textbf{x}|^3$ appears in the denominator, which has the effect of making the proof a relatively simple application of the large sieve for real characters. The proof of Proposition 7.9 is more delicate and we have divided it into several steps.

Following the template laid out to prove Proposition  5.2, we will execute a dyadic decomposition of the range of $\textbf{x}$, according to the smallest value of $|L_i(\textbf{x})|$. Since $|L_{i}(\textbf{x})|= |L_j(\textbf{x})|$ for any indices $i\ne j$ only if $\textbf{x}$ takes values in a finite set, we see that there is an overall contribution *O*(1) to the sum in the proposition from such $\textbf{x}$. This therefore allows us to partition the $\textbf{x}$-sum into four sums where $\min _{i\ne i_1}|L_i(\textbf{x})|>|L_{i_1}(\textbf{x})|,$ for $i_1\in \{1,\dots ,4\}$. We shall assume, without loss of generality, that $i_1=1$. We introduce a dyadic parameter *S* for $|\textbf{x}|$, and *R* for $|L_1(\textbf{x})|$, for $R\ll S$ and $B^{2\eta }\ll S\ll B^{\frac{1}{4}}$. Let

7.13 $$\begin{aligned} {{\mathscr {S}}}(S,R)=\left\{ \textbf{x}\in {{\mathbb {Z}}}_{{\text {prim}}}^2:|\textbf{x}|\sim S, ~|L_i(\textbf{x})|>|L_1(\textbf{x})|\sim R \text { for} i\geqslant 2\right\} . \end{aligned}$$

Then we shall be interested in bounding

$$\begin{aligned} \Sigma (S,R)= \sum _{\begin{array}{c} \textbf{x}\in {{\mathscr {S}}}(S,R)\\ \varDelta _{{\text {bad}}}(\textbf{x})\leqslant B^{\eta /1000} \end{array}}\frac{\sigma _{\infty }(\textbf{x})}{|\textbf{x}|}\left( \sum _{B^{\eta /10}<q\leqslant B^{100}}\frac{S_q(\textbf{x})}{q^4}\right) , \end{aligned}$$

for given *R*, *S* such that $R\ll S$ and $B^{2\eta }\ll S\ll B^{\frac{1}{4}}$. We shall prove the following result.

#### Lemma 7.10

Let $\eta >0$ and let $R,S\geqslant 1$ be such that $B^{2\eta }\ll S\ll B^{\frac{1}{4}}$ and $R\ll S$. Then

$$\begin{aligned} \Sigma (S,R)= O_\eta (B^{-\eta /400}). \end{aligned}$$

The statement of Proposition 7.9 is an easy consequence of this, on summing over dyadic intervals for *R* and *S*. Before proving it, we take the opportunity to record a basic estimate for the partial derivative of the real valued analytic function that weights our sum $\Sigma (S,R)$.

#### Lemma 7.11

Let $j\in \{1,2\}$ and let $K\in {\mathbb {R}}[x_1,x_2]$ be a non-zero linear form. Then the following hold:

(i) $$\begin{aligned} \frac{\partial \sigma _{\infty }(\textbf{x})}{\partial x_j} \ll |\varDelta (\textbf{x})|^{-\frac{1}{3}}(\min |L_i(\textbf{x})|)^{-\frac{2}{3}}. \end{aligned}$$
(ii) $$\begin{aligned} \frac{\partial }{\partial x_j}\frac{\sigma _{\infty }(\textbf{x})}{K(\textbf{x})}\ll _K |K(\textbf{x})|^{-1}|\varDelta (\textbf{x})|^{-\frac{1}{3}}(\min |L_i(\textbf{x})|)^{-\frac{2}{3}}+|K(\textbf{x})|^{-2}|\varDelta (\textbf{x})|^{-\frac{1}{4}}, \end{aligned}$$

#### Proof

We shall assume without loss of generality that $j=2$. We have

$$\begin{aligned} \frac{\partial }{\partial x_2}\frac{\sigma _{\infty }(\textbf{x})}{K(\textbf{x})} \ll _K\left| \frac{\partial \sigma _{\infty }(\textbf{x})}{\partial x_2}\frac{1}{K(\textbf{x})}\right| +\left| \frac{\sigma _{\infty }(\textbf{x})}{K(\textbf{x})^2}\right| . \end{aligned}$$

In view of (7.1), the second term gives rise to the second error term in part (ii) of the lemma. Thus part (ii) follows from part (i).

For any $\psi \in {{\mathbb {R}}}$, we write $I(\psi )=\int _{-1}^{1}{\text {e}}(\psi y^2){\text {d}}y$. We have $I(\psi )\ll \min (1,|\psi |^{-1/2}),$ as recorded in [5, Lemma 4.4], for example. We have

$$\begin{aligned} \sigma _{\infty }(\textbf{x})=\int _{{\mathbb {R}}}\left( \prod _{i=1}^{4}\int _{-1}^{1}{\text {e}}\left( -\theta L_i(\textbf{x})y_i^2\right) {\text {d}}y_i\right) {\text {d}}\theta =\int _{{\mathbb {R}}}\left( \prod _{i=1}^{4} I(-\theta L_i(\textbf{x}))\right) {\text {d}}\theta . \end{aligned}$$

On the other hand,

$$\begin{aligned} \frac{\partial I(-\theta L_i(\textbf{x}))}{\partial x_2}=\frac{\partial L_i(\textbf{x})}{\partial x_2}\frac{1}{2L_i(\textbf{x})}\int _{-1}^{1}y\frac{\partial }{\partial y}{\text {e}}(-\theta L_i(\textbf{x})y^2){\text {d}}y. \end{aligned}$$

The integral on the right hand side is uniformly bounded, whence

$$\begin{aligned} \frac{\partial I(-\theta L_i(\textbf{x}))}{\partial x_2}\ll |L_i(\textbf{x})|^{-1}. \end{aligned}$$

In now follows that

$$\begin{aligned} \frac{\partial \sigma _{\infty }(\textbf{x})}{\partial x_2}&\ll \sum _{i=1}^{4}|L_i(\textbf{x})|^{-1}\int _{{\mathbb {R}}}\min \left( 1,|\theta |^{-\frac{3}{2}}|\prod _{j\ne i}|L_j(\textbf{x})|^{-\frac{1}{2}}\right) {\text {d}}\theta \\&\quad \ll |\varDelta (\textbf{x})|^{-\frac{1}{3}}(\min |L_i(\textbf{x})|)^{-\frac{2}{3}}, \end{aligned}$$

which establishes part (i). $\square$

#### Proof of Lemma 7.10

Throughout the proof we may assume that the parameter $\eta >0$ is fixed but arbitrarily small. Let $\theta >\frac{3}{16}$ be a parameter to be decided upon in due course. It follows from part (iv) of Lemma 7.5 that

$$\begin{aligned} \sum _{q\leqslant B^{100}} \frac{|S_q(\textbf{x})|}{q^4}\ll _\varepsilon B^\varepsilon . \end{aligned}$$

Combining (7.1) with Lemma 5.9, we deduce that the overall contribution to $\Sigma (S,R)$ from $\textbf{x}$ such that $\varDelta _{{\text {bad}}}(\textbf{x})>(SR)^\theta$ is

$$\begin{aligned} \ll _\varepsilon \frac{B^\varepsilon (SR)^{1-\frac{\theta }{8}}}{S^{\frac{7}{4}}R^{\frac{1}{4}}}\ll B^\varepsilon S^{-\frac{\theta }{4}}\ll B^{-\frac{\theta \eta }{2}+\varepsilon }, \end{aligned}$$

since $R\ll S$ and $S\gg B^{2\eta }$. This is a satisfactory contribution since $\theta >\frac{3}{16}$.

Using the multiplicativity of $S_q(\textbf{x})$ in *q*, it follows from part (ii) of Lemma 7.5 that we may proceed under the assumption that

$$\begin{aligned} \Sigma (S,R)= \sum _{\begin{array}{c} \textbf{x}\in {{\mathscr {S}}}(S,R)\\ \varDelta _{{\text {bad}}}(\textbf{x})\leqslant \Theta \end{array}} \frac{\sigma _{\infty }(\textbf{x})}{|\textbf{x}|}\sum _{\begin{array}{c} q_2\leqslant B^{100}\\ q_2\mid (2\varDelta _{{\text {bad}}}(\textbf{x}))^\infty \end{array}}\frac{S_{q_2}(\textbf{x})}{q_2^4} \hspace{-0.4cm} \sum _{\begin{array}{c} B^{\eta /10}/q_2\leqslant q_1\leqslant B^{100}/q_2\\ 2\not \mid q_1 \end{array}} \hspace{-0.3cm} \left( \frac{\varDelta (\textbf{x})}{q_1}\right) \frac{\varphi ^*(q_1)}{q_1}, \end{aligned}$$

where

$$\begin{aligned} \Theta =\min \left( B^{\eta /1000}, (SR)^{\theta }\right) . \end{aligned}$$

We will need to show that the sums over $q_1$ and $q_2$ can be truncated satisfactorily. The inner sum over $q_1$ is $O(\log B)$. Hence part (i) of Lemma 7.5 and (7.1) implies that the contribution from $q_2>B^{\eta /100}$ is

$$\begin{aligned}&\ll \frac{\log B}{B^{\eta /200}} \sum _{\begin{array}{c} \textbf{x}\in {{\mathscr {S}}}(S,R)\\ \varDelta _{{\text {bad}}}(\textbf{x})\leqslant \Theta \end{array}}\frac{1}{|\textbf{x}||\Delta (\textbf{x})|^{\frac{1}{4}}} \#\{ q_2\leqslant B^{100}: q_2\mid (2\varDelta _{{\text {bad}}}(\textbf{x}))^\infty \}\\&\ll _\varepsilon B^{-\eta /200+\varepsilon } \frac{\#{{\mathscr {S}}}(S,R)}{S^{\frac{7}{4}}R^\frac{1}{4}}\ll _\varepsilon B^{-\eta /200+\varepsilon }, \end{aligned}$$

since $R\ll S$. Taking $\varepsilon =\eta /400$, this is satisfactory for Lemma 7.10. Next, we put

7.14 $$\begin{aligned} N_1=(S^3 R)^{2\theta }B^{\eta /50}. \end{aligned}$$

Then it follows from the Burgess bound, in the form of Lemma  3.1, that

$$\begin{aligned} \sum _{\begin{array}{c} N_1<q_1\leqslant B^{100}/q_2\\ 2\not \mid q_1 \end{array}}\left( \frac{\varDelta (\textbf{x})}{q_1}\right) \frac{\varphi ^*(q_1)}{q_1} \ll _\theta N_1^{-\frac{1}{2}}|\varDelta (\textbf{x})|^\theta \ll B^{-\eta /100}, \end{aligned}$$

since $\theta >\frac{3}{16}$. Since the contribution from the $q_2$ sum is $O_\varepsilon (B^\varepsilon )$, we obtain the overall contribution

$$\begin{aligned} \ll _\theta B^{-\eta /100+\varepsilon }\frac{\#{{\mathscr {S}}}(S,R)}{S^{\frac{7}{4}}R^\frac{1}{4}}\ll B^{-\eta /100+\varepsilon }. \end{aligned}$$

This is satisfactory for Lemma 7.10 on taking $\varepsilon =\eta /200$.

In summary, it suffices to proceed under the assumption that

$$\begin{aligned} \Sigma (S,R)= \sum _{\begin{array}{c} \textbf{x}\in {{\mathscr {S}}}(S,R)\\ \varDelta _{{\text {bad}}}(\textbf{x})\leqslant \Theta \end{array}}\frac{\sigma _{\infty }(\textbf{x})}{|\textbf{x}|}\sum _{\begin{array}{c} q_2\leqslant B^{\eta /100}\\ q_2\mid (2\varDelta _{{\text {bad}}}(\textbf{x}))^\infty \end{array}}\frac{S_{q_2}(\textbf{x})}{q_2^4}\sum _{\begin{array}{c} q_1\in I_{q_2}\cap {\mathbb {Z}}\\ 2\not \mid q_1 \end{array}}\left( \frac{\varDelta (\textbf{x})}{q_1}\right) \frac{\varphi ^*(q_1)}{q_1}, \end{aligned}$$

where

7.15 $$\begin{aligned} I_{q_2}=\left[ \frac{B^{\eta /10}}{q_2}~,~ \min \left( N_1,\frac{B^{100}}{q_2}\right) \right] . \end{aligned}$$

Next, we sort this sum according to the value of $\varDelta _{{\text {bad}}}(\textbf{x})$. The idea is now to bring the $\textbf{x}$-sum to the inside, in order to exploit cancellation from the Jacobi symbol. Thus

7.16 $$\begin{aligned} \Sigma (S,R)= \sum _{\begin{array}{c} r\leqslant \Theta \\ r \text { squarefull} \end{array}} \Sigma _r(S,R), \end{aligned}$$

where

$$\begin{aligned} \Sigma _r(S,R)= \sum _{\begin{array}{c} \textbf{x}\in {{\mathscr {S}}}(S,R)\\ \varDelta _{{\text {bad}}}(\textbf{x})=r \end{array}}\frac{\sigma _{\infty }(\textbf{x})}{|\textbf{x}|}\sum _{\begin{array}{c} q_2\leqslant B^{\eta /100}\\ q_2\mid (2r)^\infty \end{array}}\frac{S_{q_2}(\textbf{x})}{q_2^4}\sum _{\begin{array}{c} q_1\in I_{q_2}\cap {\mathbb {Z}}\\ \gcd (q_1,2r)=1 \end{array}}\left( \frac{\varDelta (\textbf{x})}{q_1}\right) \frac{\varphi ^*(q_1)}{q_1}.\nonumber \\ \end{aligned}$$

Next, we exchange the $q_2$-sum and the $\textbf{x}$-sum, by sorting the $\textbf{x}$-sum into residue classes modulo $q_2$. This leads to the expression

7.17 $$\begin{aligned} \Sigma _r(S,R)= \sum _{\begin{array}{c} q_2\leqslant B^{\eta /100}\\ q_2\mid (2r)^\infty \end{array}}\frac{1}{q_2^4} \sum _{\begin{array}{c} {\textbf{c}}\in ({{\mathbb {Z}}}/q_2{{\mathbb {Z}}})^2\\ \gcd (q_2,{\textbf{c}})=1 \end{array}}S_{q_2}({\textbf{c}}) \sum _{\begin{array}{c} q_1\in I_{q_2}\cap {\mathbb {Z}}\\ \gcd (q_1,2r)=1 \end{array}} \frac{\varphi ^*(q_1)}{q_1} \cdot U(q_1,q_2;{\textbf{c}}),\nonumber \\ \end{aligned}$$

where

$$\begin{aligned} U(q_1,q_2;{\textbf{c}})= \sum _{\begin{array}{c} \textbf{x}\in {{\mathscr {S}}}(S,R)\\ \varDelta _{{\text {bad}}}(\textbf{x})=r\\ \textbf{x}\equiv {\textbf{c}}\bmod {q_2} \end{array}}\frac{\sigma _{\infty }(\textbf{x})}{|\textbf{x}|}\left( \frac{\varDelta (\textbf{x})}{q_1}\right) . \end{aligned}$$

To handle the condition $\varDelta _{{\text {bad}}}(\textbf{x})=r$, we note that it is equivalent to the pair of conditions $\gcd \left( r,\varDelta (\textbf{x})/r\right) =1$ and $\mu ^2\left( \varDelta (\textbf{x})/r\right) =1$. These can both be detected using the Möbius function, leading to

$$\begin{aligned} U(q_1,q_2;{\textbf{c}}) =\sum _{d_1\mid r}\mu (d_1)\sum _{d_2}\mu (d_2) \sum _{\begin{array}{c} \textbf{x}\in {{\mathscr {S}}}(S,R)\\ r[d_1,d_2^2]\mid \varDelta (\textbf{x})\\ \textbf{x}\equiv {\textbf{c}}\bmod {q_2} \end{array}}\frac{\sigma _{\infty }(\textbf{x})}{|\textbf{x}|}\left( \frac{\varDelta (\textbf{x})}{q_1}\right) . \end{aligned}$$

Note $\gcd (q_1,d_1)=1$, since $\gcd (q_1,r)=1$. Hence we have $\gcd (q_1,rd_1d_2)=1$.

Clearly $\varDelta _{{\text {bad}}}(\textbf{x})\geqslant d_2^2$ and so we must have $d_2\leqslant \Theta ^{1/2}\leqslant (SR)^{\theta /2}$. We therefore have

$$\begin{aligned} U(q_1,q_2;{\textbf{c}}) =\sum _{d_1\mid r}\mu (d_1)\sum _{\begin{array}{c} d_2\leqslant (SR)^{\eta /20}\\ \gcd (d_2,q_1)=1 \end{array}}\mu (d_2) \sum _{\begin{array}{c} \textbf{x}\in {{\mathscr {S}}}(S,R)\\ r[d_1,d_2^2]\mid \varDelta (\textbf{x})\\ \textbf{x}\equiv {\textbf{c}}\bmod {q_2} \end{array}}\frac{\sigma _{\infty }(\textbf{x})}{|\textbf{x}|}\left( \frac{\varDelta (\textbf{x})}{q_1}\right) , \end{aligned}$$

where we recall that $${{\mathscr {S}}}(S,R)$$ is defined in (7.13). Since $$\textbf{x}$$ is primitive in the inner sum, it follows from (5.9) that $$\gcd (L_i(\textbf{x}),L_j(\textbf{x}))\mid {{\mathscr {D}}}$$ for $$i\ne j$$, where $${{\mathscr {D}}}$$ is defined in (5.8) and satisfies $${{\mathscr {D}}}=O(1)$$. We write $$ r[d_1,d_2^2]=DE, $$ where *D* only contains primes $$p\not \mid {{\mathscr {D}}}$$, while $$p\mid E \Rightarrow p\mid {{\mathscr {D}}}$$. We further break the $$\textbf{x}$$-sum into congruences modulo *E*, finding that

$$\begin{aligned} \sum _{\begin{array}{c} \textbf{x}\in {{\mathscr {S}}}(S,R)\\ r[d_1,d_2^2]\mid \varDelta (\textbf{x})\\ \textbf{x}\equiv {\textbf{c}}\bmod {q_2} \end{array}}\frac{\sigma _{\infty }(\textbf{x})}{|\textbf{x}|}\left( \frac{\varDelta (\textbf{x})}{q_1}\right) = \sum _{\begin{array}{c} \textbf{s}\bmod {E}\\ \gcd (\textbf{s},E)=1\\ E\mid \Delta (\textbf{s}) \end{array}} \sum _{\begin{array}{c} \textbf{x}\in {{\mathscr {S}}}(S,R)\\ D\mid \varDelta (\textbf{x})\\ \textbf{x}\equiv {\textbf{c}}\bmod {q_2}\\ \textbf{x}\equiv \textbf{s} \bmod {E} \end{array}}\frac{\sigma _{\infty }(\textbf{x})}{|\textbf{x}|}\left( \frac{\varDelta (\textbf{x})}{q_1}\right) . \end{aligned}$$

We claim that

7.18 $$\begin{aligned} \#\left\{ \textbf{s}\in ({\mathbb {Z}}/E{\mathbb {Z}})^2: \gcd (\textbf{s},E)=1, ~E\mid \Delta (\textbf{s})\right\} =O_\varepsilon (E^{1+\varepsilon }), \end{aligned}$$

for any $\varepsilon >0$. By the Chinese remainder theorem it suffices to study the case where $E=p^e$ is a prime power. If $p^\lambda \mid \gcd (L_i(\textbf{s}),L_j(\textbf{s}))$ for $i\ne j$, then $p^\lambda \mid {\mathscr {D}}$. Thus the number of solutions modulo $p^\lambda$ is clearly $O(p^\lambda )$. The claimed bound (7.18) easily follows.

Since $\gcd (D,{{\mathscr {D}}})=1$, there is a bijection between $D\mid \Delta (\textbf{x})$ and vectors $(D_1,\dots ,D_4)\in {\mathbb {N}}^4$ with pairwise coprime coordinates, such that $D_i\mid L_i(\textbf{x})$, for $1\leqslant i\leqslant 4$. Thus

$$\begin{aligned} \sum _{\begin{array}{c} \textbf{x}\in {{\mathscr {S}}}(S,R)\\ D\mid \varDelta (\textbf{x})\\ \textbf{x}\equiv {\textbf{c}}\bmod {q_2}\\ \textbf{x}\equiv \textbf{s} \bmod {E} \end{array}}\frac{\sigma _{\infty }(\textbf{x})}{|\textbf{x}|}\left( \frac{\varDelta (\textbf{x})}{q_1}\right) = \sum _{\begin{array}{c} D=D_1\cdots D_4\\ \gcd (D_i,D_j)=1 \end{array}} \sum _{\begin{array}{c} \textbf{x}\in {{\mathscr {S}}}(S,R)\\ D_i\mid L_i(\textbf{x})\\ \textbf{x}\equiv {\textbf{c}}\bmod {q_2}\\ \textbf{x}\equiv \textbf{s} \bmod {E} \end{array}}\frac{\sigma _{\infty }(\textbf{x})}{|\textbf{x}|}\left( \frac{\varDelta (\textbf{x})}{q_1}\right) . \end{aligned}$$

We use the Möbius function to remove the coprimality condition on $\textbf{x}$, and we observe that $\sigma _\infty (k\textbf{x})=k^{-1}\sigma _\infty (\textbf{x})$ for any $k>0$. Thus

7.19 $$\begin{aligned} \begin{aligned} U(q_1,q_2;{\textbf{c}})&=\sum _{d_1\mid r}\mu (d_1) \sum _{\begin{array}{c} d_2\leqslant (SR)^{\eta /20}\\ \gcd (d_2,q_1)=1 \end{array}}\mu (d_2) \hspace{-0.3cm} \sum _{r[d_1,d_2^2]=DE} \sum _{\begin{array}{c} \textbf{s}\bmod {E}\\ \gcd (\textbf{s},E)=1\\ E\mid \Delta (\textbf{s}) \end{array}}\\&\quad \sum _{\begin{array}{c} \textbf{D}\in {\mathbb {N}}^4\\ D_1\cdots D_4=D\\ \gcd (D_i,D_j)=1 \end{array}} \sum _{\begin{array}{c} k\ll R\\ \gcd (k,q_2)=1 \end{array} } \frac{\mu (k)}{k^2} U_{\textbf{D}',k}, \end{aligned} \end{aligned}$$

where

$$\begin{aligned} U_{\textbf{D}',k}= \sum _{\begin{array}{c} \textbf{x}\in {\mathbb {Z}}^2\\ |\textbf{x}|\sim S', ~|L_1(\textbf{x})|\sim R'\\ |L_i(\textbf{x})|>|L_1(\textbf{x})| \text { for} i\geqslant 2\\ D_i'\mid L_i(\textbf{x})\\ k\textbf{x}\equiv {\textbf{c}}\bmod {q_2}\\ k\textbf{x}\equiv \textbf{s} \bmod {E} \end{array}}\frac{\sigma _{\infty }(\textbf{x})}{|\textbf{x}|}\left( \frac{\varDelta (\textbf{x})}{q_1}\right) , \end{aligned}$$

with

$$\begin{aligned} D_i'=\frac{D_i}{\gcd (D_i,k)} \ \text {for}\ 1\leqslant i\leqslant 4, \quad S'=\frac{S}{k}, \quad R'=\frac{R}{k}. \end{aligned}$$

In particular, we clearly have $\gcd (D_i',D_j')=1$ for $i\ne j$ and, moreover, *k* is coprime to $q_2E$, since ${\textbf{c}}$ is coprime to $q_2$ and $\textbf{s}$ is coprime to *E*.

We now focus our attention on the sum $$U_{\textbf{D}',k}$$. Suppose that $$ L_1(x_1,x_2)=a_1x_1+b_1x_2, $$ for coprime integers $$a_1,b_1$$. Then there exists $$\textbf{M}\in \textrm{SL}_2({\mathbb {Z}})$$ with first row equal to $$(a_1,b_1)$$. Making the change of variables $$\textbf{y}=\textbf{M}\textbf{x}$$, we let $$J_i(\textbf{y})=L_i(\textbf{M}^{-1}\textbf{y})$$, for $$1\leqslant i\leqslant 4$$, and $$\varDelta ^\prime (\textbf{y})=J_1(\textbf{y})\cdots J_4(\textbf{y})$$. Under this transformation, there exists $$\textbf{c}'\in {\mathbb {Z}}^2$$ such that

$$\begin{aligned} U_{\textbf{D}',k} = \sum _{\begin{array}{c} \textbf{y}\in {\mathbb {Z}}^2\cap {\mathscr {R}}\\ D_i'\mid J_i(\textbf{y})\\ \textbf{y}\equiv \textbf{c}'\bmod {[q_2,E]}\\ \end{array}}\frac{\sigma _{\infty }'(\textbf{y})}{|\textbf{M}^{-1}\textbf{y}|}\left( \frac{\varDelta '(\textbf{y})}{q_1}\right) , \end{aligned}$$

where $$\sigma _{\infty }^\prime (\textbf{y})=\sigma _{\infty }(\textbf{M}^{-1}\textbf{y})$$ and

$$\begin{aligned} {\mathscr {R}} =\left\{ \textbf{y}\in {\mathbb {R}}^2: |y_1|\sim R', ~|\textbf{M}^{-1}\textbf{y}|\sim S',\ \text {and}\ |J_i(\textbf{y})|>|y_1|\ \text {for}\ i\geqslant 2\right\} . \end{aligned}$$

Note that once $y_1$ is fixed, there exists an interval $K_{y_1}$ of length $O( S')$, such that $\textbf{y}\in {\mathscr {R}}$ if and only if $y_2\in K_{y_1}$. Hence

7.20 $$\begin{aligned} U_{\textbf{D}',k} = \sum _{\begin{array}{c} |y_1|\sim R'\\ y_1\equiv c_1' \bmod {[q_2,E]} \end{array}} V(y_1), \end{aligned}$$

where

$$\begin{aligned} V(y_1)= \sum _{\begin{array}{c} y_2\in K_{y_1}\cap {\mathbb {Z}}\\ D_i'\mid J_i(\textbf{y})\\ y_2\equiv c_2' \bmod {[q_2,E]} \end{array}} \frac{\sigma _{\infty }'(\textbf{y})}{|\textbf{M}^{-1}\textbf{y}|}\left( \frac{\varDelta '(\textbf{y})}{q_1}\right) . \end{aligned}$$

We now seek to apply Lemma 3.2 to estimate $V(y_1)$. For this we recall that $\gcd (q_1,q_2D'E)=1$, where $D'=D_1'\cdots D_4'$. There exists a unique factorisation $q_1=ut^2$, where *u* is the largest square-free divisor of $q_1$. We then deduce from Lemma  3.2 that

$$\begin{aligned} \sum _{\begin{array}{c} y_2\in I\cap {\mathbb {Z}}\\ D_i'\mid J_i(\textbf{y})\\ y_2\equiv c_2' \bmod {[q_2,E]} \end{array}} \left( \frac{\varDelta '(\textbf{y})}{q_1}\right) \ll _\varepsilon \left( \frac{{{\,\textrm{vol}\,}}(I)}{u^{\frac{1}{2}}[[q_2,E], D']} + u^{\frac{1}{2}} \log (q_2D'E) \right) u^{\varepsilon }\gcd (y_1,u D'), \end{aligned}$$

for any $\varepsilon >0$ and any interval $I\subset {\mathbb {R}}$. Note that $[[q_2,E], D']\geqslant [E,D']=D'E,$ since $D'$ and *E* are coprime.

Armed with this bound, it now follows from Lemma  7.11 and partial summation that

$$\begin{aligned} V(y_1)&\ll \sup _{I\subset K_{y_1}}\left| \sum _{\begin{array}{c} y_2\in I\cap {\mathbb {Z}}\\ D_i' \mid J_i(\textbf{y})\\ y_2\equiv c_2'\bmod {[q_2,E]} \end{array}} \left( \frac{\varDelta ^\prime (\textbf{y})}{q_1}\right) \right| \cdot \sup _{y_2\in K_{y_1}} W(y_2), \end{aligned}$$

where

$$\begin{aligned} W(y_2)&={{\,\textrm{vol}\,}}(K_{y_1})\cdot \left| \frac{\partial }{\partial y_2}\frac{\sigma _{\infty }^\prime (\textbf{y})}{|\textbf{M}^{-1}\textbf{y}|}\right| + \frac{\sigma _{\infty }'(\textbf{y})}{|\textbf{M}^{-1}\textbf{y}|}\ll \frac{1}{R'S'}. \end{aligned}$$

Hence

$$\begin{aligned} V(y_1)&\ll _\varepsilon \left( \frac{S'}{u^{\frac{1}{2}}{D'E} } + u^{\frac{1}{2}} \log (q_2D'E) \right) \frac{ u^{\frac{\varepsilon }{2}}\gcd (y_1,u D')}{R'S'}. \end{aligned}$$

On returning to (7.20) and summing over $y_1$, we obtain

$$\begin{aligned} U_{\textbf{D}',k}&\ll _\varepsilon \frac{ u^{\frac{\varepsilon }{2}}}{R'S'} \left( \frac{S'}{u^{\frac{1}{2}}D'E} + u^{\frac{1}{2}} \log (q_2D'E) \right) \tau (uD') \left( \frac{R'}{[q_2,E]}+1\right) \\&\ll _\varepsilon \frac{ (q_1q_2DE)^{\varepsilon }}{R'S'} \left( \frac{S'}{u^{\frac{1}{2}}D'E} + u^{\frac{1}{2}} \right) \left( \frac{R'}{[q_2,E]}+1\right) . \end{aligned}$$

But

$$\begin{aligned} D'E= \frac{DE}{\gcd (D_1,k)\cdots \gcd (D_4,k)}\gg \frac{DE}{k}\geqslant \frac{d_2^2}{k}, \end{aligned}$$

since $DE=r[d_1,d_2^2]\geqslant d_2^2$. Moreover, $R'/[q_2,E]\leqslant R'=R/k$ and $S'=S/k$. It therefore follows that

$$\begin{aligned} U_{\textbf{D}',k}&\ll _\varepsilon kB^{2\varepsilon } \left( \frac{1}{u^{\frac{1}{2}}d_2^2} + \frac{u^{\frac{1}{2}}}{S} \right) , \end{aligned}$$

on noting that $q_1q_2DE\leqslant B^2.$ We now insert this into (7.19) and apply (7.18). Observe that there are $O_\varepsilon (B^\varepsilon )$ choices for $D_1,\dots ,D_4$, for fixed *D*, and that the sum over *k* contributes $O(\log B)$. Hence we find that

$$\begin{aligned} U(q_1,q_2;{\textbf{c}})&\ll _\varepsilon B^{3\varepsilon }\log B\sum _{d_1\mid r} \sum _{\begin{array}{c} d_2\leqslant (SR)^{\eta /20} \end{array}} \sum _{r[d_1,d_2^2]=DE} E^{1+\varepsilon } \left( \frac{1}{u^{\frac{1}{2}}d_2^2} + \frac{u^{\frac{1}{2}}}{S} \right) , \end{aligned}$$

where we recall that $\gcd (D,{{\mathscr {D}}})=1$ and *E* is only divisible by primes dividing ${{\mathscr {D}}}$. In particular, the factorisation of $r[d_1,d_2^2]$ as *DE* is uniquely determined. We factorise $d_2=d_2'd_2''$, where $\gcd (d_2',{{\mathscr {D}}})=1$ and $p\mid d_2'' \Rightarrow p\mid {{\mathscr {D}}}$. There are clearly $O_\varepsilon (B^\varepsilon )$ choices for $d_2''$. Moreover, we now have $E\leqslant rd_1(d_2'')^2\leqslant (rd_2'')^2$ and so we may sum over $d_2'$ and $d_2''$ to get

$$\begin{aligned} U(q_1,q_2;{\textbf{c}})&\ll _\varepsilon B^{4\varepsilon }\log B r^{2+\varepsilon } \left( \frac{1}{u^{\frac{1}{2}}} + \frac{u^{\frac{1}{2}}(SR)^{\eta /20}}{S} \right) \\&\ll _\varepsilon r^{2+\varepsilon } B^{5\varepsilon } \left( \frac{1}{u^{\frac{1}{2}}} + \frac{u^{\frac{1}{2}}(SR)^{\eta /20}}{S} \right) . \end{aligned}$$

It remains to substitute this bound into (7.17). Recalling that $q_1=ut^2$, where *u* is the largest square-free divisor of $q_1$, we observe that

$$\begin{aligned} \sum _{Q_1<q_1\leqslant Q_2} \frac{1}{q_1u^{\frac{1}{2}}}\leqslant \sum _{t\leqslant \sqrt{Q_2}} \frac{1}{t^2} \sum _{u>Q_1/t^2} \frac{1}{u^{\frac{3}{2}}}\ll Q_1^{-\frac{1}{2}}\log Q_2, \end{aligned}$$

for any $Q_1\leqslant Q_2$. Similarly

$$\begin{aligned} \sum _{Q_1<q_1\leqslant Q_2} \frac{u^{\frac{1}{2}}}{q_1}\leqslant \sum _{q_1\leqslant Q_2} q_1^{-\frac{1}{2}}\ll Q_2^{\frac{1}{2}}. \end{aligned}$$

Recalling the definitions (7.14) and (7.15) of $N_1$ and $I_{q_2}$, respectively, it follows that

$$\begin{aligned} \sum _{\begin{array}{c} q_1\in I_{q_2}\cap {\mathbb {Z}}\\ \gcd (q_1,2r)=1 \end{array}} \frac{\varphi ^*(q_1)}{q_1} \left( \frac{1}{u^{\frac{1}{2}}} + \frac{u^{\frac{1}{2}} (SR)^{\eta /20}}{S} \right)&\ll \frac{q_2^{\frac{1}{2}}\log B}{B^{\eta /20}} + \frac{N_1^{\frac{1}{2}}(SR)^{\eta /20}}{S} \\&\ll \frac{q_2^{\frac{1}{2}}\log B}{B^{\eta /20}} + \frac{B^{7\eta /100}}{S^{1-4\theta }}, \end{aligned}$$

since $R\ll S$. Appealing to part (i) of Lemma 7.5 to estimate $S_{q_2}({\textbf{c}})$ we deduce from (7.17) that

$$\begin{aligned} \Sigma _r(S,R)&\ll _\varepsilon r^{2+\varepsilon }B^{2\varepsilon }\left( \frac{1}{B^{\eta /20}} + \frac{B^{7\eta /100}}{S^{1-4\theta }}\right) \sum _{q_2\leqslant B^{\eta /100}}q_2^{2}\\&\ll _\varepsilon r^{2+\varepsilon } B^{2\varepsilon }\left( \frac{1}{B^{\eta /50}} + \frac{B^{\eta /10}}{S^{1-4\theta }}\right) . \end{aligned}$$

This bound is valid for any choice of $\theta >\frac{3}{16}$. Taking $\theta =\frac{1}{5}$ and recalling that $S\gg B^{2\eta }$, it therefore follows that

$$\begin{aligned} \Sigma _r(S,R)\ll _\varepsilon r^{2+\varepsilon } B^{2\varepsilon }\left( \frac{1}{B^{\eta /50}} + \frac{B^{9\eta /100}}{S^{1/5}}\right) \ll r^{2+\varepsilon } B^{-\eta /50+2\varepsilon }. \end{aligned}$$

Summing over $r\leqslant \Theta \leqslant B^{\eta /1000}$ in (7.16) and taking $\varepsilon$ sufficiently small, we finally conclude the proof of Lemma 7.10. $\square$

### Proof of Proposition 7.2: final step

We now have everything in place to analyse the asymptotic behaviour of *M*(*B*), as defined in (7.12). Combining Lemmas  7.6 and 7.7 with Proposition  7.9, we deduce that

$$\begin{aligned} M(B)= \sum _{\begin{array}{c} \textbf{x}\in {{\mathbb {Z}}}_{{\text {prim}}}^2\\ B^{2\eta }\leqslant |\textbf{x}|\leqslant B^\frac{1}{4}\\ \varDelta _{{\text {bad}}}(\textbf{x})\leqslant B^{\eta /1000} \end{array}}\frac{\sigma _{\infty }(\textbf{x})\mathfrak {S}(\textbf{x};B^{\eta /10})}{|\textbf{x}|} +O_\eta (1). \end{aligned}$$

The proof of Lemma 7.6 applies in the same way to show that there is an overall contribution $O_\eta (1)$ from $\textbf{x}$ such that $\varDelta _{{\text {bad}}}(\textbf{x})> B^{\eta /1000}$. Let

$$\begin{aligned} c_q(a)=\sum _{\begin{array}{c} x\bmod q\\ \gcd (x,q)=1 \end{array}} {\text {e}}_q(ax) \end{aligned}$$

be the Ramanujan sum, for $a,q\in {{\mathbb {N}}}$. Then, on opening up $\mathfrak {S}(\textbf{x};B^{\eta /10})$ and rearranging the sums, we obtain

$$\begin{aligned} M(B)= \sum _{q\leqslant B^{\eta /10}} q^{-4} \sum _{\textbf{b}\in ({\mathbb {Z}}/q{\mathbb {Z}})^4} \sum _{\begin{array}{c} \textbf{x}\in {{\mathbb {Z}}}_{{\text {prim}}}^2\\ B^{2\eta }\leqslant |\textbf{x}|\leqslant B^\frac{1}{4} \end{array}}c_q\left( \sum _{i=1}^4 L_i(\textbf{x})b_i^2\right) \frac{\sigma _{\infty }(\textbf{x})}{|\textbf{x}|} +O_\eta (1). \end{aligned}$$

We break the $\textbf{x}$-sum into residue classes modulo *q*, leading to

7.21 $$\begin{aligned} M(B)=\sum _{q\leqslant B^{\eta /10}}q^{-4} \sum _{\textbf{b}\in ({\mathbb {Z}}/q{\mathbb {Z}})^4}\sum _{\begin{array}{c} {\textbf{c}}\in ({{\mathbb {Z}}}/q{{\mathbb {Z}}})^2\\ \gcd (q,{\textbf{c}})=1 \end{array}}c_q\left( \sum _{i=1}^{4}L_i({\textbf{c}})b_i^2\right) U_q({\textbf{c}}) +O_\eta (1),\nonumber \\ \end{aligned}$$

where

$$\begin{aligned} U_q({\textbf{c}})= \sum _{\begin{array}{c} \textbf{x}\in {{\mathbb {Z}}}_{{\text {prim}}}^2\\ \textbf{x}\equiv {\textbf{c}}\bmod q\\ B^{2\eta }\leqslant |\textbf{x}|\leqslant B^\frac{1}{4} \end{array}}\frac{\sigma _{\infty }(\textbf{x})}{|\textbf{x}|}. \end{aligned}$$

The following result is concerned with the asymptotic evaluation of this sum.

#### Lemma 7.12

We have

$$\begin{aligned} U_q({\textbf{c}})= \frac{1}{q^2\zeta (2)}\prod _{p\mid q}\left( 1-\frac{1}{p^2}\right) ^{-1} \int _{\{\textbf{t}\in {\mathbb {R}}^2: B^{2\eta }\leqslant |\textbf{t}| \leqslant B^{\frac{1}{4}}\}} \frac{\sigma _\infty (\textbf{t})}{|\textbf{t}|} \textrm{d} \textbf{t} +O(B^{-\frac{6\eta }{7}}\log B), \end{aligned}$$

for any ${\textbf{c}}\in ({\mathbb {Z}}/q{\mathbb {Z}})^2$ and $q\leqslant B^{\eta /10}$.

#### Proof

It will be convenient to define $m(\textbf{x})=\min _{1\leqslant i\leqslant 4}|L_i(\textbf{x})|$ in the proof of this result. Then, in view of Lemma  5.7 and (7.1), we have the estimate

7.22 $$\begin{aligned} \sigma _\infty (\textbf{x})\ll |\textbf{x}|^{-\frac{3}{4}}m(\textbf{x})^{-\frac{1}{4}}. \end{aligned}$$

Since there are no primitive vectors $\textbf{x}\in {\mathbb {Z}}^2$ with $|x_1|=|x_2|$ and $|\textbf{x}|>B^{2\eta }$, we may write

$$\begin{aligned} U_q({\textbf{c}})= \sum _{\begin{array}{c} \textbf{x}\in {{\mathbb {Z}}}_{{\text {prim}}}^2,~ |x_1|<|x_2|\\ \textbf{x}\equiv {\textbf{c}}\bmod q\\ B^{2\eta }\leqslant |x_2|\leqslant B^\frac{1}{4} \end{array}}\frac{\sigma _{\infty }(\textbf{x})}{|x_2|}+ \sum _{\begin{array}{c} \textbf{x}\in {{\mathbb {Z}}}_{{\text {prim}}}^2,~ |x_2|<|x_1|\\ \textbf{x}\equiv {\textbf{c}}\bmod q\\ B^{2\eta }\leqslant |x_1|\leqslant B^\frac{1}{4} \end{array}}\frac{\sigma _{\infty }(\textbf{x})}{|x_1|}=U_q^{(1)}({\textbf{c}})+U_q^{(2)}({\textbf{c}}), \end{aligned}$$

say. We focus our efforts on $U_q^{(1)}({\textbf{c}})$, the treatment of the remaining sum being identical.

We begin by handling the overall contribution to $U_q^{(1)}({\textbf{c}})$ from $\textbf{x}$ such that $m(\textbf{x})\leqslant \delta |x_2|$, for a parameter $\delta$ that will be selected in due course, but which will tend to 0 as $B\rightarrow \infty$. In particular $m(\textbf{x})$ cannot be proportional to $x_2$ in this case. Given $x_2\in {\mathbb {Z}}$, there are at most *O*(*L*) values of $x_1\in {\mathbb {Z}}$ such that $m(\textbf{x})\leqslant L$, for any $L\leqslant \delta |x_2|$. Thus (7.22) implies that

$$\begin{aligned} \sum _{\begin{array}{c} \textbf{x}\in {{\mathbb {Z}}}_{{\text {prim}}}^2, ~|x_1|<|x_2|\\ m(\textbf{x})\leqslant \delta |x_2|\\ B^{2\eta }\leqslant |x_2|\leqslant B^\frac{1}{4} \end{array}}\frac{\sigma _{\infty }(\textbf{x})}{|x_2|}\ll \delta ^{\frac{3}{4}}\log B. \end{aligned}$$

Since $\sigma _{\infty }(k\textbf{x})=k^{-1}\sigma _{\infty }(\textbf{x})$, for any $k>0$, we apply Möbius inversion to deal with the coprimality of $\textbf{x}$, giving

$$\begin{aligned} U_q^{(1)}({\textbf{c}})=\sum _{\begin{array}{c} k\leqslant B^{\frac{1}{4}}\\ \gcd (k,q)=1 \end{array}} \frac{\mu (k)}{k^2} \sum _{\begin{array}{c} \textbf{x}\in {\mathbb {Z}}^2,~|x_1|<|x_2|\\ \textbf{x}\equiv \bar{k}{\textbf{c}}\bmod q\\ B^{2\eta }\leqslant k|x_2|\leqslant B^\frac{1}{4}\\ m(\textbf{x})>\delta |x_2| \end{array}}\frac{\sigma _{\infty }(\textbf{x})}{|x_2|} +O\left( \delta ^{\frac{3}{4}}\log B\right) . \end{aligned}$$

where ${\bar{k}}$ is the multiplicative inverse of *k* modulo *q*. It follows from (7.22) that the $\textbf{x}$-sum is $O(\log B)$. Hence, the overall contribution to the main term from $k>B^{2\eta }$ is easily seen to be $O(B^{-2\eta }\log B)$. Hence

$$\begin{aligned} U_q^{(1)}({\textbf{c}})=\sum _{\begin{array}{c} k\leqslant B^{2 \eta }\\ \gcd (k,q)=1 \end{array}} \frac{\mu (k)}{k^2} \hspace{-0.3cm} \sum _{\begin{array}{c} x_2\in {\mathbb {Z}}\\ x_2\equiv \bar{k} c_2\bmod {q}\\ B^{2\eta }/k\leqslant |x_2|\leqslant B^\frac{1}{4}/k \end{array}} \frac{1}{|x_2|} \sum _{\begin{array}{c} x_1\in {\mathbb {Z}}\cap K_{x_2}\\ x_1\equiv \bar{k}c_1\bmod q \end{array}}\sigma _{\infty }(\textbf{x}) +O((B^{-2 \eta }+\delta ^{\frac{3}{4}})\log B), \end{aligned}$$

where $K_{x_2}$ is the interval of $t\in {\mathbb {R}}$ such that $|t|<|x_2|$ and $m(t,x_2)>\delta |x_2|$.

Appealing to partial summation, together with (7.22) and part (i) of Lemma 7.11, it easily follows that

$$\begin{aligned} \sum _{\begin{array}{c} x_1\in {\mathbb {Z}}\cap K_{x_2}\\ x_1\equiv \bar{k}c_1\bmod q \end{array}}\sigma _{\infty }(\textbf{x}) =\frac{1}{q} \int _{K_{x_2}} \sigma _\infty (t_1,x_2)\textrm{d} t_1 +O\left( E_1(x_2) \right) , \end{aligned}$$

where

$$\begin{aligned} E_1(x_2)&= \sup _{t\in K_{x_2}}|\Delta (t,x_2)|^{-\frac{1}{4}}+ \int _{K_{x_2}} \left( |\Delta (t,x_2)|^{-\frac{1}{3}}m(t,x_2)^{-\frac{2}{3}}\right) \textrm{d} t \ll \frac{1}{|x_2| \delta }. \end{aligned}$$

For given $t_1$, let $I_{t_1}$ be the interval of $t\in {\mathbb {R}}$ cut out by the conditions $m(t_1,t)>\delta |t|$, $B^{2\eta }/k\leqslant |t|\leqslant B^\frac{1}{4}/k$ and $|t|> |t_1|$. We therefore obtain

7.23 $$\begin{aligned} U_q^{(1)}({\textbf{c}})=\frac{1}{q}\sum _{\begin{array}{c} k\leqslant B^{2\eta }\\ \gcd (k,q)=1 \end{array}} \frac{\mu (k)}{k^2} \int _{-B^{\frac{1}{4}}/k}^{B^{\frac{1}{4}}/k} S(t_1) \textrm{d} t_1 +O((B^{-2 \eta }+\delta ^{\frac{3}{4}})\log B), \end{aligned}$$

where

$$\begin{aligned} S(t_1)= \sum _{\begin{array}{c} x_2\in {\mathbb {Z}}\cap I_{t_1}\\ x_2\equiv \bar{k} c_2\bmod {q} \end{array}} \frac{\sigma _\infty (t_1,x_2)}{|x_2|}. \end{aligned}$$

We now once more use partial summation, equipped with (7.22) and part (ii) of Lemma 7.11. This leads to the conclusion that

$$\begin{aligned} S(t_1) =\frac{1}{q} \int _{I_{t_1}}\frac{\sigma _\infty (t_1,t_2)}{|t_2|}\textrm{d} t_2 +O\left( E_2(t_1) \right) , \end{aligned}$$

where

$$\begin{aligned} E_2(t_1)&= \sup _{t\in I_{t_1}}\frac{|\Delta (t_1,t)|^{-\frac{1}{4}}}{|t|} +\int _{I_{t_1}} \left( \frac{|\Delta (t_1,t)|^{-\frac{1}{3}}m(t_1,t)^{-\frac{2}{3}}}{|t|} +\frac{|\Delta (t_1,t)|^{-\frac{1}{4}}}{|t|^2} \right) \textrm{d} t\\&\ll \frac{1}{\max (B^{2\eta }/k, |t_1|)^2\delta }. \end{aligned}$$

Moreover, it is easily confirmed that

$$\begin{aligned} \int _{I_{t_1}}\frac{\sigma _\infty (t_1,t_2)}{|t_2|}\textrm{d} t_2 = \int _{J_{t_1}}\frac{\sigma _\infty (t_1,t_2)}{|t_2|}\textrm{d} t_2 +O\left( \frac{\delta ^{\frac{3}{4}}}{ \max (B^{2\eta }/k, |t_1|)} \right) , \end{aligned}$$

where $J_{t_1}$ is defined as for $I_{t_1}$, but with the constraint $m(t_1,t_2)>\delta |t_2|$ removed. Hence it follows that

$$\begin{aligned} S(t_1) =\frac{1}{q} \int _{J_{t_1}}\frac{\sigma _\infty (t_1,t_2)}{|t_2|}\textrm{d} t_2 +O\left( \frac{1}{\max (B^{2\eta }/k, |t_1|)^2\delta }+ \frac{\delta ^{\frac{3}{4}}}{ \max (B^{2\eta }/k, |t_1|)}\right) . \end{aligned}$$

The contribution from this error term to (7.23) is

$$\begin{aligned}&\ll \frac{1}{q} \sum _{k\leqslant B^{2 \eta }} \frac{1}{k^2} \left( \frac{k}{B^{2\eta }\delta }+ \delta ^{\frac{3}{4}} \log B \right) \ll \left( \frac{1}{B^{2\eta }\delta }+ \delta ^{\frac{3}{4}} \right) \log B. \end{aligned}$$

An obvious change of variables shows that

$$\begin{aligned} \int _{-B^{\frac{1}{4}}/k}^{B^{\frac{1}{4}}/k} \int _{J_{t_1}}\frac{\sigma _\infty (t_1,t_2)}{|t_2|}\textrm{d} t_2 \textrm{d} t_1 = \int _{\{\begin{array}{c} \textbf{t}\in {\mathbb {R}}^2: B^{2\eta }\leqslant |t_2| \leqslant B^{\frac{1}{4}} \end{array}, ~|t_2|>|t_1|\}} \frac{\sigma _\infty (\textbf{t})}{|t_2|} \textrm{d} \textbf{t}. \end{aligned}$$

Hence, on returning to (7.23) and extending the *k*-sum to infinity, we readily obtain

$$\begin{aligned} U_q^{(1)}({\textbf{c}})=~&\frac{1}{q^2}\sum _{\begin{array}{c} k=1\\ \gcd (k,q)=1 \end{array}}^\infty \frac{\mu (k)}{k^2} \int _{\{\begin{array}{c} \textbf{t}\in {\mathbb {R}}^2: B^{2\eta }\leqslant |t_2| \leqslant B^{\frac{1}{4}} \end{array}, ~|t_2|>|t_1|\}} \hspace{-0.3cm} \frac{\sigma _\infty (\textbf{t})}{|t_2|} \textrm{d} \textbf{t}\\&\quad +O\left( (B^{-2 \eta }\delta ^{-1}+\delta ^{\frac{3}{4}})\log B\right) . \end{aligned}$$

Clearly

$$\begin{aligned} \sum _{\begin{array}{c} k=1\\ \gcd (k,q)=1 \end{array}}^\infty \frac{\mu (k)}{k^2} =\frac{1}{\zeta (2)}\prod _{p\mid q}\left( 1-\frac{1}{p^2}\right) ^{-1}. \end{aligned}$$

The statement of the lemma is now a consequence of combing this with the analogous estimate for $U_q^{(2)}({\textbf{c}})$, which follows by symmetry, and taking $\delta =B^{-\frac{8\eta }{7}}$. $\square$

Before returning to our expression (7.21) for *M*(*B*), we proceed by analysing the term

$$\begin{aligned} \int _{\{\textbf{t}\in {\mathbb {R}}^2: B^{2\eta }\leqslant |\textbf{t}| \leqslant B^{\frac{1}{4}}\}} \frac{\sigma _\infty (\textbf{t})}{|\textbf{t}|} \textrm{d} \textbf{t}= \int _{\{\textbf{t}\in {\mathbb {R}}^2: 1\leqslant |\textbf{t}| \leqslant B^{\frac{1}{4}}\}} \frac{\sigma _\infty (\textbf{t})}{|\textbf{t}|} \textrm{d} \textbf{t}+O(\eta \log B). \end{aligned}$$

Arguing as in the proof of [5, Lemma 6.4], it easily follows that

$$\begin{aligned} \int _{\{\textbf{t}\in {\mathbb {R}}^2: 1\leqslant |\textbf{t}| \leqslant B^{\frac{1}{4}}\}} \frac{\sigma _\infty (\textbf{t})}{|\textbf{t}|} \textrm{d} \textbf{t}=\frac{1}{4}\tau _\infty \log B, \end{aligned}$$

where $\tau _\infty$ is defined in (2.9). Note that, as readily follows from (7.1), we have

7.24 $$\begin{aligned} \tau _\infty =O(1), \end{aligned}$$

for an implied constant that depends on $L_1,\dots ,L_4$.

In summary, it follows from combining the previous calculation with (7.21) and Lemma 7.12 that

7.25 $$\begin{aligned} M(B)= \frac{1}{\zeta (2)}\left( \sum _{q\leqslant B^{\eta /10}} \frac{A_q}{q^6}\prod _{p\mid q}\left( 1-\frac{1}{p^2}\right) ^{-1} \right) \left( \frac{\tau _\infty }{4} +O(\eta )\right) \log B +O_\eta (1),\nonumber \\ \end{aligned}$$

where $A_q$ is given by (2.10). The final remaining task is to show that the sum over *q* can be extended to infinity, with acceptable error. Since $A_q$ is multiplicative in *q*, it will suffice to study it when *q* is prime power, as in the following result.

#### Lemma 7.13

For any $r\in {{\mathbb {N}}}$ and any prime *p*, we have

$$\begin{aligned} A_{p^r}=\varphi (p^r)p^{2r}\left( \varrho (p^r)-p^2\varrho (p^{r-1})\right) , \end{aligned}$$

where $\varrho$ is defined in (3.6).

#### Proof

We begin by observing that

$$\begin{aligned} \sum _{\begin{array}{c} {\textbf{c}}\in ({{\mathbb {Z}}}/p^r{{\mathbb {Z}}})^2 \end{array}}c_{p^r}\left( \sum _{i=1}^{4}L_i({\textbf{c}})b_i^2\right)&= \sum _{\begin{array}{c} a\bmod {p^r}\\ \gcd (a,p)=1 \end{array}} \sum _{\begin{array}{c} {\textbf{c}}\in ({{\mathbb {Z}}}/p^r{{\mathbb {Z}}})^2 \end{array}} {\text {e}}_{p^r}\left( a(c_1Q_1({\textbf{b}})-c_2Q_2({\textbf{b}}))\right) \\&= {\left\{ \begin{array}{ll} \varphi (p^r)p^{2r} &{}\text {if}\ p^r\mid (Q_1({\textbf{b}}),Q_2({\textbf{b}})),\\ 0 &{}\text { otherwise}. \end{array}\right. } \end{aligned}$$

On noting that $A_{p^r}$ can be written as the difference of sums

$$\begin{aligned} \sum _{\textbf{b}\in ({\mathbb {Z}}/p^r{\mathbb {Z}})^4}\left( \sum _{\begin{array}{c} {\textbf{c}}\in ({{\mathbb {Z}}}/p^r{{\mathbb {Z}}})^2 \end{array}}c_{p^r}\left( \sum _{i=1}^{4}L_i({\textbf{c}})b_i^2\right) - p \sum _{\begin{array}{c} {\textbf{c}}\in ({{\mathbb {Z}}}/p^{r-1}{{\mathbb {Z}}})^2 \end{array}}c_{p^{r-1}}\left( \sum _{i=1}^{4}L_i({\textbf{c}})b_i^2\right) \right) , \end{aligned}$$

the lemma readily follows. $\square$

#### Corollary 7.14

Let $\varepsilon >0$ and let $q=q_0q_1$, where $\gcd (q_0,\Delta )=1$ and $q_1\mid \Delta ^\infty$. Then $A_q\ll _\varepsilon q_0^{\frac{9}{2}+\varepsilon } q_1^{5+\varepsilon }.$

#### Proof

We have $A_q=A_{q_0}A_{q_1}$. Now it follows from part (i) of Lemma  7.5 and (7.18) that

$$\begin{aligned} A_{q_1}=\sum _{\begin{array}{c} \textbf{c}\in ({\mathbb {Z}}/q_1{\mathbb {Z}})^2\\ \gcd (q_1,\textbf{c})=1 \end{array}} S_{q_1}({\textbf{c}})\ll q_1^3 \sum _{\begin{array}{c} \textbf{c}\in ({\mathbb {Z}}/q_1{\mathbb {Z}})^2\\ \gcd (q_1,\textbf{c})=1 \end{array}}\gcd (q_1,\Delta ({\textbf{c}}))^{\frac{1}{2}}\ll _\varepsilon q_1^3\sum _{d\mid q_1} d^{\frac{1}{2}} \cdot \left( \frac{q_1}{d}\right) ^2 \cdot d^{1+\varepsilon }. \end{aligned}$$

Thus $A_{q_1}=O_\varepsilon (q_1^{5+\varepsilon })$ on taking the trivial estimate for the divisor function.

Turning to $A_{q_0}$, we study $A_{p^r}$ for $p\not \mid \Delta$. It follows from Lemma 7.13 that

$$\begin{aligned} |A_{p^r}|\leqslant p^{3r}\left| \varrho (p^r)-p^2\varrho (p^{r-1})\right| , \end{aligned}$$

Extracting common divisors between $\textbf{y}$ and $p^r$ it is easily checked that

$$\begin{aligned} \varrho (p^r)=\sum _{0\leqslant k< \frac{r}{2}} p^{2k} \varrho ^*(p^{r-2k}) +p^{2(r-\lceil \frac{r}{2}\rceil )}, \end{aligned}$$

in the notation of (3.7). (This follows from [6, Eq. (2.4)], for example.) Since $p\not \mid \Delta$, it follows from part (i) of Lemma 3.4 that

$$\begin{aligned} \varrho (p^r)=p^{2r}\left( 1+O(p^{-\frac{1}{2}})\right) \sum _{0\leqslant k< \frac{r}{2}} p^{-2k} +p^{2(r-\lceil \frac{r}{2}\rceil )}. \end{aligned}$$

Similarly,

$$\begin{aligned} p^2\varrho (p^{r-1})=p^{2r}\left( 1+O(p^{-\frac{1}{2}})\right) \sum _{0\leqslant k< \frac{r-1}{2}} p^{-2k} +p^{2(r-1-\lceil \frac{r-1}{2}\rceil )}. \end{aligned}$$

Combining these, it easily follows that $\varrho (p^r)-p^2\varrho (p^{r-1})\ll p^{r+1}\leqslant p^{\frac{3r}{2}},$ if $p\not \mid \Delta$. The statement of the lemma follows. $\square$

Taking $\varepsilon =\frac{1}{4}$ in Corollary 7.14, it follows that

$$\begin{aligned} \sum _{q> B^{\eta /10}} \frac{A_q}{q^6}\prod _{p\mid q}\left( 1-\frac{1}{p^2}\right) ^{-1} \ll \sum _{p\mid q_1\Rightarrow p\mid \Delta } q_1^{-\frac{3}{4}} \sum _{q_0> B^{\eta /10}/q_1} q_0^{-\frac{5}{4}}&\ll B^{-\eta /40}\sum _{p\mid q_1\Rightarrow p\mid \Delta } q_1^{-\frac{1}{2}}\\&\ll B^{-\eta /40}. \end{aligned}$$

In particular, this implies that

7.26 $$\begin{aligned} \mathfrak {S}_2=O(1), \end{aligned}$$

in the notation of (2.11). Hence, returning to (7.25), it now follows that

$$\begin{aligned} M(B)&= \frac{\mathfrak {S}_2}{\zeta (2)} \left( \frac{\tau _\infty }{4} +O(\eta )\right) \log B +O_\eta (1), \end{aligned}$$

which easily leads to the statement of Proposition 7.2.

## Comparison of the leading constants

In this section we complete the proof of Theorem 1.1. On recalling (2.6) and (2.7), we see that

$$\begin{aligned} N_V(\Omega ,B)=\frac{1}{4}\left( \#{{\mathscr {L}}}_1(B)+\#{{\mathscr {L}}}_2(B)+\#{{\mathscr {L}}}_3(B)\right) \end{aligned}$$

in (2.4). We begin by analysing the main term in Proposition 2.2.

### Lemma 8.1

Let $$Y_2>Y_1\geqslant 1$$. Then

$$\begin{aligned} \int _{\begin{array}{c} \textbf{y}\in {\mathbb {R}}^4\\ Y_1 \leqslant |\textbf{y}|< Y_2 \end{array}} \frac{\textrm{d}\textbf{y}}{|\textbf{y}|^2\max (|Q_1(\textbf{y})|,|Q_2(\textbf{y})|)} = \tau _\infty \log \left( \frac{Y_2}{Y_1}\right) , \end{aligned}$$

where $\tau _\infty$ is given by (2.9).

This result will be established at the end of this section. Taking it on faith for the moment, and arguing as [5, § 6.3], it now follows from the union of Propositions 2.2–2.4 that

$$\begin{aligned} N_V(\Omega ,B)\sim c B\log B, \end{aligned}$$

as $$B\rightarrow \infty $$, with

8.1 $$\begin{aligned} c=\frac{\tau _\infty }{4}\left( \frac{ \mathfrak {S}_1}{4}+ \frac{ \mathfrak {S}_2}{4\zeta (2)^2}\right) . \end{aligned}$$

The following result confirms that this agrees with Peyre’s constant [20], as required to complete the proof of Theorem 1.1.

### Proposition 8.2

We have $c=c_V$, where $c_V$ is the constant predicted by Peyre.

The constant $c_V$ has been calculated by Elsenhans [9], but we shall give more details here. Let $V\subset {\mathbb {P}}^1\times {\mathbb {P}}^3$ be the smooth threefold (1.3), which we view as the blow-up of ${\mathbb {P}}^3$ along the genus 1 curve *Z*. The Picard group ${{\,\textrm{Pic}\,}}(V)$ is generated by the hyperplane classes $H_1=\pi _1^*{\mathscr {O}}_{{\mathbb {P}}^1}(1)$ and $H_2=\pi _2^*{\mathscr {O}}_{{\mathbb {P}}^3}(1)$. On the other hand, we saw in §1 that the effective cone ${{\,\textrm{Eff}\,}}_V$ is generated by $H_1$ and the class of the exceptional divisor $E=-H_1+2H_2$. Finally, the class of the anticanonical divisor is $-K_V= 4H_2-H_1$. The constant $c_V$ predicted by Peyre [20] then takes the shape

$$\begin{aligned} c_V=\alpha (V) \omega _\infty (V({\mathbb {R}})) \prod _p \left( 1-\frac{1}{p}\right) ^2\omega _p(V({\mathbb {Q}}_p)), \end{aligned}$$

where

8.2 $$\begin{aligned} \alpha (V)={{\,\textrm{rank}\,}}\left( {{\,\textrm{Pic}\,}}(V)\right) \cdot {{\,\textrm{vol}\,}}\left( \textbf{x}\in {{\,\textrm{Eff}\,}}_V^\vee : \langle \textbf{x}, -K_V\rangle \leqslant 1 \right) \end{aligned}$$

and, for each place *v*, the measure $\omega _v$ is the local Tamagawa measure defined by Peyre [20]. The dual of the effective cone is ${{\,\textrm{Eff}\,}}_V^\vee = \{(t_1,t_2)\in {\mathbb {R}}^2: t_1\ge 0, 2t_2 - t_1\ge 0\}$, and so the volume in (8.2) is

$$\begin{aligned} {{\,\textrm{vol}\,}}\{(t_1,t_2)\in {\mathbb {R}}^2: t_1\ge 0, ~2t_2 - t_1\ge 0, ~4t_2 - t_1 \le 1\}. \end{aligned}$$

This is the volume of the triangle with vertices (0, 0), $(0,\frac{1}{4})$, and $(1,\frac{1}{2})$, so $\alpha (V) =2\cdot \frac{1}{8}=\frac{1}{4}$. The quantities $\omega _\infty (V({\mathbb {R}}))$ and $\omega _p(V({\mathbb {Q}}_p))$ have been calculated by Schindler [22, § 3]. It follows from [22, Lemma 3.2] that $\omega _\infty (V({\mathbb {R}}))=\frac{1}{2} \tau _\infty ,$ where $\tau _\infty$ is given by (2.9), and from [22, Lemma 3.1] that

$$\begin{aligned} \omega _p(V({\mathbb {Q}}_p))=\left( 1-\frac{1}{p}\right) ^{-2} \left( 1-\frac{1}{p}\right) \left( 1-\frac{1}{p^2}\right) \tau _p, \end{aligned}$$

where

$$\begin{aligned} \tau _p=\lim _{t\rightarrow \infty }p^{-5t}\#\left\{ (\textbf{x},\textbf{y})\in ({{\mathbb {Z}}}/p^t{{\mathbb {Z}}})^6: L_1(\textbf{x})y_1^2+\cdots + L_4(\textbf{x})y_4^2\equiv 0\bmod p^t\right\} . \end{aligned}$$

In this way, we deduce that

8.3 $$\begin{aligned} c_V=\frac{1}{8}\tau _\infty \prod _{p}(1-p^{-1})(1-p^{-2})\tau _p. \end{aligned}$$

At first glance, it is not perhaps clear that the Euler product converges in (8.3). However, Elsenhans gives an explicit formula for $\tau _p$ when $p\not \mid \Delta$ where $\Delta$ is given by (2.1). Let *E* be the elliptic curve cut out by the equation $y^2=\prod _{i=1}^4 L_i(x_1,x_2)$ in ${\mathbb {P}}(2,1,1)$. Let $T_p(E)=p+1-\#E({{\mathbb {F}}}_p)$ be the Frobenian trace of *E*. Then it follows from [9, § 3.1] that

$$\begin{aligned} \tau _p=\left( 1+\frac{1}{p}\right) ^{-1}\left( 1 +\frac{2}{p} -\frac{T_p(E)-2}{p^2}+\frac{1}{p^3}\right) . \end{aligned}$$

The Hasse–Weil bound gives $|T_p(E)|\leqslant 2\sqrt{p}$. Thus

$$\begin{aligned} \prod _{p\not \mid \Delta } (1-p^{-1})(1-p^{-2})\tau _p= \prod _{p\not \mid \Delta } \left( 1-\frac{1}{p}\right) ^2 \left( 1 +\frac{2}{p} +O\left( \frac{1}{p^{\frac{3}{2}}}\right) \right) , \end{aligned}$$

which is clearly convergent.

For any prime *p*, we may write $\tau _p=\lim _{t\rightarrow \infty } p^{-5t}n(p^t)$, where

$$\begin{aligned} n(p^t)=\#\left\{ (\textbf{x},\textbf{y})\in ({{\mathbb {Z}}}/p^t{{\mathbb {Z}}})^6: L_1(\textbf{x})y_1^2+\cdots + L_4(\textbf{x})y_4^2\equiv 0\bmod p^t\right\} . \end{aligned}$$

The following result provides a convenient formula for this quantity.

### Lemma 8.3

For any prime power $p^t$, we have

$$\begin{aligned} p^{-5t} n(p^t)=1+\left( 1-\frac{1}{p}\right) \sum _{j=1}^{t}\frac{\varrho (p^j)}{p^{3j}}, \end{aligned}$$

where $\varrho$ is defined in (3.6). In particular,

$$\begin{aligned} \tau _p=1+\left( 1-\frac{1}{p}\right) \sum _{j=1}^{\infty }\frac{\varrho (p^j)}{p^{3j}}. \end{aligned}$$

### Proof

On recalling the definition (3.6) of $\varrho$, we may write

$$\begin{aligned} n(p^t)&=\#\{(\textbf{x},\textbf{y})\in ({{\mathbb {Z}}}/p^t{{\mathbb {Z}}})^6:x_1Q_1(\textbf{y})\equiv x_2Q_2(\textbf{y})\bmod p^t\}\\&=\varrho (p^t)p^{2t}+\sum _{j=0}^{t-1}\sum _{\begin{array}{c} \textbf{y}\bmod p^t\\ p^j\Vert \gcd (Q_1(\textbf{y}),Q_2(\textbf{y})) \end{array}} \hspace{-0.4cm}\#\{\textbf{x}\bmod p^t:x_1Q_1(\textbf{y})\equiv x_2Q_2(\textbf{y})\bmod p^t\}. \end{aligned}$$

For each $0\leqslant j\leqslant t-1$ and for each $\textbf{y}$ in the sum, any $\textbf{x}\bmod p^t$ to be counted must satisfy $x_1p^{-j}{Q_1(\textbf{y})} \equiv x_2p^{-j}{Q_2(\textbf{y})}\bmod p^{t-j}.$ Since $p\not \mid p^{-j}\gcd (Q_1(\textbf{y}), Q_2(\textbf{y}))$, the number of such $\textbf{x}\bmod p^{t-j}$ is $p^{t-j}$, giving $p^{t-j}\cdot p^{2j}=p^{t+j}$ values of $\textbf{x}\bmod p^t$. Moreover,

$$\begin{aligned} \sum _{\begin{array}{c} \textbf{y}\bmod p^t\\ p^j\Vert \gcd (Q_1(\textbf{y}),Q_2(\textbf{y})) \end{array}} 1&= \sum _{\begin{array}{c} \textbf{y}\bmod p^t\\ p^j\mid \gcd (Q_1(\textbf{y}),Q_2(\textbf{y})) \end{array}}1- \sum _{\begin{array}{c} \textbf{y}\bmod p^t\\ p^{j+1}\mid \gcd (Q_1(\textbf{y}),Q_2(\textbf{y})) \end{array}}1\\&= p^{4(t-j)}\varrho (p^j)-p^{4(t-j-1)}\varrho (p^{j+1}). \end{aligned}$$

It therefore follows that

$$\begin{aligned} n(p^t)&=\varrho (p^t)p^{2t}+\sum _{j=0}^{t-1}p^{t+j}\left( p^{4(t-j)}\varrho (p^j)-p^{4(t-j-1)} \varrho (p^{j+1})\right) \\ {}&=\varrho (p^t)p^{2t}+\sum _{j=0}^{t-1}p^{5t-3j}\left( \varrho (p^j)-p^{-4}\varrho (p^{j+1})\right) , \end{aligned}$$

whence

$$\begin{aligned} \frac{n(p^t)}{p^{5t}}&=\frac{\varrho (p^t)}{p^{3t}}+\sum _{j=0}^{t-1}\left( \frac{\varrho (p^j)}{p^{3j}} -\frac{1}{p}\frac{\varrho (p^{3(j+1)})}{p^{3(j+1)}}\right) =1+\left( 1-\frac{1}{p}\right) \sum _{j=1}^{t}\frac{\varrho (p^j)}{p^{3j}}. \end{aligned}$$

as claimed. $$\square $$

We now turn to the Euler product $\mathfrak {S}_1$ defined in (2.8), writing $\mathfrak {S}_1=\prod _{p}\lambda _p$, say. The following result confirms that the local factor $\lambda _p$ matches the corresponding local factor in Peyre’s constant (8.3).

### Lemma 8.4

For any prime *p*, we have $\lambda _p= (1-p^{-1})(1-p^{-2})\tau _p.$

### Proof

Let *p* be a prime. We have

$$\begin{aligned} \lambda _p= \left( 1-\frac{1}{p}\right) \left( 1-\frac{1}{p^4}+\left( 1-\frac{1}{p}\right) ^2\sum _{a=1}^{\infty }\frac{\#V_{p^{a}}^{\times }}{p^{2a}} \right) , \end{aligned}$$

where $V_{p^a}^{\times }$ is given by (3.8). We observe that

$$\begin{aligned} \varrho ^*(p^{b})= {\left\{ \begin{array}{ll} \varrho (p)-1 &{}\text {if}\ b=1\\ \varrho (p^{b})-p^{4}\varrho (p^{a-2}) &{}\text { otherwise}. \end{array}\right. } \end{aligned}$$

Hence it follows from (3.13) that

$$\begin{aligned} \begin{aligned} \sum _{a=1}^{\infty }\frac{\#V_{p^{a}}^{\times }}{p^{2a}}&= \left( 1-\frac{1}{p}\right) ^{-1}\sum _{a=1}^{\infty }\frac{\varrho ^{*} (p^{a})}{p^{3a}}\\&=\left( 1-\frac{1}{p}\right) ^{-1}\left( \left( 1-\frac{1}{p^{2}}\right) \sum _{a=1}^{\infty }\frac{\varrho (p^{a})}{p^{3a}}-\frac{1}{p^{2}}\left( 1+\frac{1}{p}\right) \right) \\&=\left( 1+\frac{1}{p}\right) \left( \sum _{a=1}^{\infty }\frac{\varrho (p^{a})}{p^{3a}}-\frac{1}{p(p-1)}\right) . \end{aligned} \end{aligned}$$

Thus $$\lambda _p=(1-\frac{1}{p})(1-\frac{1}{p^{2}})\lambda _{p}'$$, where

$$\begin{aligned} \begin{aligned} \lambda _{p}'=1+\frac{1}{p^{2}}+\left( 1-\frac{1}{p}\right) \sum _{a=1}^{\infty }\frac{\varrho (p^{a})}{p^{3a}} -\frac{1-\frac{1}{p}}{p(p-1)}&=1+\left( 1-\frac{1}{p}\right) \sum _{a=1}^{\infty }\frac{\varrho (p^{a})}{p^{3a}}. \end{aligned} \end{aligned}$$

Lemma 8.3 confirms that the right hand side is $\tau _p$. $\square$

It remains to examine the second term in (8.1). We recall that

$$\begin{aligned} \mathfrak {S}_2=\sum _{q=1}^\infty \frac{A_q}{q^6}\prod _{p\mid q}\left( 1-\frac{1}{p^{2}}\right) ^{-1} \end{aligned}$$

where $A_q$ is defined in (2.10). Since $A_q$ is a multiplicative function of *q*, we can represent the *q*-sum as an Euler product, finding that

$$\begin{aligned} \frac{\mathfrak {S}_2}{\zeta (2)^2}= \prod _p \left( 1-\frac{1}{p^2}\right) ^2 \left( 1+\left( 1-\frac{1}{p^{2}}\right) ^{-1}\sum _{r\geqslant 1} \frac{A_{p^r}}{p^{6r}}\right) . \end{aligned}$$

We may now record the following result.

### Lemma 8.5

For any prime *p*, we have

$$\begin{aligned} \left( 1-\frac{1}{p^2}\right) ^2\left( 1+\left( 1-\frac{1}{p^{2}}\right) ^{-1}\sum _{r\geqslant 1} \frac{A_{p^r}}{p^{6r}}\right) = (1-p^{-1})(1-p^{-2})\tau _p. \end{aligned}$$

### Proof

We need to prove that

$$\begin{aligned} \left( 1-\frac{1}{p^2}\right) \left( 1+\left( 1-\frac{1}{p^{2}}\right) ^{-1}\sum _{r\geqslant 1} \frac{A_{p^r}}{p^{6r}}\right) = (1-p^{-1})\tau _p. \end{aligned}$$

But Lemma 7.13 implies that the left hand side is

$$\begin{aligned} 1-\frac{1}{p^2}+\sum _{r\geqslant 1} \frac{A_{p^r}}{p^{6r}}&=1-\frac{1}{p^2}+ \left( 1-\frac{1}{p}\right) ^2\sum _{r=1}^\infty \frac{\varrho (p^r)}{p^{3r}}-\frac{1}{p}\left( 1-\frac{1}{p}\right) \\&=\left( 1-\frac{1}{p}\right) \left( 1+\left( 1-\frac{1}{p}\right) \sum _{r=1}^\infty \frac{\varrho (p^r)}{p^{3r}} \right) . \end{aligned}$$

The desired equality now follows from Lemma 8.3. $\square$

Combining Lemmas 8.4 and 8.5 in (8.1), we therefore conclude that $c=c_V$, as claimed in Proposition  8.2, subject to the verification of Lemma 8.1.

### Proof of Lemma 8.1

Let $\textbf{y}\in {\mathbb {R}}^4$ and define

$$\begin{aligned} \varrho _\infty (\textbf{y})= \int _{-\infty }^\infty \int _{[-1,1]^{2}}{\text {e}}\left( \theta (x_{1}Q_{1}(\textbf{y})-x_{2}Q_{2}(\textbf{y}))\right) \textrm{d}\textbf{x}\textrm{d} \theta . \end{aligned}$$

Suppose first that $|Q_{1}(\textbf{y})||Q_{2}(\textbf{y})|>0$. Then

$$\begin{aligned} \begin{aligned}&\int _{[-1,1]^{2}}{\text {e}}\left( \theta (x_{1}Q_{1}(\textbf{y})-x_{2}Q_{2}(\textbf{y}))\right) \textrm{d}\textbf{x}\\&\quad = \prod _{i=1,2} \int _{-1}^{1}{\text {e}}\left( \theta x Q_{i}(\textbf{y})\right) \textrm{d}x = \prod _{i=1,2}\frac{\sin (2\pi \theta |Q_{i}(\textbf{y})|)}{\pi \theta |Q_{i}(\textbf{y})|}. \end{aligned} \end{aligned}$$

Hence it follows from [11, § 3.741] that

$$\begin{aligned} \begin{aligned} \varrho _\infty (\textbf{y})&= \frac{1}{\pi ^{2}|Q_{1}(\textbf{y})||Q_{2}(\textbf{y})|}\int _{-\infty }^{\infty }\frac{\sin (2\pi \theta |Q_{1}(\textbf{y})|)\sin (2\pi \theta |Q_{2}(\textbf{y})|)}{\theta ^{2}}\mathrm d\theta \\&=\frac{2\pi ^{2}\min _{i=1,2}|Q_{i}(\textbf{y})|}{\pi ^{2}|Q_{1}(\textbf{y})||Q_{2}(\textbf{y})|}\\&=\frac{2}{\max _{i=1,2}|Q_{i}(\textbf{y})|}. \end{aligned} \end{aligned}$$

Suppose next that $|Q_{1}(\textbf{y})||Q_{2}(\textbf{y})|=0$. But we clearly have $\max (|Q_{1}(\textbf{y})|,|Q_{2}(\textbf{y})|)>0$, since $Z({\mathbb {R}})= \emptyset$. It is easy to see that the argument goes through and leads to the exact same result.

Let $Y_2>Y_1\geqslant 1$. We may now conclude that

$$\begin{aligned} \int _{\begin{array}{c} \textbf{y}\in {\mathbb {R}}^4\\ Y_1 \leqslant |\textbf{y}|< Y_2 \end{array}} \frac{\textrm{d}\textbf{y}}{|\textbf{y}|^2\max (|Q_1(\textbf{y})|,|Q_2(\textbf{y})|)}= \frac{1}{2} \int _{\begin{array}{c} \textbf{y}\in {\mathbb {R}}^4\\ Y_1 \leqslant |\textbf{y}|< Y_2 \end{array}} \frac{\varrho _\infty (\textbf{y})}{|\textbf{y}|^2 }\textrm{d}\textbf{y}. \end{aligned}$$

Let us first consider the contribution from $\textbf{y}$ for which $|\textbf{y}|=|y_4|$. Writing $t_i=y_i/|y_4|$ for $1\leqslant i\leqslant 3$, we obtain the contribution

$$\begin{aligned} \int _{ Y_1}^{Y_2} \frac{\textrm{d} y_4}{y_4} \int _{[-1,1]^3} \varrho _\infty (t_1,t_2,t_3,1)\textrm{d}\textbf{t}= \log \left( \frac{Y_2}{Y_1}\right) \int _{[-1,1]^3} \varrho _\infty (t_1,t_2,t_3,1)\textrm{d}\textbf{t}, \end{aligned}$$

where $\textbf{t}=(t_1,t_2,t_3)$. On adding in the remaining three contributions, and observing that

$$\begin{aligned} \int _{[-1,1]^4}\varrho _\infty (\textbf{y})\textrm{d}\textbf{y}= \int _{[-1,1]^3} \varrho _\infty (t_1,t_2,t_3,1)\textrm{d}\textbf{t} +\cdots + \int _{[-1,1]^3} \varrho _\infty (1,t_2,t_3,t_4)\textrm{d}\textbf{t}, \end{aligned}$$

the statement of the lemma easily follows. $\square$
